# Transposable elements as drivers of genome evolution in *Drosophila virilis*

**DOI:** 10.1093/nar/gkag139

**Published:** 2026-02-17

**Authors:** Alexander P Rezvykh, Dina A Kulikova, Elena S Zelentsova, Liudmila Protsenko, Alina V Bespalova, Iuliia O Guseva, Justin P Blumenstiel, Mikhail B Evgen’ev, Sergei Y Funikov

**Affiliations:** Engelhardt Institute of Molecular Biology of Russian Academy of Sciences, Moscow 119991, Russia; Koltzov Institute of Developmental Biology, Russian Academy of Sciences, 119334 Moscow, Russia; Engelhardt Institute of Molecular Biology of Russian Academy of Sciences, Moscow 119991, Russia; Engelhardt Institute of Molecular Biology of Russian Academy of Sciences, Moscow 119991, Russia; Engelhardt Institute of Molecular Biology of Russian Academy of Sciences, Moscow 119991, Russia; Moscow Center for Advanced Studies, Kulakova Str. 20, 123592 Moscow, Russia; Department of Ecology and Evolutionary Biology, University of Kansas, Lawrence, Kansas 66049, United States; Engelhardt Institute of Molecular Biology of Russian Academy of Sciences, Moscow 119991, Russia; Institute of Evolution, University of Haifa, Haifa 3498838, Israel; Engelhardt Institute of Molecular Biology of Russian Academy of Sciences, Moscow 119991, Russia

## Abstract

Transposable elements (TEs) drive genomic innovation, but their dynamics in non-model species remain unclear. Here, we integrated multi-omics data to explore TE dynamics in *Drosophila virilis*, an important model for repetitive DNA research. By combining computational predictions with manual curation, we identified 100 TE families and delineated three temporal waves of TE mobilization: recent activity, speciation-associated, and ancient invasions. TEs in *D. virilis* dynamically colonise both euchromatin and heterochromatin, suggesting heterochromatin is not solely a repository for degenerate repeats. While most TEs are widespread across strains, some exhibit strain-specific expansions, indicating varied activity and silencing. We found substantial evidence for horizontal transfer of TEs among close relatives, demonstrating that the *D. virilis* species group functions effectively as a TE “ecosystem”, allowing for recurrent invasion, loss, and re-invasion of TE lineages across the group. Epigenetic profiling revealed that H3K9me3 spreading from TEs represses adjacent genes in a distance-dependent manner, influenced by insertion length and genomic context, affecting developmental and metabolic genes. We also discovered the first spontaneous polymorphic inversion in *D. virilis* linked to retrotransposons. Our findings illuminate TEs as drivers of genomic innovation, influencing gene regulation and evolutionary trajectories, providing a framework for studying TE dynamics across animal species.

## Graphical Abstract

**Graphical Abstract:**
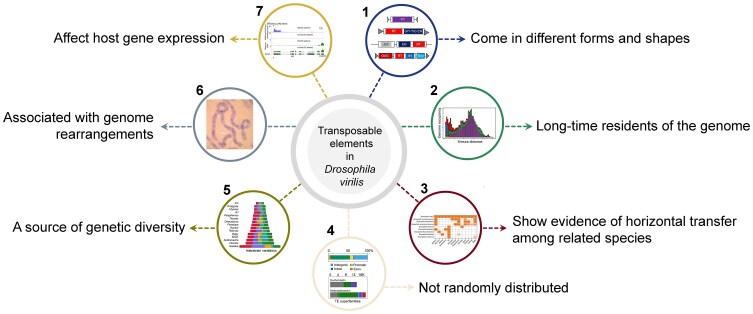

## Introduction

Transposable elements (TEs) are mobile DNA sequences that can replicate and change their genomic position, allowing them to constitute a major fraction of eukaryotic genomes [1–4]. Once considered “junk DNA”, these elements are now recognized as crucial drivers of genome plasticity, evolution, and regulation in eukaryotes [5–7].

The fundamental classification of eukaryotic TEs distinguishes two major classes based on their transposition intermediate: class I—retrotransposons, and class II—DNA transposons [8, 9]. Retrotransposons differ by the presence or absence of long terminal repeats (LTRs). LTR retrotransposons typically encode *gag* and *pol* polyproteins, with *pol* including reverse transcriptase (RT), integrase (IN), protease, and RNase H domains [3, 4]. Certain LTR retroelements may additionally encode a retroviral envelope protein *env*. This particular class of LTR retrotransposons is currently more widely recognized as endogenous retroviruses (ERVs) [9]. Non-LTR retroelements, such as long interspersed nuclear elements (LINEs), possess a more elementary structural configuration but encode polyproteins that exhibit endonuclease (EN) and RT activities, the latter of which is a prerequisite for target-primed reverse transcription [10]. *Penelope*-like elements (PLEs) form a distinct monophyletic clade possessing a pseudo-LTR and a GIY-YIG EN domain which is not shared with any other retroelement subclasses [11–13]. Class II TEs includes three major groups: transposase-coding elements (e.g. *Tc1*-*Mariner, Cryptons*), rolling-circle elements (RC, *Helitrons*), and self-synthesizing *Mavericks* [14, 15]. Most class II elements have terminal inverted repeats (TIRs) recognized by transposase, with the exception of *Helitrons* and *Cryptons*. TEs can be further divided into superfamilies and families, that are more accurately characterized in terms of phylogenetic relationships and DNA sequence conservation [9].

To control TE activity, hosts employ small RNA-based silencing strategies. In metazoan germline, a distinct class of small RNAs called Piwi-interacting RNAs (piRNAs) plays a crucial role in the silencing of TEs [16, 17]. Regulation of TEs in the germline is of paramount importance, as only TE insertions present in gametes can contribute to the spread of TEs in natural populations. In Drosophila, piRNA silencing relies on three Argonaute proteins: Piwi, Aubergine (Aub), and Argonaute-3 (Ago3), that are loaded with TE-derived piRNAs to mediate co-transcriptional (Piwi) and post-transcriptional repression (Aub/Ago3) [18]. The majority of piRNAs are produced by piRNA clusters, usually located in the pericentromeric and subtelomeric regions of chromosomes and composed of diverged copies of TEs [19, 20]. In both germ cells and the surrounding somatic cells of the Drosophila ovary, piRNA-Piwi complexes silence TEs by inducing changes in chromatin state, including the deposition of the heterochromatic histone mark H3K9me3 [21, 22]. H3K9me3 plays a dual role: it not only reinforces TE silencing but also facilitates the transcription of piRNA precursors, effectively transforming targeted TE insertions into piRNA clusters [23–25].

TE-host interactions represent a balance between genomic instability and innovation. TE insertions can disrupt genes or regulatory regions, and their epigenetic silencing can have unintended consequences [26]. Specifically, the repressive histone mark H3K9me3 can “spread” from silenced TEs into adjacent genes, potentially repressing their expression [27–31]. While this often leads to purifying selection against such insertions [26, 32], it can also be a source of genetic diversity and adaptation [33–36].

Even with robust repressive mechanisms in place, forcing TEs to exist in a transposition-selection balance, TEs persist as one of the most dynamic and rapidly evolving components of eukaryotic genomes. TE content can differ drastically even between closely related species [37–39] and contribute to intraspecific variation [29, 40–42]. In comparison to the animal and plant genomes [43, 44], insects, particularly Drosophila, exhibit remarkable TE diversity and are excellent models for studying TE dynamics [45–47].

The *Drosophila virilis* species group has emerged as a valuable model for comparative genomics and evolution, offering a robust framework for the study of repetitive DNA due to abundant diversity of interspersed and tandem repeats [48–53]. The *virilis* species group of Drosophila includes 12 species and is divided into three subgroups, or phylads, *virilis, littoralis*, and *montana* diverged ∼9 million years ago (mya) [54–56]. Within the *virilis* phylad, *D. virilis* is the earliest diverging lineage, having split from the ancestor of *D. lummei* and *D. americana/D. novamexicana* about 5 mya [54, 55]. *Drosophila lummei* subsequently diverged about 3 mya, with *D. americana* and *D. novamexicana* only diverging from each other about 1 mya [55, 56]. *Drosophila virilis* harbours a diverse array of TEs, including the *Penelope* retroelement, which exhibits active transposition implicated in hybrid dysgenesis, a syndrome of hybrid sterility and germline damage [57, 58]. Furthermore, studies in *D. virilis* have revealed lineage-specific TE expansions, thereby providing evolutionary context for TE diversification and TE silencing mechanisms [20, 59, 60]. Classical cytogenetic studies of the *virilis* species group demonstrated that *D. virilis* karyotype is the most primitive and probably ancestral among the *virilis* phylad based on chromosomal phylogenies [54, 61].

In this study, we employed a multi-omics approach across several *D. virilis* strains to investigate TE dynamics. We generated a refined, curated TE library and used it to delineate three temporal waves of TE mobilization. We show that TEs dynamically colonize both euchromatin and heterochromatin, exhibit strain-specific expansions, and are regulated by a dynamic piRNA response. Furthermore, we demonstrate that TE insertions can suppress nearby gene expression via H3K9me3 spreading in a distance- and context-dependent manner. Finally, we report the first polymorphic inversion in *D. virilis*, likely linked to retrotransposon activity.

## Materials and methods

### Fly stocks and husbandry

Five *D. virilis* strains were analysed in this study, including three wild-type strains *9* (Batumi, Georgia), *101* (Japan, neutral substrain according to the classification used in hybrid dysgenesis studies) [62], *15010-1051.47* (Hangchow, China), and two laboratory strains with recessive markers, *140* (*eb va*) and *160* (*b; tb gp; cd; pe; gl*). Fly stocks of strains *9, 140*, and *101* were obtained from the Stock Centre of Koltzov Institute of Developmental Biology RAS (Moscow, Russia). Strain *160* is a long-established strain maintained in our laboratory and has the capacity to induce hybrid dysgenesis [58]. Strain *15010-1051.47* was obtained from the National Drosophila Species Stock Centre at Cornell University (USA). Flies were reared on a standard medium at 25°C until sexual maturity (10–15 days).

### DNA extraction, library preparation, ONT sequencing, and genome assembly

The large size of the *D. virilis* genome (∼390 Mb) is primarily due to the expansion of pericentromeric/centromeric 7 bp satellite DNA [63]. To enrich for euchromatic sequences, we isolated genomic DNA from ovarian tissue, where centromeric satellite DNA is under-replicated due to polytenization [64]. Genomic DNA was isolated from thirty pairs of ovaries from 10–15-day-old females. Ovaries from strains *160, 101*, and *1051.47* were collected for DNA extraction. Libraries were prepared with the ONT SQK-LSK109 kit and sequenced on FLO-MIN-106D flow cells. Basecalling was performed with Guppy 6.4.6, followed by quality filtering [65] and adapter removal [66].

Genomes were assembled using Flye v2.9 [67]. Quality was evaluated using QUAST 5.1 [68], and BUSCO (Diptera lineage) [69]. The *D. virilis* r.1.06 genome [70] was used for reference-based QUAST assessment, and annotation transfer using Liftoff [71].

Genome assemblies for strains *101* and *1051.47* are available from the NCBI BioProject database under accession number PRJNA1279437. The genomes of the *160, 9*, and *140* strains, which had been previously sequenced and assembled [72–74], were retrieved from NCBI (GCA_007989325.2 – strain *160*; GCA_016920725.1 – strain *9*; GCA_050656195.1 – strain *140*).

To compare PacBio and ONT sequencing errors, we aligned sequenced genomes to a reference (strain *9*) using GSAlign [75]. SNPs were identified and annotated within gene loci using R packages VariantAnnotation and GenomicFeatures [76, 77].

To compare homozygosity and heterozygosity levels, reads were aligned to the *D. virilis* (strain *101*) genome using BWA-MEM [78]. Alignments were processed with SAMtools [79]. Variant calling was performed using FreeBayes [80].

This study used ONT reads from strain *160* (for which a PacBio chromosomal assembly is available [73]) for *de novo* TE insertion analysis and SNP calling using FreeBayes.

### *De novo* annotation of TEs and creation of a curated TE library

We performed *de novo* annotation of TEs in the *D. virilis* genomes using RepeatModeler2 [81] with the “-LTRstruct” parameter. The output was filtered to remove non-TE sequences (e.g. ribosomal RNA, numts) and short sequences [82], though LTR elements were exempt from length-based filtering to preserve structural accuracy. “Unknown” sequences from RepeatClassifier were discarded.

Further curation involved aligning the filtered output to existing TE libraries [83, 84] using blastn [85]. Shorter, redundant sequences were removed. Remaining sequences were manually curated and extended using established guidelines [86, 87] and scripts from Storer *et al.* [88]. A final manual curation involved multiple sequence alignment of full-length copies and annotation of protein domains using the Conserved Domain Database [89]. The resulting library consists of 100 TEs: 14 DNA, 5 RC, 30 LINE, and 50 LTR elements, and 1 PLE (Supplementary File 1). To avoid confusion, the names of the TEs described in this study and their corresponding homologous families in RepBase and Erwin *et al.* [84] are provided in Supplementary File 2.

### TE analysis

To screen genomes for TE insertions, we used RepeatMasker (v 4.1.0) [90] in RMBlast mode. The output was filtered using two criteria: (i) >90% identity over >50 bp, and (ii) <50% overlap with low-complexity regions; only insertions >50 nt were retained. We used the script “one_code_to_find_them_all.pl” [91] with the parameter “–insert 11”.

Divergence times were estimated by calculating Kimura distance from RepeatMasker alignments using a modified “calcDivergenceFromAlign.pl” script. The resulting distance matrix was Z-transformed and clustered via k-means, with the optimal cluster number determined using the factoextra R package [92].

The potential for transposition was assessed by identifying open reading frames (ORFs) [93]. TE sequences were analysed using ORFik [94] and Biostrings [95]; ORFs starting with ATG were translated. Proteins were clustered with CD-HIT (90% similarity). Insertions encoding a protein ≥ 90% of the length of the canonical sequence were considered full-length.

Pericentric heterochromatin was annotated in Integrative Genomic Viewer (IGV) [96] using ChIP-seq data for the heterochromatic mark H3K9me3 from ovarian tissue [97].

For phylogenetic analysis, transposase (DNA transposons) or RT (retroelements) encoding ORFs were aligned using MAFFT [98], trimmed with trimAl [99], and analysed with ProtTest 3 [100]. Phylogenetic trees were inferred using the maximum likelihood method in MEGA 11 [101]. Visualization was performed using iTOL v6 [102].

The ggplot2 package [103] was used to visualize the distribution of TEs across the genome. The custom scripts used in this study are available on https://github.com/aprezvykh/drosophila_virilis_TE_scripts.

### Identification of horizontal transfer of TEs

To identify horizontal transfer (HT) of TEs in *D. virilis*, we searched for near-identical TEs in divergent relatives [104, 105]. We used *D. virilis* TE sequences as queries in a blastn search against the eight *D. virilis* group genomes assembled to date. Hits were filtered for ≥ 99.5% sequence identity and ≥ 90% query coverage of the canonical TE length.

To distinguish HT from vertical inheritance, we compared the synonymous substitution rate (dS) of TEs with that of 50 single-copy orthologues from BUSCO [69]. Alignments were generated using MAFFT [98] and dS was calculated using MSA2dist [106]. The dS distributions of genes and TEs were compared with a Mann–Whitney U test (False Discovery Rate (FDR) corrected).

### Identification of nonreference TE insertion

Using TELR [107], we identified nonreference TE insertions in *D. virilis* strains by mapping ONT reads from strains *9, 101, 140*, and *1051.47* to the strain *160* reference genome. To identify nonreference TE insertions in strain *160*, we used TELR with ONT reads from *160* as a query and the *1051.47* assembly as a reference.

To identify unique TE insertions for each strain, we compared the coordinates of insertions obtained from TELR using the “bedtools intersect” function [108]. Genomic feature distribution was analysed with ChIPSeeker’s “annotatePeak” function [109].

### RNA-seq experiments and TE expression analysis

Total RNA was extracted from 10–15-day-old *D. virilis* ovaries using Extract RNA reagent (Evrogen, Russia). RNA quality (RNA Integrity Number, (RIN) ≥ 8) was confirmed using an Agilent BioAnalyzer 2100. Libraries were prepared from poly(A)-selected RNA using the NEBNext Ultra II Directional RNA Library Prep Kit (NEB, USA) and sequenced on an Illumina NextSeq 2000 (2 × 50 bp; two biological replicates per strain).

Reads were trimmed with TrimGalore and aligned to the genome using STAR 2.7.1a [110]. TE expression analysis was conducted using TEtranscripts [111] with the TE annotation file generated by RepeatMasker. The counts were then normalized to RPKM (Reads Per Kilobase per Million mapped reads). Differential expression of protein-coding genes was analysed with edgeR [112].

### Small RNA-seq experiment and TE-targeting piRNA analysis

The ovarian small RNA fraction for cloning was separated from total RNA (∼15 μg) of flies aged 10–15 days using 15% polyacrylamide gel electrophoresis containing 8M urea. Subsequently, gel fragments corresponding to the small RNA fraction were excised using chemically synthesized RNA oligonucleotides corresponding to 21 and 29 nucleotides as size markers. Small RNA libraries were then prepared using the Illumina TruSeq Small RNA Library Prep Kit (Illumina, USA) according to the manufacturer’s protocol.

Following adapter trimming, reads matching ribosomal RNA, tRNA, small nucleolar RNA (snRNA), and microRNA were removed. The remaining reads were mapped to *D. virilis* TEs and genome using Bowtie [113], with a requirement for a perfect match (0 mismatches). We considered small RNAs 23–29 nt in length as piRNAs, consistent with known piRNA characteristics (1U bias, ping-pong signature) [19, 20]. Analysis of piRNAs, including sorting of sense and antisense piRNAs and calculation of ping-pong signatures, was carried out by well-described techniques [114] and custom scripts written in Python.

### ChIP-seq experiments and enrichment analysis of methylated H3K9

ChIP-seq for H3K9me3 and H3K9me2 was performed as described [115] using antibodies ab8898 and ab1220 (Abcam). Libraries were prepared using the NEBNext Ultra II Kit (NEB, USA). Paired-end sequencing (50 + 50 bp) was conducted on an Illumina NextSeq 2000 platform. For analysis of H3K9me3 enrichment in strains *160* and *9*, we used data (single-end sequencing) published previously [97].

Reads were aligned to the genome using Bowtie2 [116], processed with SAMtools [117] to filter multimapped reads and remove duplicates, and converted to normalized BigWig files using deepTools2 [118]. Enrichment was calculated by subtracting input signals and generating matrices centred on TE insertions.

To assess the epigenetic impact of unique TE insertions, the H3K9me2/me3 signal in a strain with an insertion (experimental, TE present) was compared to the mean signal from four strains without it (control, TE absent). Enrichment in the 1-2 kb regions flanking the TE was tested for statistical significance using the Mann–Whitney U test (FDR < 0.05). The association between heterochromatin spreading and gene downregulation was assessed using a chi-squared test. Significant loci were manually curated in IGV.

### Chromosomal inversion analysis

Chromosomal inversion analysis was performed using pairwise genome-wide alignment with Minimap2 (asm5 preset) [119], visualized with dotPlotly (https://github.com/tpoorten/dotPlotly), and refined with blastn for breakpoint accuracy.

Polytene chromosomes from F1 hybrids (*1051.47* × *9* strains) were prepared and stained as described [120].

Polymerase chain reaction (PCR) validation of the inversion breakpoints was performed using primers: P1 – GCGTTTGTTCGCCAAAGCG; P2 – GTCCTGCCAGTTGTTTCAGTTTG; P3 – CATTATAATCTTGCAGCTGCC; P4 – CCGTTAAACTGAATTATGCGCCATG.

**The custom scripts used in this study are available in https://github.com/aprezvykh/drosophila_virilis_TE_scripts**.

**Detailed experimental procedures are provided in the Supplementary Materials**.

## Results

### Whole-genome sequencing and assembly of *D. virilis* strains of geographic and laboratory origin

To study the diversity of TEs, we utilized genomic assemblies of three *D. virilis* strains that had been published previously [72–74]. In addition, we applied long-read sequencing to generate whole-genome assemblies of two *D. virilis* strains. All five *D. virilis* strains examined originated from diverse sources spanning geographic populations and laboratory lineages: three wild-type strains from the Republic of Georgia (strain *9*), Japan (strain *101*), and China (*1051.47*), and two laboratory strains, *140* and *160*.

Although genome assemblies differed in contig length and number, the N50 was high across all five genomes, ranging from 16.9 Mb in strain *9* to 31 Mb in strain *160* (Table 1). The total length of the genome assemblies ranged from 165.5 Mb in strain *101* to 182.3 Mb in strain *9*. The BUSCO percentage of complete orthologues of dipteran genes was similar across the assemblies (99.5%–99.6%) (Table 1). Thus, the analysed genomes are comparable in terms of contiguity, total length, and completeness.

**Table 1.** Genome assembly statistics for the five *D. virilis* genomes studied in this work

Genome	Assembly length (Mb)	Number of contigs	Contigs N50 (Mb)	GC (%)	BUSCO complete (%)	BUSCO single (%)	BUSCO duplicated (%)
*160*	169.7	45	31	40.4	99.6	99.1	0.5
*9*	182.3	567	16.9	40.5	99.5	99.1	0.4
*101*	165.5	155	28.9	40.4	99.5	99.2	0.3
*140*	168.2	219	27.3	40.3	99.6	99.3	0.3
*1051.47*	174.9	225	27.5	40.4	99.6	99.3	0.3

Genomes of strains *160* and *9* were assembled previously and deposited in GenBank under the numbers GCA_007989325.2 and GCA_016920725.1, respectively. The genomes of strains *101, 140*, and *1051.47* were sequenced and assembled in the current study.

We have also performed SNP analysis to determine the levels of heterozygous and homozygous mutations in the studied strains. The analysis indicated that strain *160* has a higher level of heterozygous SNPs than the other strains analysed (Supplementary Fig. S1A).

### Expansion and refinement of TEs in *D. virilis* and creation of a curated library of canonical TE sequences

**Figure 1.**
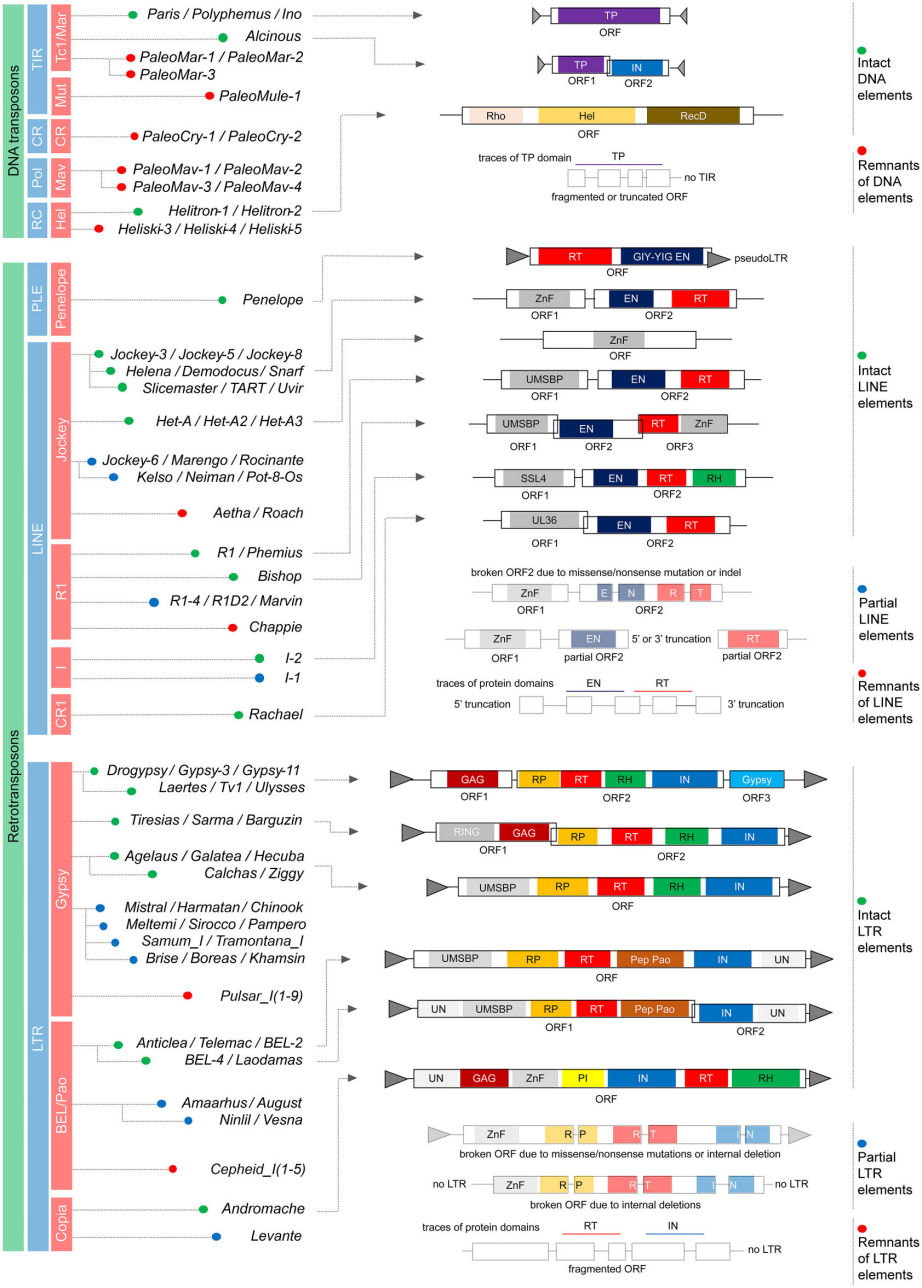
A guide to the TEs of *D. virilis*. The **left** panel lists all annotated TE families in *D. virilis* in terms of their taxonomy based on phylogenetic analysis and core protein homology with major TE superfamilies and subclasses. The **right** panel depicts genetic structures of representative elements from each TE family. Outlined boxes are ORFs, coloured regions are protein domains, triangles are repeated sequences (TIRs and LTRs). Element lengths are not to scale. The use of green and red circles is intended to denote intact and severely truncated (remnants) canonical copies of the elements, respectively. Blue circles indicate retroelements with partial reconstruction of their canonical copies. Abbreviations: TIR – Terminal Inverted Repeat-containing transposons; CR – *Cryptons*; Pol – *Mavericks/Polintons*; RC – rolling circle replication elements (*Helitrons*); PLE – *Penelope*-like elements; LINE – Long Interspersed Nuclear Elements; LTR – Long Terminal Repeat-containing retrotransposons; UN – The TE superfamily has not been identified/domain with unknown function; ORF – open reading frame; TP – transposase; IN – integrase; Rho – transcription termination factor Rho; Hel – helitron helicase-like domain; RecD – helicase subunit RecD; RT – reverse transcriptase; EN – endonuclease; GIY-YIG EN – GIY-YIG endonuclease; ZnF – Zn finger; UMSBP – universal minicircle sequence binding protein; SSL4 – superantigen-like protein SSL4; UL36 – large tegument protein UL36; GAG – retrotransposon gag protein; Gypsy – Gypsy protein; RING – ring finger domain; RP – retropepsin; RH – ribonuclease H domain; Pep Pao – Pao retrotransposon peptidase; PI – GAG-pre-integrase domain.

To establish a comprehensive and high-quality TE library for *D. virilis*, we integrated computational predictions from RepeatModeler2 [81] with existing databases and extensive manual curation (see the ‘Materials and methods’ section for details). This consolidated library comprises 100 distinct TE families, including 14 DNA transposons, 5 RC/Helitrons, 30 LINEs, 50 LTR retrotransposons, and 1 PLE (Fig. 1). Given the structural features inherent to TEs across diverse subclasses, the reconstruction of full intact canonical TE sequences was successful for 44 elements, including 4 DNA transposons, 2 RC elements, 1 PLE element, 17 LINE retrotransposons, and 20 LTR retrotransposons (marked with green circles in Fig. 1).

Following the reconstruction of the consensus TE copies, the phylogenetic analysis was performed using TE sequences of invertebrates from RepBase v27.01 [83] (Supplementary Figs S2–S5). This approach enabled us to determine that the four described intact transposase-coding DNA elements belong to the *Tc1*-*Mariner* superfamily (Fig. 1 and Supplementary Fig. S2). The analysis of the 17 intact LINE elements demonstrated that 12 belong to the *Jockey* superfamily, three to the *R1* superfamily, and one each to the *I* and *CR1* superfamilies (Fig. 1 and Supplementary Fig. S3). Of the three superfamilies of LTR retrotransposons typically present in Drosophila genomes [3], 14 intact elements belong to the *Gypsy* superfamily, five to the *Bel/Pao* superfamily, and one represents the *Copia* superfamily (Fig. 1 and Supplementary Fig. S4). Finally, the other two intact DNA transposons belong to the *Helitron* group (Fig. 1 and Supplementary Fig. S5).

The remaining TE families are represented in an incomplete form, exhibiting varying degrees of truncation. For instance, LINE retroelements and LTR retrotransposons frequently exhibit a 5′ or 3′ truncation, likely resulting from deletion or recombination [14, 15]. As a result, essential domains like IN or RT are frequently missing, compromising the element’s basic structure. Retrotransposons *Amaarhus, Kelso, Rocinante, Neiman, I-1, Levante, Harmatan, Mistral, Meltemi, Sirocco, Pampero, Khamsin, Ninlil, Chinook*, etc., represent just such cases (marked with blue circles in Fig. 1). Despite our efforts, we were unable to reconstruct a complete consensus sequence for these TE families. Thus, these elements were designated as “partial” for further analysis.

As TEs are long-term residents of eukaryotic genomes, particularly in Drosophila, it is often only possible to identify traces of protein domains that were once present. An even more challenging scenario arises when only partial homology with a known TE allows for the tentative classification of a TE into a specific superfamily. For instance, DNA transposons belonging to *Polinton* and *Crypton*, as well as LTR retrotransposon families such as *Pulsar-1-9* and *Cepheid-1-5*, represent this group of TEs in the *D. virilis* genome. We designated such elements as “remnants” (marked with red circles in Fig. 1).

In this study, we re-annotated all TEs in *D. virilis*, resulting in a refined library of 100 TE sequences. While only 44 intact TEs were fully reconstructed, the remaining elements, classified as partial (26 elements) and remnants (30 elements), provide valuable insights into the evolutionary dynamics of TEs in *D. virilis* species.

### Divergence landscape analysis reveals temporal patterns of TE mobilization in *D. virilis*

To investigate the evolutionary history of TE activity, we generated divergence landscapes based on Kimura distance for TE copies in the genome of the *D. virilis* strain *160*, which was assembled at the chromosomal level. The estimated Kimura distances were converted into a data matrix, followed by k-means clustering (see the ‘Materials and methods’ section for details). This approach enabled the identification of three discrete TE clusters that reflected the divergence times of the TE families occupying the *D. virilis* genome.

**Figure 2.**
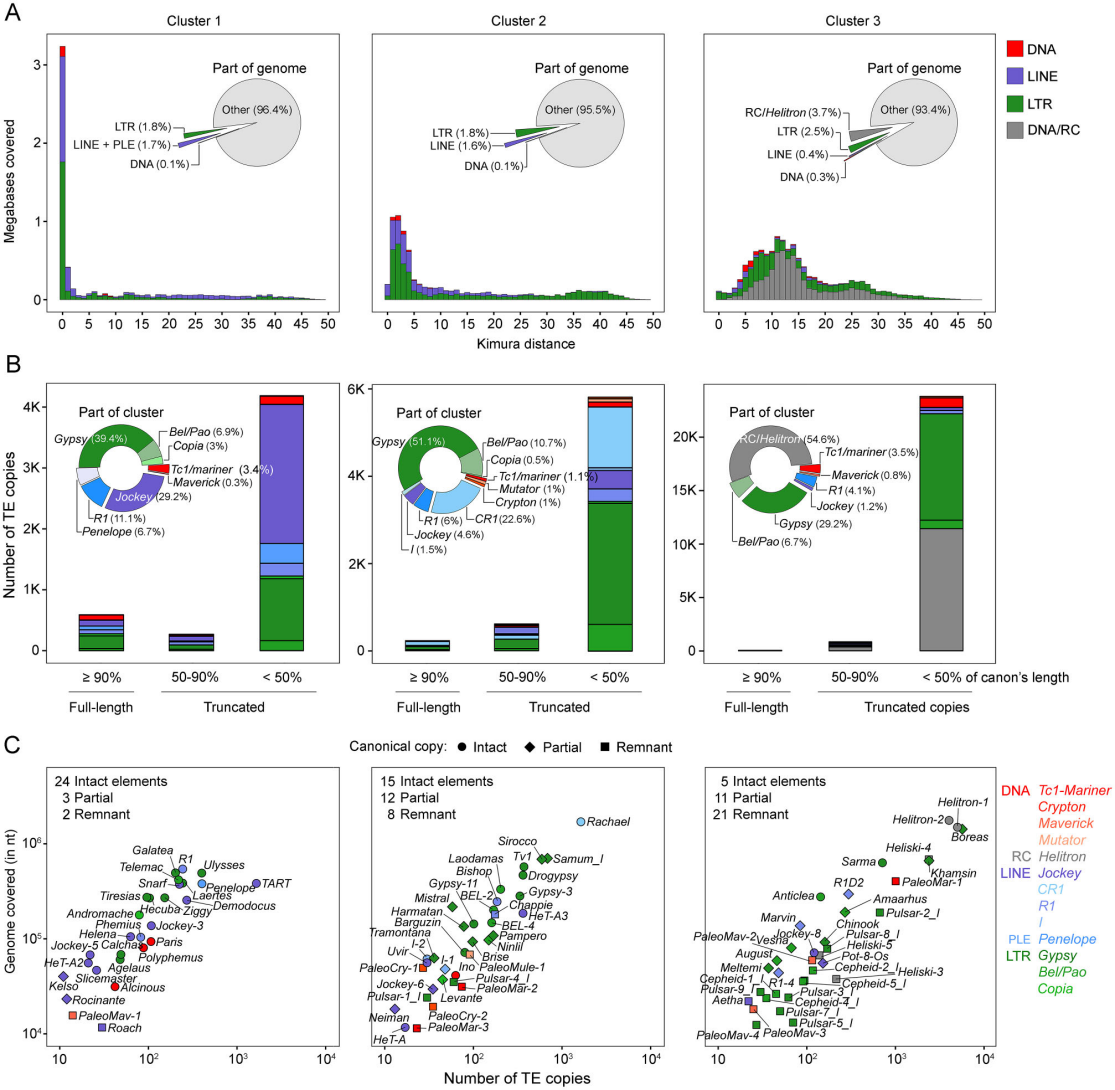
Divergence and occupancy of TEs in the *D. virilis*. (**A**) TE divergence landscape. The X-axes show the level of divergence (Kimura substitution level) between each identified TE copy and the consensus sequence for that TE family. The Y-axis shows the part of the genome occupied by each bar (in millions of bases covered). The number of TE clusters was determined by the silhouette method, followed by k-means clustering. Colours represent TE subclasses. (**B**) The number of TE copies in each cluster, as defined in panel (A), is categorized according to their length relative to the canonical copy. Colours show TE superfamilies. Partial elements according to the structure of their canonical copies were considered only as truncated copies. Remnant elements were considered only as half-truncated (<50% of the canonical copy length). (**C**) The relationship between the number of TE copies and the number of covered bases in the genome is given for each TE family. Colours show TE superfamilies. Intact, partial, and remnant TEs according to their canonical copies are indicated by circles, rhombuses, and squares, respectively. Data were analysed on the chromosome assembly of strain *160*.

The first cluster predominantly includes copies of LTR, LINE, PLE, and DNA transposons with low or no divergence from the consensus sequences (K-value ≤ 1). These TE copies occupy 3.6% of the genome (Fig. 2A). Assuming a molecular clock for nucleotide substitutions within TE copies, these elements represent amplification of TE copies that bear a high degree of similarity to their progenitor copy, consistent with recent transposition of an active element. A broader distribution is observed around K values of 0-5 in the second cluster, which likely reflects TE transpositions during the divergence of the *D. virilis* species group (Fig. 2A). TE copies in this cluster occupy 4.5% of the genome (Fig. 2A). Finally, the third cluster, comprising ancient historical TE transpositions, has K values ranging from 5 to 30 (Fig. 2A). Two waves of TE invasion can be distinguished in this pattern, occurring at K values of around 11 and 25. Comprising the largest portion of the genome (6.6%), the third cluster is dominated by *Helitrons* and LTR retrotransposons belonging predominantly to partial and remnant families (Fig. 2A).

We next assessed the structural integrity of TEs within these clusters. A substantial number of TEs accumulated in the genome of *D. virilis* in a truncated form with low nucleotide substitution from the consensus (Supplementary Fig. S6). Given this, we categorized TEs from intact families as “full-length” if they were ≥90 percent of the consensus length. Shorter copies were considered “truncated”. Given that, a complete canonical copy was recovered only for 44 TE families, the above criterion was applied here and below exclusively to these intact TE families. The remaining partial and remnant TE families were examined only as truncated copies. The distribution of full-length and truncated TE copies differed significantly between clusters (Fig. 2B). As Kimura distance increased, the number of full-length insertions decreased, while the number of truncated copies increased. Cluster 1 was dominated by ∼600 full-length copies, cluster 2 contained ∼250, and cluster 3 contained very few. Conversely, the number of truncated copies was minimal in cluster 1 (>4000), rose sharply in cluster 2 (>6000), and increased dramatically in cluster 3 (over 22 000) (Fig. 2B).

Regarding TE families, more than half of the intact TE families (24 out of 44) were assigned to the first cluster, along with several partial and remnant elements. This suggests that these elements are the youngest and most transpositionally active residents of the *D. virilis* genome (Fig. 2C). These active families include members of the *Gypsy* (e.g. *Ulysses, Galatea*), *Jockey* (e.g. *Demodocus, Snarf*), and *R1* (e.g. *R1, Phemius*) superfamilies, as well as *Tc1-Mariner* DNA transposons (e.g. *Paris, Polyphemus*) and *Penelope* (Fig. 2C). A second cluster also contained many intact TEs [15], but included more partial [12] and remnant [8] elements, suggesting lower transpositional activity (Fig. 2C). While LTR retroelements dominate these clusters, the *CR1* superfamily (e.g. *Rachael*) is exceptionally abundant, with over 1600 copies in the genome (Fig. 2B and C). The third cluster consists mainly of partial and remnant TEs (∼89% of the elements in the cluster), representing traces of ancient invasions (Fig. 2C). In contrast to the first two clusters of predominantly intact elements, this cluster still contains some intact *Helitrons*, LTRs, and LINEs. Notably, *Helitron-1* and *Helitron-2* represent over half of the ancient TEs, but only a few full-length copies remain (Fig. 2C).

To control for sequencing technology bias (PacBio for strain *160* and ONT for other strains), we confirmed that the Kimura distance distributions and three-cluster structure were consistent across all strains (Supplementary Fig. S7).

In summary, the divergence landscape analysis of TEs in *D. virilis* revealed distinct evolutionary waves of TE activity, categorized into three clusters based on Kimura distance and copy integrity: recent, speciation-associated, and ancient.

### TE dynamics across chromatin domains provide evidence of heterochromatic and euchromatic colonization in *D. virilis*

Transposon occupancy varied accordingly with the total length of the genome assembly and ranges from 11.8% in strain *101* (the shortest assembly, 165.5 Mb) to 18.8% in strain *9* (the longest assembly, 182.3 Mb; Table 1 and Supplementary Table S1). In the chromosomal assembly of strain *160*, TEs occupied just over 14% of the *D. virilis* genome (Fig. 2A and Supplementary Table S1). Notably, the TE load was estimated for the entire genomes assembled so far, encompassing both euchromatin and heterochromatin regions, that are known to differ significantly in TE density [121].

To separate the TE content in euchromatin from that in pericentromeric heterochromatin, we analysed the TE load in the chromosome assembly of strain *160*. The percentage of euchromatin regions of each chromosome comprised by TEs ranged from 3.09% (in chromosome 3, Muller D) to 4.65% (in chromosome 2, Muller E; Supplementary Table S1). The average TE density was 0.74 for DNA transposons, 1.18 for LINE elements, 2.37 for *Penelope*, 1.61 for LTR elements, and 13.87 for RC elements per million bases (Mb) across all chromosomes (Supplementary Table S2). The density of RC elements was higher than that of other TE subclasses, indicating that RC elements are the most prolific residents of the *D. virilis* genome.

**Figure 3.**
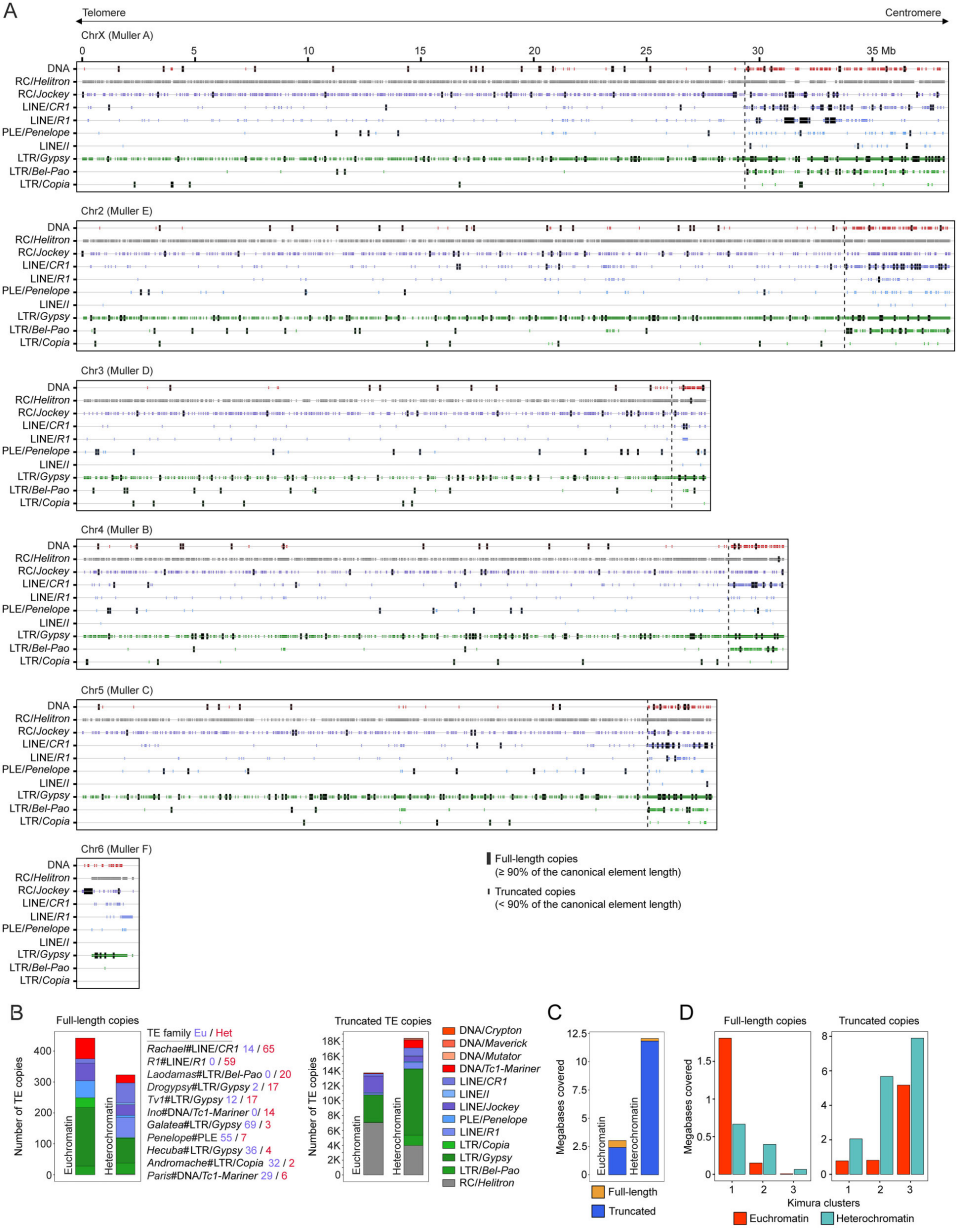
TE landscape in the genome of *D. virilis*. (**A**) Distribution of TEs along chromosomes. Long vertical dashes indicate full-length TE copies (≥90% of the canonical copy length), short vertical dashes indicate truncated TE copies (<90% of the canonical copy length). Dotted vertical lines indicate the boundary between euchromatin and pericentromeric heterochromatin. (**B**) The bar plots show the number of full-length and truncated TE copies located on different sides of the euchromatin-heterochromatin boundary. For full-length TE copies, the most contrasting TE families are indicated on the right according to their predominant presence either in euchromatin or in pericentromeric heterochromatin. (**C**) The bar plots show the occupancy of TE copies in both chromatin types. (**D**) Association between Kimura divergence clusters and chromatin states (euchromatin versus heterochromatin). See also Supplementary Tables S1 and S2. Data were analysed on the chromosome assembly of strain *160*.

The key distinction in TE distribution lies not in their sheer abundance, but in their structure and genomic location. Although all TE superfamilies were detected in pericentromeric heterochromatin (Fig. 3A) and the total count of truncated copies showed minimal discrepancy between the two chromatin types (∼14 000 copies in euchromatin versus ∼18 000 in heterochromatin; Fig. 3B), their genomic impact differed significantly. Euchromatin was preferentially enriched for full-length TE copies, while heterochromatin is predominantly occupied by truncated copies (χ^2^ test, *P*-value <.001) (Fig. 3C).

However, a higher proportion of full-length TE copies from several TE families was observed in heterochromatin compared to euchromatin, and a few TE families were located exclusively in heterochromatin. For instance, the number of full-length copies of the LINE element *Rachael*, as well as the LTR elements *Tv1* and *Drogypsy*, was 1.5–4 times greater in heterochromatin than in euchromatin (Fig. 3B). Furthermore, the *Laodamas* and *R1* retroelements, as well as the *Ino* DNA transposon, were represented in the *D. virilis* genome as full-length copies exclusively in pericentromeric heterochromatin (Fig. 3B). It should be noted, however, that satellite arrays are under-represented due to sequencing DNA from ovaries where centromeric regions is under-replicated due to polytenization.

Analysis of full-length TE copies revealed a distinct distribution pattern based on Kimura divergence clustering. Recent TEs (cluster 1) preferentially occupied euchromatin (χ^2^ test, *P*-value <.001), while both speciation-associated (cluster 2) and ancient (cluster 3) TEs were highly associated with heterochromatin (χ^2^ test, *P*-value <.01) (Fig. 3D).

In summary, TEs in *D. virilis* actively colonize both euchromatic and heterochromatic regions. While euchromatin was enriched for young, full-length copies, heterochromatin also hosted a significant number of intact elements, demonstrating that it is not merely a passive repository for degenerate repeats.

### TE diversity and intraspecific variation in *D. virilis*

**Figure 4.**
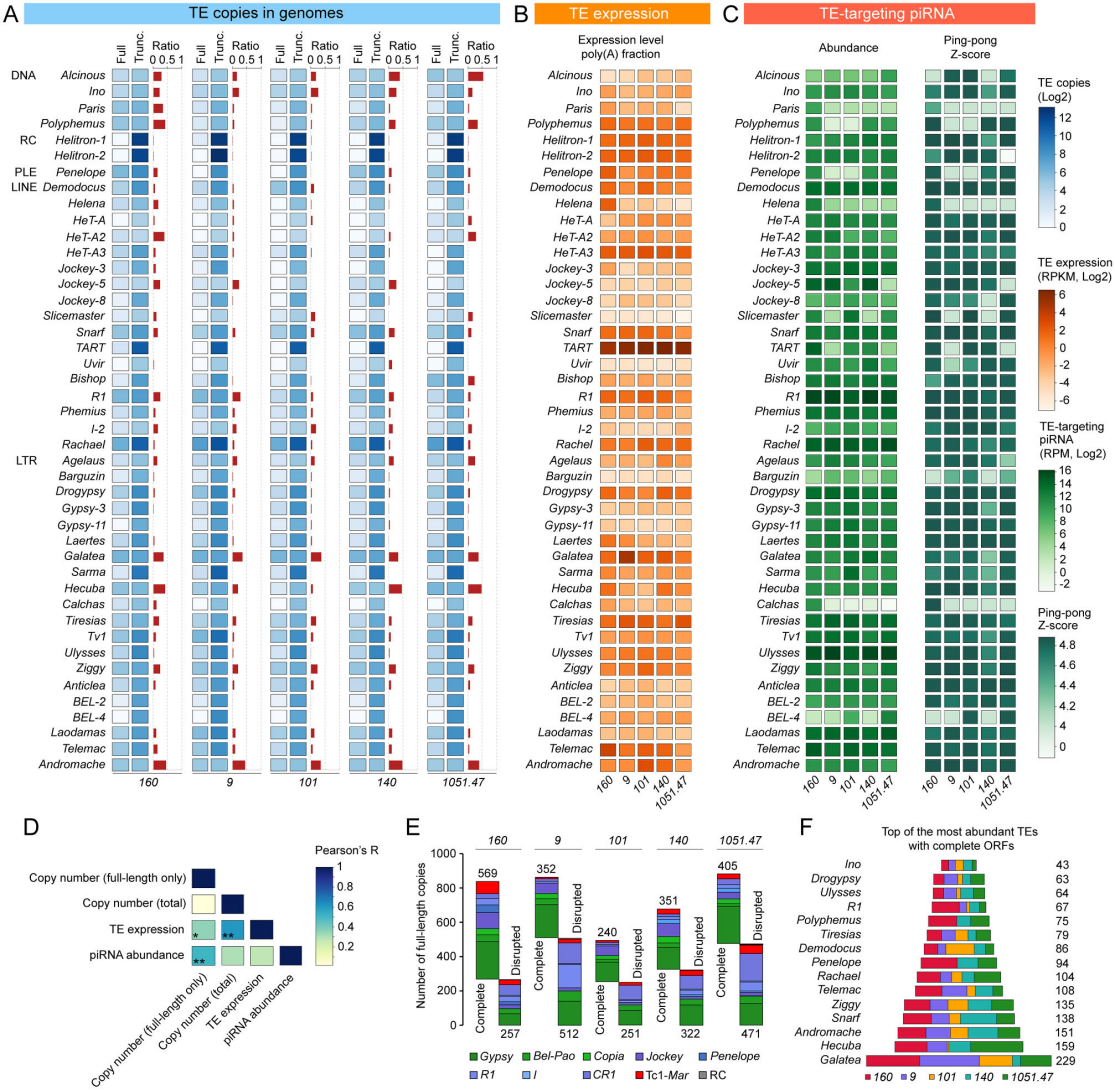
Comparative analysis of TE abundance, TE expression, and TE-targeting by piRNAs in *D. virilis* strains. (**A**) Heatmap depicting the number (log_2_ values) of full-length (≥90% of the canonical copy length) and truncated (<90% of the canonical copy length) TE copies in the genomes of strains studied. The ratio of full-length to truncated TE copies is given as bars on the right. Only intact TE families according to their canonical copies are shown. (**B**) Heatmap demonstrating the expression level (RPKM, log_2_) of intact TE families in ovaries of the studied strains. (**C**) Heatmaps showing the abundance of piRNAs (left) (RPM, log_2_) and ping-pong Z-scores (right) for each intact TE family in five strains. (**D**) Pearson’s correlation coefficient between copy number (full-length only and all (full-length + truncated) copies), TE expression, and piRNA abundance is shown as a heatmap. Pairwise correlations were calculated between all categories for each strain studied and presented as average values. Asterisks indicate *P*-value (*P <.05, **P <.01). (**E**) Number of full-length copies of intact TE families, divided according to the presence of complete or disrupted ORFs. An ORF was considered complete if it had at least 90% of the ORF length with preservation of the domain structure of the canonical element. Otherwise, an ORF was considered disrupted. Colours represent TE superfamilies. (**F**) The most abundant intact TE copies with complete ORFs in all genomes examined. The number of TE-copies on the right shows the sum of TE-copies in all strains.

We next analysed TE abundance across the five *D. virilis* strains to characterize common features and interstrain variation. As anticipated from the divergence landscape, the most prevalent TE families in the *D. virilis* genome are represented by the RC elements *Helitron-1* and *Helitron-2*, that had accumulated >4000 copies in each of the genomes studied (Fig. 4A and Supplementary Table S2). Along with *Helitrons*, DNA transposon *PaleoMar-1*, LINE elements *TART* and *Rachael*, as well as LTR retrotransposons of the *Boreas* and *Khamsin* families, were represented by more than a thousand copies in *D. virilis* genomes (Fig. 4A, Supplementary Fig. S8A, and Supplementary Table S2). However, according to their consensus sequences, only *Helitrons, TART*, and *Rachael* had intact full-length copies in the studied genomes (Fig. 4A). The other three TE families were represented only by truncated copies. Furthermore, despite a large number of copies of *Helitrons* and *TART*, their full-length representatives were found in only a few copies in the genomes of *D. virilis* (Fig. 4A and Supplementary Table S2).

Most potentially active TE family lineages consisted of fewer than 500 copies in the genomes, including both full-length and truncated forms (Supplementary Table S2). While many of these families were present in all five strains, we observed significant quantitative variation in the abundance of full-length copies for several elements, indicating differences in recent transpositional activity. For instance, the *Penelope* element and DNA transposon *Polyphemus* were present in a significantly larger number of full-length copies in the genomes of three of the five studied strains (*160, 140*, and *1051.47*) (Fig. 4A and Supplementary Table S2). Full-length copies of the LINE retroelement *Slicemaster* were found only in the genomes of strains *160, 101*, and *1051.47*. Another LINE element, *Jockey-5*, was present only in the genomes of strains *160, 9*, and *140* as a full-length copy (Fig. 4A and Supplementary Table S2). Certain TE families exhibited higher representation in only one strain in comparison to others. For example, full-length copies of the LINE element *Demodocus* predominated in strain *101*, while strain *1051.47* demonstrated a predominance of the *Bishop* element of the *R1* superfamily as well as the *Barguzin* LTR retroelement (Fig. 4A, Supplementary Fig. S9, and Supplementary Table S2).

In *D. virilis*, the *HeT-A* elements are divided into three subfamilies (*HeT-A, HeT-A2, HeT-A3*), and their composition differed across strains. Full-length copies of *HeT-A3* predominated in all studied strains except *1051.47*, ranging from 5 copies in the genome of strain *101* to 16 copies in the genome of strain *160* (Fig. 4A and Supplementary Table S2). The genome of strain *1051.47* did not contain full-length copies of *HeT-A3*, but comprised five copies each of *HeT-A* and *HeT-A2*, that are absent in the genomes of strains *101* and *140* (Fig. 4A and Supplementary Table S2).

Strain *160* was particularly distinctive, containing full-length copies of all known TE families and showing marked expansions of specific elements like the *Paris* DNA transposon, the *Helena* and *TART* LINE retroelements, and the *Calchas* LTR retrotransposon (Fig. 4A, Supplementary Fig. S9, and Supplementary Table S2). Importantly, the aforementioned interstrain variations are predominantly formed by intact TE families, whereas partial and remnant TE families exhibited a comparable number of copies across the *D. virilis* genomes studied (Supplementary Fig. S8A and Supplementary Table S2).

Profiling of TE expression in all studied strains using poly(A)-selected RNA-seq and analysis of TE-targeting piRNAs with perfect match (0 mismatches allowed) in ovarian tissue largely confirmed the identified common features and interstrain variations in the genomic abundance of intact TE families. Among the most expressed TEs in all strains in terms of TE expression and piRNA targeting, the following TE families can be distinguished: *TART, Helitron-1, Helitron-2, Galatea, Rachael, Tiresias, HeT-A3, Snarf, R1, Ulysses, Drogypsy, Telemac*, and *Andromache* (Fig. 4B and C). Strain-specific TE families (e.g. *Penelope, Helena*) also demonstrated higher levels of expression in the strains where they were most prevalent (Fig. 4B and C). Furthermore, the majority of identified intact TE families were targeted by piRNAs that exhibit a ping-pong signature, the hallmark of *bona fide* piRNA biogenesis (Fig. 4C).

We found a significant correlation between the number of TE copies (full-length + truncated) for intact TE families and TE expression (average Pearson’s R = 0.6, *P *<.01; Fig. 4D). The correlation was weaker when considering only full-length copies (R = 0.35, *P* <.05), suggesting that truncated copies also contribute to the transcript pool. Interestingly, while piRNA abundance correlated with the number of full-length copies (R = 0.5, *P* <.01), there was no significant correlation between TE expression and piRNA abundance, indicating a complex and nonlinear relationship between piRNA targeting and transcriptional output.

Conversely, despite the absence of substantial differences in the number of copies, partial and remnant TEs showed significant variation in expression and piRNA targeting between strains (Supplementary Fig. S8B and C). The correlation between TE copy number and expression also varied, with Pearson’s correlation coefficients of 0.4 in some strains and 0.6 in others (*P* <.05) (Supplementary Fig. S8D). As with intact TEs, their expression did not correlate significantly with piRNA targeting (Supplementary Fig. S8D).

This analysis revealed intraspecific variation in TE composition across *D. virilis* strains, with each exhibiting unique abundances of specific elements. These distinct TE profiles, supported by expression and piRNA data, indicate strain-specific potentials for TE mobilization that likely contribute to genomic diversity.

### The disruption of coding regions limits the functional capabilities of full-length TE insertions

Internal TE mutations can impede the translation of functional TE proteins, thereby limiting transposition even when the element is transcribed [94, 122, 123]. To assess functional potential, we analysed ORF integrity in full-length insertions, defining a ‘complete ORF’ as one covering ≥90% of the consensus sequence with preserved domain structure (see the ‘Materials and methods’ section for details).

The analysis demonstrated that approximately half of the full-length TE copies exhibited incomplete ORFs in four of the five genomes examined, due to mutations that disrupted their integrity (Fig. 4E and Supplementary Table S2). However, an exception to this generalization was observed in the genome of strain *160*, where the percentage of disrupted ORFs was lower (∼30%) (Fig. 4E and Supplementary Table S2). Among the TEs with the most copies containing complete ORFs, retrotransposon superfamilies were particularly predominant, including *Galatea, Hecuba, Andromache, Snarf, Ziggy, Telemac, Rachael, Penelope, Demodocus, Tiresias, R1, Ulysses*, and *Drogypsy* (Fig. 4F). Overall, ∼0.7% (in strains *101* and *9*) to 1.5% (in strain *160*) of all individual TE copies (full-length and truncated) were considered capable of transposition. It is noteworthy that the majority of these TEs were enriched in the first cluster, as determined by Kimura distance analysis (Fig. 2). In contrast, only *Rachael* and *Drogypsy* were enriched in cluster 2, and despite their age, both appeared to remain active in the genome (Fig. 2).

The number of complete and disrupted ORFs in strain *160* is likely genuine, as SNP analysis showed only minor differences in mutation counts between PacBio and ONT sequencing technologies (Supplementary Fig. S1B).

Our findings reveal that a significant proportion of full-length insertions were functionally inert. Consequently, a comprehensive assessment of TE activity must take into account ORF integrity analysis to accurately distinguish between potentially active elements and nonfunctional genomic relics, thereby refining our understanding of TE-driven genome evolution.

### Dynamic regulation of TEs by piRNA pathway in *D. virilis*

**Figure 5.**
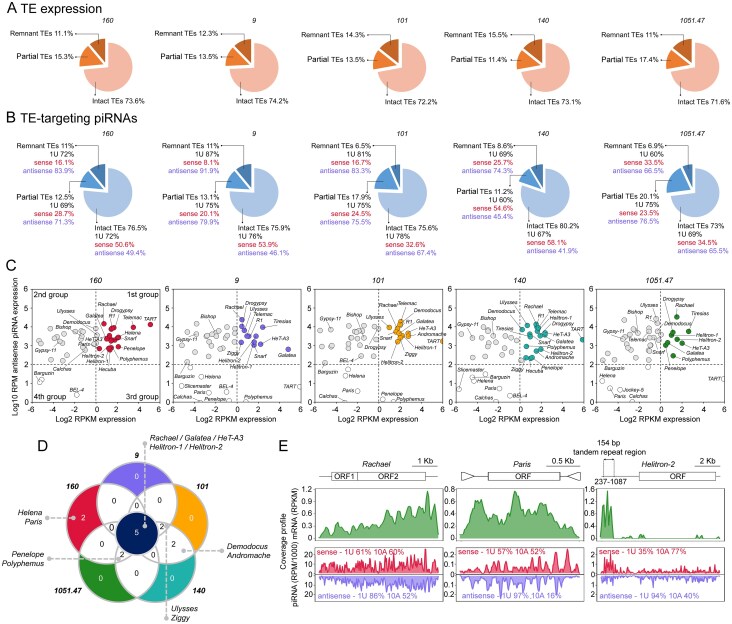
Dynamic regulation of TEs by piRNA pathway in *D. virilis*. (**A, B**) The ratio of intact, partial, and remnant TEs in terms of their expression and piRNA targeting in ovaries of the five *D. virilis* strains studied. Percentage of TEs according to the completeness of their canonical copies constituting the TEs expression pool (RNA-seq) and TE targeting piRNA population (small RNA-seq) in ovarian tissue, respectively. The 1U nucleotide bias, as well as the ratio of sense and antisense piRNAs (percentage), are shown. (**C**) Relationship between the expression level of TEs and the number of TE-targeting antisense piRNAs. The X-axes show the expression level of TEs determined by RNA-seq. The Y-axis demonstrates the expression level of antisense piRNAs. Expression values for RNA-seq and small RNA-seq were normalized to the number of reads per kilobase per million mapped reads (RPKM) and reads per million mapped (RPM), respectively. (**D**) The Venn diagram shows strain-specific and common TE families in the studied strains. Only the most expressed TEs, according to TE expression analysis and piRNA targeting, were considered. (**E**) Examples of mapping of RNA-seq and piRNA reads on the LINE retroelement of *CR1* superfamily, *Rachael*, DNA transposon of *Tc1-Mariner* superfamily, *Paris*, and *Helitron-2*. Data from strains *1051.47, 160*, and *9* were used to plot RNA-seq and piRNA reads for *Rachael, Paris*, and *Helitron-2*, respectively. Nucleotide biases, including 1U and 10A (percentage), are shown for both sense and antisense piRNA mappers (23–29 nt). The 154 bp tandem repeat region corresponding to the 237–1087 nt region of *Helitron-2* is shown.

The expression of TEs is tightly regulated by the piRNA pathway, which targets active TEs through sequence complementarity, preventing deleterious mutations and insertions in the germline and somatic cells [17, 18]. The proportion of intact, partial, and remnant TEs in the TE transcript pool closely mirrored the distribution of piRNAs targeting these elements. Intact TE families accounted for >70% of the TE transcript pool in ovaries and are the primary target of piRNA regulation in all strains of *D. virilis* (Fig. 5A). The remaining piRNAs were distributed among partial and remnant TEs, with variation observed between the strains studied (Fig. 5B).

As revealed by correlation analysis, a clear association between TE expression and piRNA abundance was not always observable (Fig. 4D). We sought to analyse this relationship more thoroughly in every studied strain. We categorized the intact TE families into four distinct groups based on their expression levels and the abundance of TE-targeting piRNAs. The first group comprised TEs that demonstrated both TE expression (log_2_ RPKM > 0) and antisense piRNAs targeting these elements (log_10_ RPM ≥ 2) (Fig. 5C). This group likely represents the active TE families in *D. virilis* strains, as supported by genome-wide analysis of full-length TE copy number. The second group consisted of TEs that exhibited low levels of TE expression (log_2_ RPKM < 0) but high levels of antisense piRNAs (log_10_ RPM > 2; Fig. 5C). The discordance in TE expression and their piRNA targeting between the aforementioned groups likely explains the observed lack of significant correlation described previously (Fig. 4D). Importantly, according to Kimura distance, the first group of TEs, with the exception of *Rachael* and *Drogypsy*, consisted of young families (cluster 1; Fig. 2C). In contrast, the second group was primarily composed of older families (cluster 2; Fig. 2C), indicating that these TE families have been effectively suppressed by the piRNA pathway throughout their extended presence in the genome. The third group comprised TEs that are expressed in ovarian tissue (log_2_ RPKM > 0) but demonstrated low targeting by antisense piRNAs (log_10_ RPM < 2; Fig. 5C). TEs in this group were highly strain-specific, indicating that the same TE families may be active in one strain while represented only by inactive relics of past invasions in others. Consequently, these strains lacked piRNAs that perfectly matched (0 mismatches) and targeted these elements. Finally, the fourth group represented TEs that exhibited a negligible level of both TE expression (log_2_ < 0) and antisense piRNAs (log_10_ < 2) in the particular strain (Fig. 5C). Collectively, these findings demonstrate that the piRNA pathway dynamically suppresses TE expression to maintain a balance between genome plasticity and stability.

Among the actively expressed TEs (group 1), we identified both universal and strain-specific families (Fig. 5D). *Rachael, Galatea, HeT-A3, Helitron-1*, and *Helitron-2* were highly expressed across all strains. In contrast, *Penelope* and *Polyphemus* expression was elevated in strains *160, 140*, and *1051.47*, while *Paris* and *Helena* were distinctive markers of strain *160*.

In summary, our analysis revealed a dynamic and nuanced interplay between TEs and the piRNA pathway in *D. virilis*. The piRNA system does not uniformly silence all TEs but rather establishes distinct regulatory relationships, categorizing TE families into groups based on their expression and piRNA targeting. This results in effective suppression of older elements, active engagement with recent invaders, and strain-specific patterns that reflect the unique TE history and piRNA repertoire of each population, thereby maintaining a crucial balance between genomic stability and plasticity.

### Expression profiling of TEs suggests piRNA biogenesis from TE-derived tandem repeats in *D. virilis*

Despite extensive methodological developments for measuring TE expression using RNA-seq data, the efficacy of these approaches is constrained by the inherent characteristics of TEs [124]. Since TEs are highly repetitive and evolutionarily related TE families often coexist in the same genome, short sequencing reads derived from TEs frequently map equally well to multiple genomic locations.

With this in mind, we analysed the coverage of RNA-seq and piRNA reads across the sequences of intact 44 TE families (Fig. 1). Examples of the most common TE families, *Rachael* and *Helitron-2*, as well as *Paris*, that illustrate interstrain variations, are shown in Fig. 5E. The coverage profile for *Paris* showed a relatively uniform distribution of RNA-seq reads, while for *Rachael*, the mapping profile was shifted towards the 3′-end of the TE sequence (Fig. 5E). This is typical for LINE retroelements, that are frequently represented by 5′-truncated copies (Fig. 5E) [125, 126]. An intriguing coverage pattern was observed for *Helitron-2*, which demonstrated a marked preference for RNA-seq mapping to the 5′ end of its sequence. Previously, we showed that 154 bp tandem repeats represent one of the most abundant families of medium-sized repeats in the *D. virilis* genome [127]. This repeat demonstrates a high degree of homology to the region between positions 237 and 1087 in *Helitron-2*, suggesting that the 154 bp tandem repeats originated from this transposon. The analysis performed in this study demonstrated that the 237–1087 *Helitron-2* region exhibited elevated levels of expression for both RNA-seq reads and TE-targeting piRNAs (Fig. 5E). This finding indicates that the 154 nt tandem repeat is transcribed and functions as a source for piRNA production. Importantly, the expression of the full-length copy of *Helitron-2* was also determined by northern blot hybridization [127]. Similarly, the coverage profile for *Helitron-1* also demonstrated a high prevalence of RNA-seq reads mapping to the 5′-end of the TE sequence (Supplementary Fig. S10). Given that the *D. virilis* genome contains even more copies of *Helitron-1* than *Helitron-2*, but retains only a few full-length copies (Supplementary Table S2), one may suggest that another medium-sized tandem repeat may be derived from *Helitron-1*, but this has yet to be described.

### Evidence for HT events in *D. virilis* species group

In *Drosophila melanogaster*, studies have shown that close relatives frequently share recently mobilized TE lineages through HT [128, 129].

**Figure 6.**
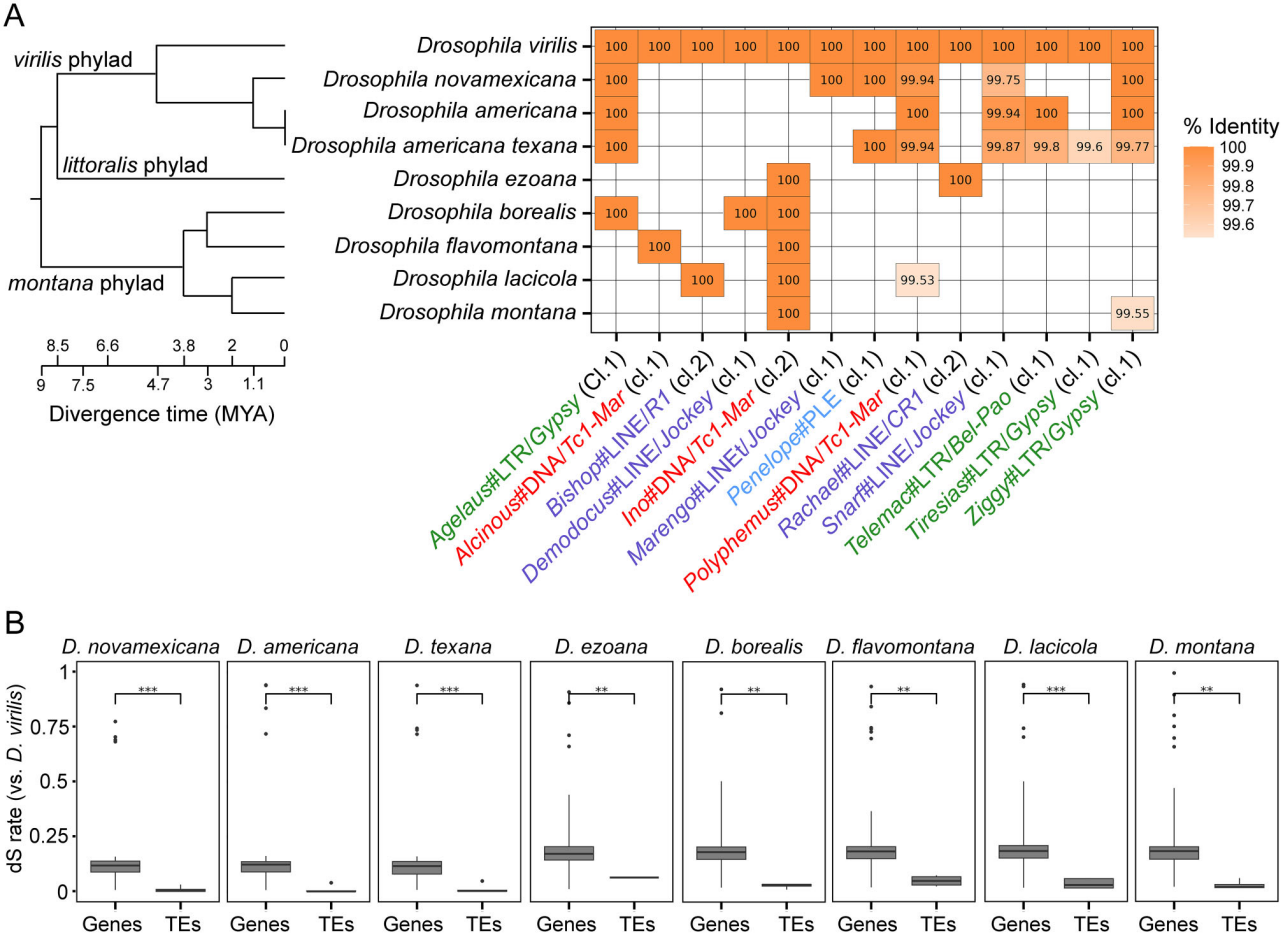
HT of TEs among the *D. virilis* species group. (**А**) The results demonstrate the presence of *D. virilis* TEs in related species, with a minimum completeness of 90% and sequence identity of at least 99.5% compared to the consensus *D. virilis* TE copies. The cluster’s numbers are given according to the Kimura distance analysis. The species phylogenetic tree used in this analysis was sourced from Yusuf *et al.* [56]. (**B**) The synonymous substitution rate (dS) for single-copy orthologous genes and TE ORFs between *D. virilis* and other species in the group was determined. A conserved set of 50 single-copy orthologs (genes) and 13 potentially horizontally transferred TEs was used. Statistical support was determined by the Mann–Whitney U-test, followed by FDR correction. **P <.01; ***P <.001.

We thus sought to determine the degree to which elements active in the *D. virilis* genome were also active in relatives. If such TE families showed residence within relatives of *D. virilis* and also exhibited near identity at the sequence level, this would suggest broad sharing via HT among species within the *virilis* group. We evaluated this using blastn to identify, among the described 100 TE families, those also present in available genome sequences of eight species within the *virilis, littoralis*, and *montana* phylads. Among these TEs, we considered families with identity >99.5% in another species (all species have >4 million years of divergence from *D. virilis*) to be most likely appeared by HT. This approach revealed 13 families with at least 99.5% identity in at least one another species (Fig. 6A). Eleven of these 13 families were found in another species with 100% identity. Of these 13, three were DNA transposons and ten were retrotransposons. Most sharing of identical TE lineages was among the *D. virilis* phylad (*D. virilis, Drosophila novamexicana, Drosophila americana, Drosophila americana texana*), but five TE families had 100% identical copies in at least one species of the *montana* phylad, which diverged from *D. virilis* about 9 million years ago (Fig. 6A). In fact, one DNA transposon (*Ino*) was present in all studied species of the *montana* phylad, but was found only in *D. virilis* among the species of the *virilis* phylad (Fig. 6A). We also calculated the synonymous substitution rate (dS) for the top 50 orthologous genes between *D. virilis* and other species in the group. We confirmed that for each of the 13 families proposed to have been horizontally transferred, the TE sequence identity was significantly higher (*P* <.001) than the background dS value (which had median values ranging from 12 to 20), as shown in Fig. 6B.

This indicates that HT is frequent among even quite divergent species of the *virilis* group.

### Distinct TE insertional landscapes drive genomic variations in *D. virilis*

To understand how TE landscapes differ across *D. virilis* strains, we applied TELR [107]. The application of TELR revealed hundreds of nonreference TE insertions, ranging from 584 in strain *1051.47* to 323 in strain *9* (Supplementary Fig. S11A). The differences in the number of identified nonreference TE insertions are not likely attributable to variation in coverage and read length between the strains, as evidenced by an analysis of read length and coverage-normalized datasets (Supplementary Fig. S11B). However, a slight underestimation of insertions may have occurred in strain *160* due to the limited amount of sequencing data available (genome coverage ∼30×).

**Figure 7.**
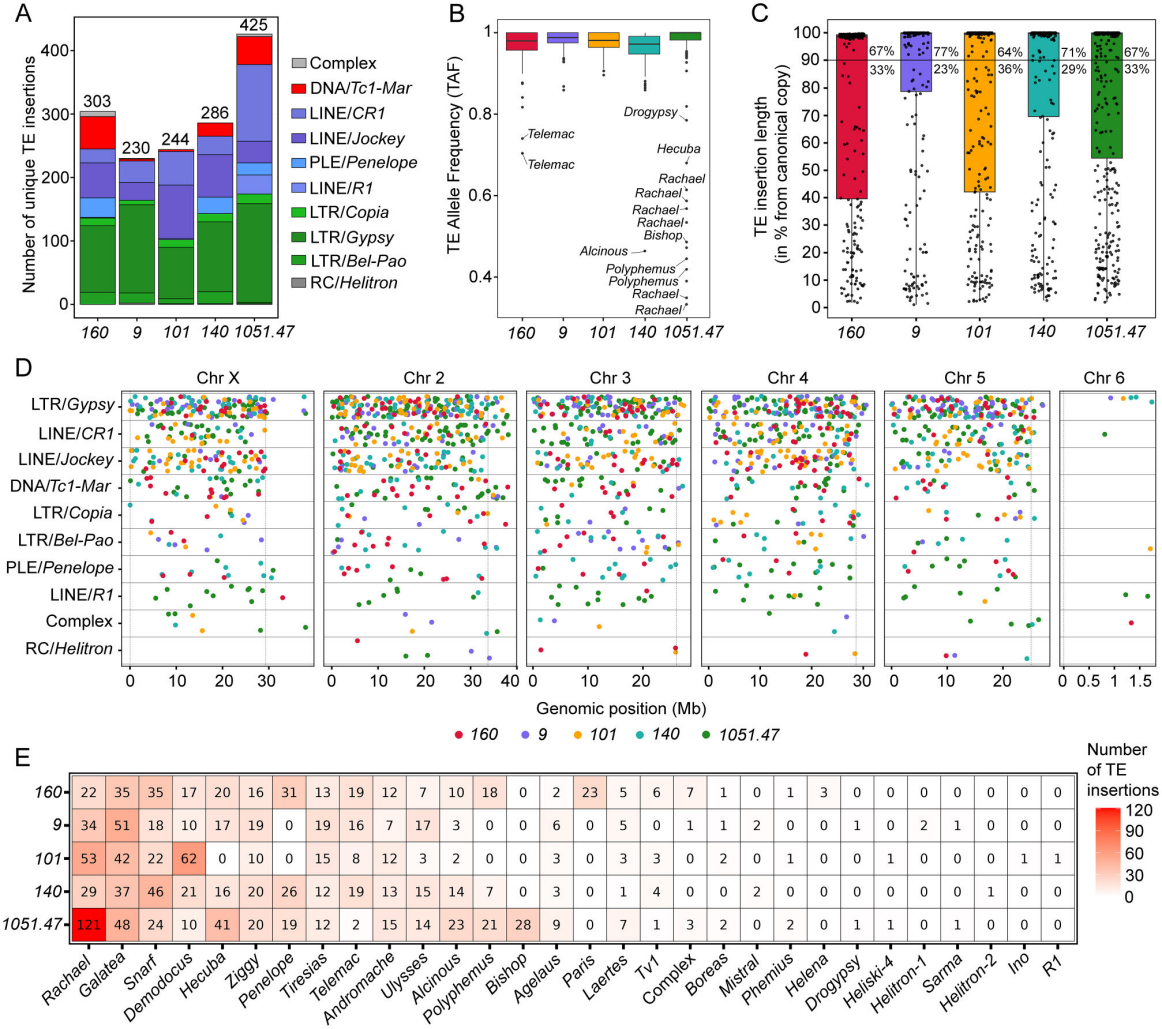
Abundance, allele frequency, and genome-wide distribution of unique TE insertions in *D. virilis* strains. (**A**) Number of unique TE insertions for *D. virilis* strains *160, 9, 101, 140*, and *1051.47* partitioned by TE superfamilies. (**B**) Genome-wide TE allele frequency (TAF) distribution for studied strains. (**C**) Length of unique TE insertions. The length is given as a percentage relative to the length of the canonical TE sequence. The percentage of full-length and truncated/partial TE insertions is indicated next to the boxes. TE insertions that are at least 90% of the length of the canonical element are considered full-length TE insertions. (**D**) Genome-wide distribution of unique full-length TE insertions across chromosomes is indicated for each strain. Dotted vertical lines indicate pericentromeric regions. (**E**) The heatmap shows the number of unique TE insertions in each genome, divided by TE family.

Further to estimate the number of TE insertions that comprise the unique landscape of TE invasions, we removed those nonreference TE insertions that overlap between genomes 5 kb upstream and downstream from the TE insertion coordinates defined for each strain. Applying this approach, we identified a total of 425 unique nonreference TE insertions in strain *1051.47*, 303 in strain *160*, 286 in strain *140*, 244 in strain *101*, and 230 in strain *9* (Fig. 7A). On the average, 70% of the identified nonreference TE insertions were unique to a single genome (Supplementary Fig. S11A). Analysis of TE age (Kimura distance) showed that 80% of these unique insertions were young, while <1% were ancient (Supplementary Fig. S11C). In contrast, nonunique insertions had a more balanced age distribution. Elements from the intermediate-age *Rachael* family made up about 20% of both unique and nonunique groups (Supplementary Fig. S11C).

Most unique insertions were homozygous (TE allele frequency, TAF ∼1) and full-length (∼70%; Fig. 7B and C), indicating their recent fixation. However, a notable exception was observed in strain *1051.47*, where a group of insertions from various TE families, including *Rachael, Bishop, Hecuba*, and *Polyphemus*, were found to be heterozygous (TAF < 0.7; Fig. 7B). While predominantly euchromatic, some full-length insertions from *Gypsy, CR1*, and *Tc1-Mariner* superfamilies were located in heterochromatin (Fig. 7D).

We identified 29 TE families contributing to this strain-specific variation, 26 of which were intact (Figs 7E and 1). This analysis confirmed the uneven distribution of specific families, such as the exclusive activity of the *Paris* DNA transposon in strain *160* and the unique *Bishop* insertions in strain *1051.47*. Transpositions of *Penelope* and *Polyphemus* occurred in strains *160, 140*, and *1051.47* but not in *101* or *9*. Four families were particularly prolific, with over 100 insertions across all genomes: the LINE elements *Rachael, Snarf, Demodocus*, and the LTR element *Galatea*. Notably, *Rachael* underwent a major expansion in strain *1051.47*, with more than twice as many insertions as in other strains studied (Fig. 7E).

Our analysis revealed distinct TE landscapes across *D. virilis* strains. This variation was not random. Different strains exhibited unique “signature” expansions of specific TE families, such as *Paris* in strain *160* and *Rachael* in strain *1051.47*. Collectively, the identification of hundreds of strain-specific, unique TE insertions, predominantly from young, intact families, demonstrates that recent and ongoing TE activity has actively shaped the genomes of different populations of *D. virilis*.

### TE insertions modify gene expression in a distance- and location-dependent manner

Transposon insertions can significantly influence gene expression through various genetic and epigenetic mechanisms, ranging from direct disruption of coding sequences (CDS) to epigenetic silencing and long-range chromatin effects [1, 32].

**Figure 8.**
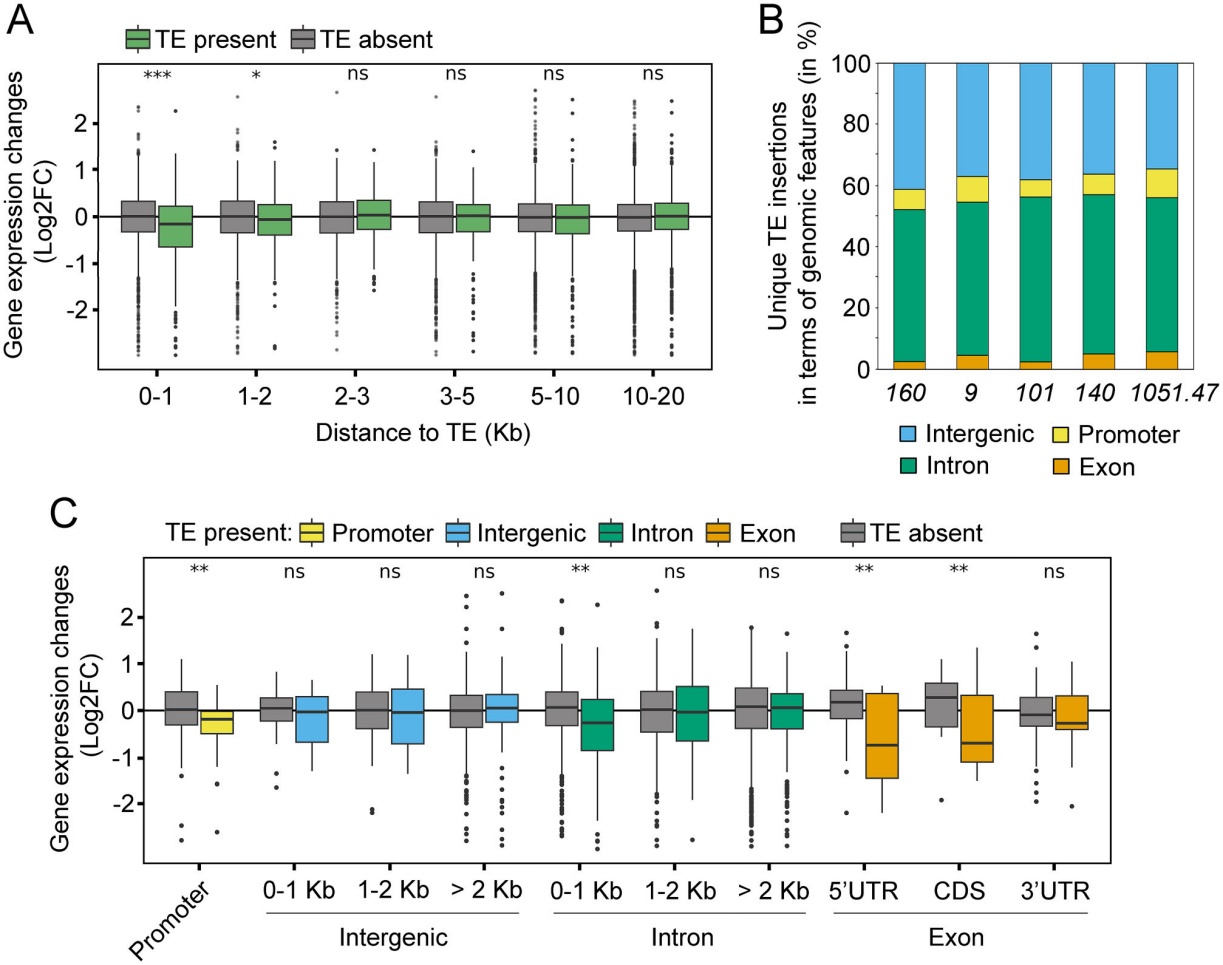
Effects of TE insertions on host gene expression. (**A**) The pattern of gene expression changes according to the distance to the TE insertion. (**B**) The distribution of unique TE insertions across genomic features is given for strains *160, 9, 101, 140*, and *1051.47*. A region up to 1 kb upstream of the transcription start site of genes is considered the promoter region. (**C**) The pattern of gene expression changes depending on the genomic features. Statistical significance in was assessed by the Mann–Whitney U test followed by FDR correction. * indicates *P* <.05, ** indicates *P *<.01 and *** denotes *P* <.001.

To investigate the influence of TEs on the expression of adjacent genes, we categorise protein-coding genes based on their distance to the unique TE insertions identified previously (0–1 kb, 1–2 kb, etc.). From this point onwards, the “TE present” category denotes the strain containing a unique TE insertion, whereas the “TE absent” category refers to the control group comprizing all other strains lacking that specific insertion (see the ‘Materials and methods’ section for details). We analysed ovarian mRNA expression for each of the five strains individually, and then combined the results to provide a general overview. We found that TEs affected the expression of genes located up to 2 kb from their insertions (0–1 kb, *P* <.001; 1–2 kb, *P* <.05; Mann–Whitney U test followed by FDR correction; Fig. 8A). No significant changes in gene expression were observed in the region >2 kb from the TEs (Fig. 8A). Both correlation and principal component analyses demonstrated a high level of similarity between the biological replicates for each sample (Supplementary Fig. S12).

Approximately 45% of all unique TE insertions were located within gene introns (Fig. 8B). A substantial proportion of TEs, reaching about 40%, were located within intergenic regions. Only 5%–8% of TE insertions were found in gene promoters, and ∼5% were located in gene exons (Fig. 8B). This distribution was only slightly different between full-length and truncated TE insertions (Supplementary Fig. S13A).

A general decrease in gene expression was observed when TE insertions were located in promoters and introns (Fig. 8C). However, for intronic insertions, this decrease was only observed if the TE was positioned within 1 kb of the nearest exon (*P* <.01, Mann–Whitney U test, FDR-corrected) (Fig. 8C). As expected, a marked decline in gene expression was evident due to TE insertions within gene exons (Fig. 8C). A statistically significant decrease in gene expression was also detected for TE insertions in the 5′ untranslated region (5′ UTR) and CDS, but not in the 3′ untranslated region (3′ UTR; Fig. 8C).

The location of TE insertions relative to genes had a significant impact on gene expression. Overall, 187 of 521 genes (35.8%) with nearby TE insertions showed significant differential expression (*p*-adj < 0.05) (Supplementary Table S3). Of these, 127 genes were downregulated (log_2_FC range: −0.16 to −8.7) and 60 were upregulated (log_2_FC range: 0.34–2.27). A breakdown by TE location showed that intronic insertions had the broadest effect, significantly altering expression for 138 of 400 genes. Promoter and exonic insertions affected 19 of 49, and 17 of 29, respectively (Supplementary Table S3).

Herein, we systematically assessed how TE insertions may influence nearby gene expression using a comparative genomics approach, integrating high-confidence TE insertion calls with gene expression data. Our analysis demonstrated that TE insertions can act as local modulators of gene expression in the *D. virilis* genome and are primarily associated with gene repression. The magnitude of this repression is determined by a dual dependency: distance from a gene (maximal within 0–1 kb, detectable up to 2 kb) and genomic location (most severe in promoters, exons, and introns located close to exons).

### Heterochromatin spreading from TE insertion sites contributes to a reduced expression of adjacent genes

To provide a mechanistic interpretation of the observed overall reduction in gene expression near TE insertions, we examined the epigenetic effects of TE silencing by profiling the repressive histone mark H3K9me3 in the ovaries of all five strains studied. Specifically, we performed ChIP-seq for strains *101* and *1051.47* as well as re-analysed ChIP-seq data for strains *160, 9*, and *140* that had been published previously [72, 97]. We then quantified H3K9me3 enrichment near unique TE insertions by comparing strains where a specific TE was present to strains where it was absent (see the ‘Materials and methods’ section for details). This analysis was performed separately for full-length and truncated TE insertions.

**Figure 9.**
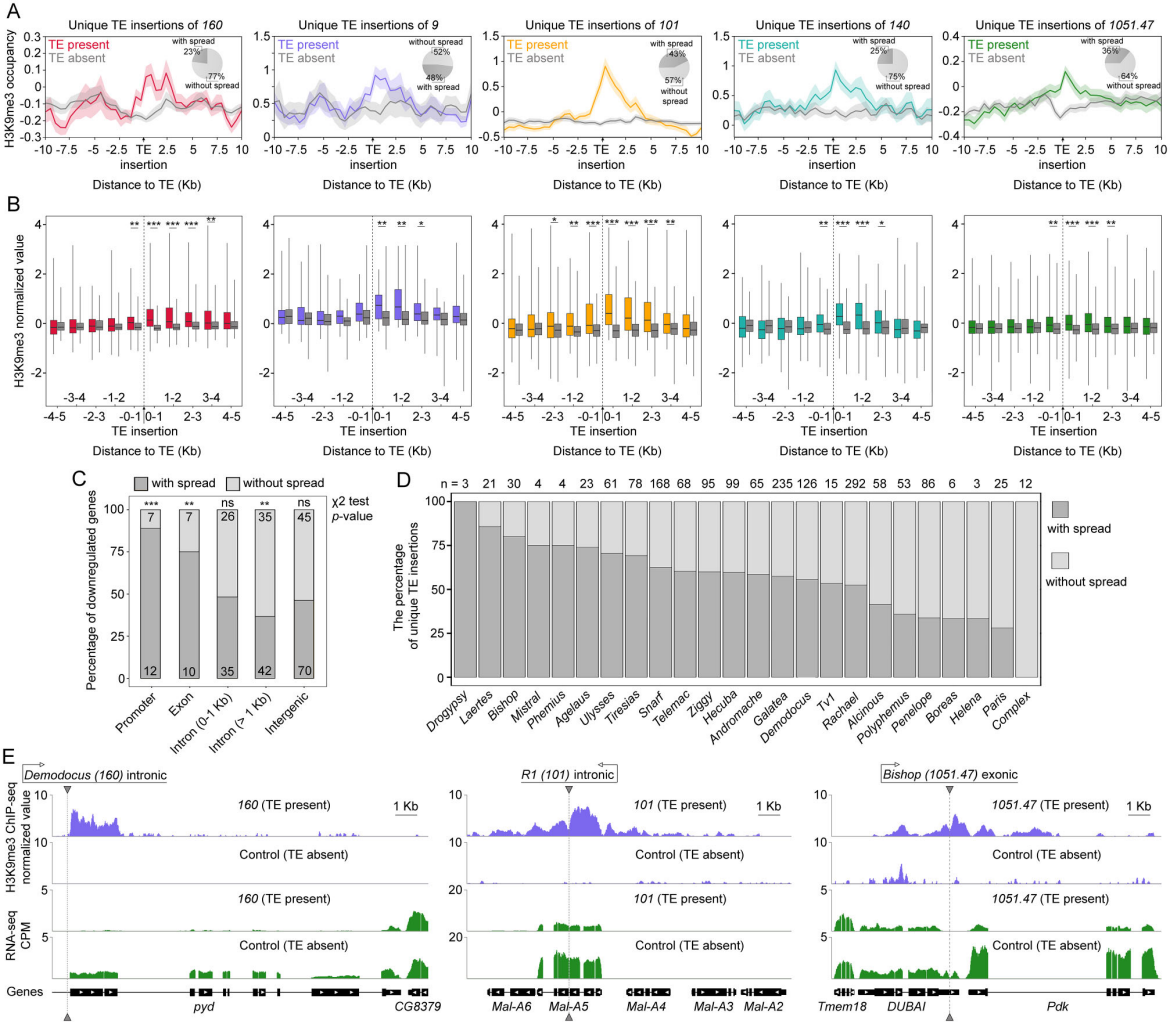
Epigenetic effects of TE insertions on host gene expression. (**A**) The genome-wide H3K9me3 occupancy around full-length TE insertions in the studied genomes. The shaded areas indicate the 95% confidence interval. The pie chart in the corner on the right shows the percentage of TE insertions with or without determined H3K9me3 spread. (**B**) The enrichment analysis of H3K9me3 occupancy in 0–1 kb windows flanking TE insertions. The colours for the experimental group (TE present) and the control group (TE absent) correspond to those shown in panel (D). (**C**) The percentage of TE insertions with and without H3K9me3 spread according to the genomic features. The numbers on the bars indicate the number of TE insertions with and without H3K9me3 spreading in each category. ** and *** denote *P*-values <.01 and <.001, respectively, determined by chi-squared test. (**D**) The proportion of TE insertions with epigenetic effects for each TE family. All unique TE insertions observed for all five strains were used. The value (n) indicates the number of unique TE insertions for a given TE family. (**E**) Examples of H3K9me3 spreading affecting gene expression at the *pyd, Maltase*, and *Pdk* gene loci. Gene names are given according to the names of their orthologues in *D. melanogaster*. Statistical significance in (B), (C), and (E) was assessed by the Mann–Whitney U test followed by FDR correction. * indicates *P* <.05, ** indicates *P *<.01 and *** denotes *P* <.001.

As indicated by the H3K9me3 profiles, heterochromatin spreading was observed at distances of up to 4 kb from full-length TE insertions (Fig. 9A and B). Moreover, H3K9me3 spreading of up to 4 kb was only evident in two of the five analysed strains, specifically in strains *160* and *101*. In the remaining three strains (*9, 140*, and *1051.47*), a statistically significant enrichment of H3K9me3 was observed in the region extending up to 3 kb from TE insertions (Fig. 9A and B). The level of the H3K9me3 mark also appeared to be higher in the downstream region compared to the upstream region of TE insertions across all strains examined (Fig. 9A and B). Importantly, only a minority of unique TE insertions (23%–48%) demonstrated H3K9me3 spreading across all *D. virilis* strains examined (Fig. 9A and B).

The enrichment of H3K9me3 in the regions flanking TE insertions was significantly more pronounced for full-length insertions compared to truncated ones (Supplementary Fig. S13B). In contrast to full-length TE insertions (Fig. 9A and B), we observed the spread of H3K9me3 exclusively in strain *101*, and only up to 1 kb downstream of truncated TE insertions (Supplementary Fig. S13C and D).

In Drosophila, both di- and trimethylation of H3K9 have been demonstrated to function as repressive post-translational histone modifications [130, 131]. To investigate their roles, we performed ChIP-seq on strain *140* ovaries and found no statistically significant difference in H3K9me2 and H3K9me3 enrichment around TE insertions, indicating that the epigenetic effects of TE silencing involve both examined H3K9 modifications in similar proportions (Supplementary Fig. S14).

Crucially, the functional impact of this spreading was highly dependent on genomic context. Association analysis revealed that H3K9me3 spreading strongly correlated with gene repression when TEs were located in promoters or exons (χ² test, *P* <.01; Fig. 9C). In contrast, for intronic insertions located >1 kb from an exon, reduced gene expression was typically not associated with H3K9me3 spreading (χ^2^ test, *P* >.05). Furthermore, for TE insertions located >1 kb from the nearest exon, a reduction in gene expression was less likely to be associated with H3K9me3 spreading (χ^2^ test, *P* <.01; Fig. 9C).

The propensity to induce heterochromatin also varied among TE families. For instance, insertions of the LTR retrotransposon *Drogypsy* showed the strongest H3K9me3 enrichment and the highest proportion of insertions with epigenetic effects (100%), though they were rare (Fig. 9D and Supplementary Fig. S15). In contrast, more abundant families like *Rachael* and *Galatea* exhibited moderate enrichment, with only about half of their insertions causing detectable spreading (Fig. 9D and Supplementary Fig. S15).

As a case in point, consider the insertions of the LINE retroelements *Demodocus, R1*, and *Bishop* in the genomes of *160, 101*, and *1051.47*, respectively (Fig. 9E). In all cases, the TE insertions induced spreading of the heterochromatin mark H3K9me3. The insertion of *Demodocus* was associated with a substantial decrease in the expression of the *pyd (polychaetoid)* gene (log_2_FC = −2.6, *p*-adj < 0.05) in strain *160* (Fig. 9E). Similarly, the insertion of *R1* into the maltase A gene cluster led to a drastic decrease in *Mal-A5* gene expression (log_2_FC = −2.2, *p*-adj < 0.05) in the genome of strain *101* (Fig. 9E) [132]. Interestingly, *Mal-A5* is the only gene from the maltase A cluster that is expressed in the ovaries of *D. melanogaster* according to modENCODE [133]. Given the conservation of the maltase gene clusters in Drosophila species, it can be assumed that oogenesis in strain *101* is accompanied by glucose deficiency. Finally, the insertion of *Bishop* in the genome of *1051.47* is of particular interest, as it is a potential factor for the concurrent downregulation of the expression of two genes concurrently. This TE insertion occurred in the exon encoding the 3′UTR of the *DUBAI* (*Deubiquitinating apoptotic inhibitor*) gene and was associated with H3K9me3 spreading both upstream and downstream. This was associated with the downregulation of both the *DUBAI* (log_2_FC = −1, *p*-adj < 0.05) and *Pdk* (*Pyruvate dehydrogenase kinase*) (log_2_FC = −2.8, *p*-adj < 0.05) genes strain *1051.47* (Fig. 9E).

Our findings demonstrate that heterochromatin spreading from TE insertion sites is a significant mechanism contributing to the reduced expression of adjacent genes in *D. virilis*. This epigenetic effect is most pronounced for full-length TE insertions, with the repressive H3K9me3 mark extending up to 4 kb and exhibiting a directional bias, primarily downstream. Crucially, the functional impact of this spreading is highly dependent on genomic context: it is strongly associated with gene repression when TEs are located in promoters or exons, but has a negligible effect for intronic insertions distant from exons. This suggests that the epigenetic consequences of TE silencing are not uniform, but are modulated by both the structural integrity of the TE and its precise location within the genome.

### Retrotransposon insertions are associated with a polymorphic inversion in *D. virilis*

It is widely established that TEs are frequently linked to chromosomal rearrangements across various species, including Drosophila, with inversions being a predominant occurrence [3, 134].

**Figure 10.**
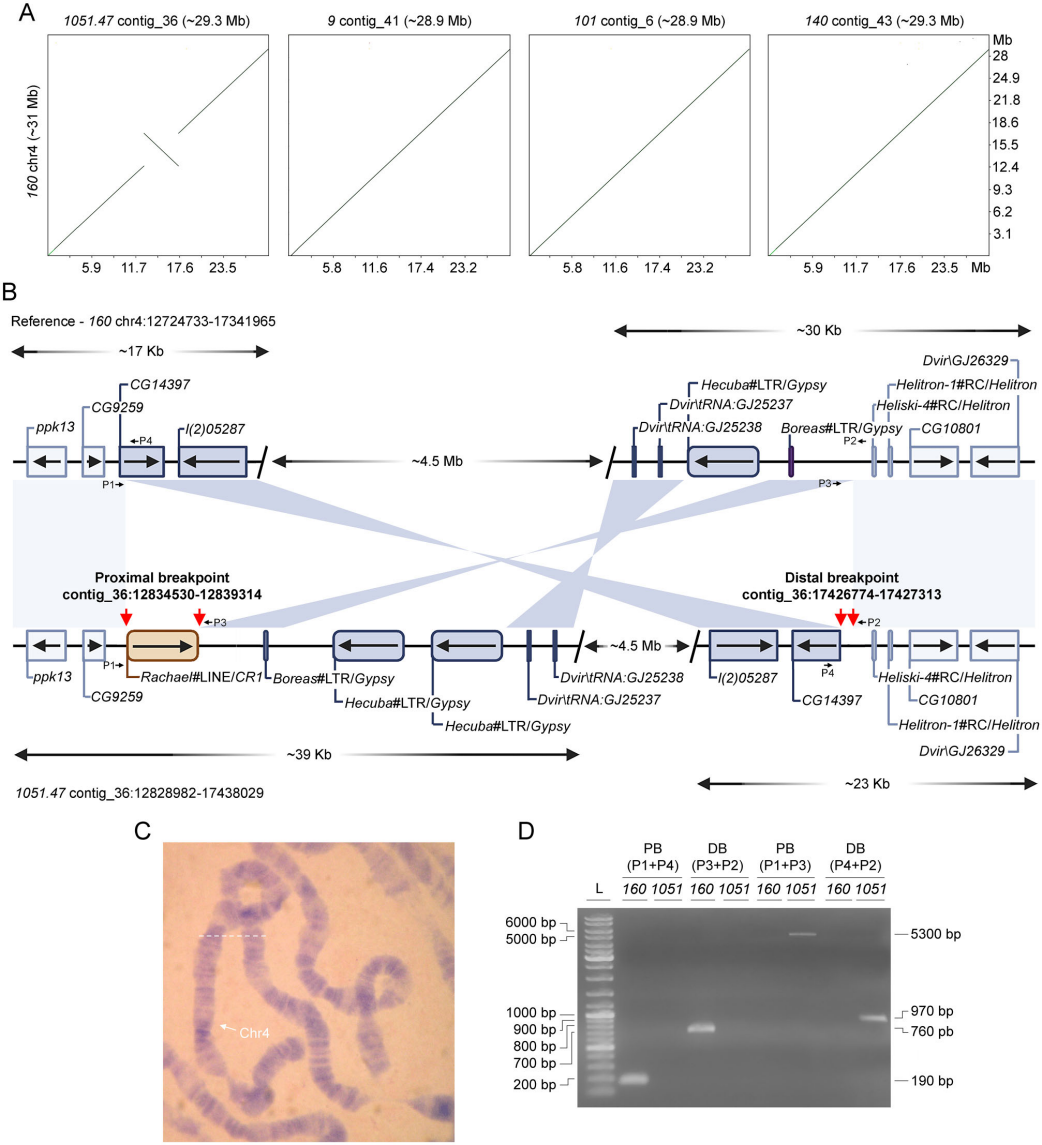
Polymorphic chromosomal inversion in *D. virilis*. (**A**) The dot plots illustrate the inversion in the genome of strain *1051.47* in comparison to the chromosomal assembly of the genome of strain *160* and other *D. virilis* strains that have been analysed. Up to 10% divergence was allowed for sequence alignment. (**B**) Schematic representation of the inversion region in the genomes of strains *1051.47* and *160*. Light and dark grey indicate regions of homology beyond and within the inversion breakpoints, respectively. The names of genes are given according to the names of their orthologues in *D. melanogaster*, if the gene ortholog exists. (**C**) Polytene chromosomes of the salivary glands of the F1 hybrid from the cross between the strains *1051.47* and *9*. The dotted white line indicates the inversion region at cytogenetic loci 44B–44E of chromosome 4 (Muller B). (**D**) PCR validation of the inversion breakpoints. The alignment of the P1–P4 primer regions is shown in (B). L is a ladder. Strain *1051.47* is referred as *1051*.

In the course of our study, we identified a spontaneous inversion of ∼4.5 Mb in length located in the medial part of the fourth chromosome (Muller B) in *D. virilis* strain *1051.47* (Fig. 10A). Precise mapping of the breakpoints demonstrated that the proximal inversion breakpoint was located in a region downstream of the *CG9259* gene locus in strain *1051.47* (contig_36:12834530-12839314) (Fig. 10B). The distal inversion breakpoint was located in the region between the *CG14397* and *CG10801* gene loci in the genome of strain *1051.47* (contig_36:17426774-17427313) (Fig. 10B).

The use of the blastn algorithm for pairwise alignment of the corresponding genomic regions encompassing the proximal inversion breakpoint revealed a 4784 nt gap in the genome of strain *1051.47*. Further analysis indicated a full-length *Rachael* TE insertion located just between the aligned homologous regions at the proximal breakpoint (Fig. 10B). Furthermore, the genomic region downstream of the *Rachael* insertion also showed a loss of homology between the compared genomes. This loss can be attributed to an imbalance in the number of insertions of another TE, the LTR retrotransposon *Hecuba*. Specifically, a single *Hecuba* insertion was identified in strain *160*, while double *Hecuba* insertions were detected in genome *1051.47* (Fig. 10B).

The *DAIBAM* Miniature Inverted-repeat Transposable Elements (MITE) is implicated in at least 20% of both intraspecific and interspecific chromosomal inversions observed in the *virilis* subgroup [135]. Furthermore, *DAIBAM* is implicated in 80% of inversions in *Drosophila americana* [136]. Following this, we checked for the presence of *DAIBAM* at both inversion breakpoints in the *1051.47* genome. We did not find *DAIBAM* sequences at or near the identified breakpoints, indicating that *DAIBAM* is not associated with the inversion breakpoint in this case.

To confirm the detected chromosomal inversion, strain *1051.47* was crossed with strain *9*. In the salivary gland chromosomes of the F1 hybrid, the inversion appeared as a loop at cytological loci 44B–44E (Fig. 10C). The breakpoints were validated by PCR (Fig. 10D).

## Discussion

### A high-quality curated library revealed historical waves of TE colonization in the *D. virilis* genome

High-quality TE annotation is fundamental for understanding genome function, evolution, and stability, as well as TE-host interactions [87, 88]. Historically, only a limited number of TEs have been annotated and curated for *D. virilis*. This TE annotation relied mostly on direct analysis of TE insertions that were identified as the causal factor for phenotypic mutations during the study of hybrid dysgenesis [58, 137–143, 144].

By integrating computational predictions from RepeatModeler2 with existing databases and rigorous manual curation, we have established a refined library of 100 TE families for *D. virilis*. This resource, which includes 44 intact, 26 partial, and 30 remnant elements, provides a robust foundation for the current and future studies, correcting and expanding upon previous TE annotations.

A notable feature of many DNA transposons is the generation of short yet transpositionally active elements known as MITEs (miniature inverted-repeat TEs) [145]. Typically, MITEs lack coding capacity but possess conserved TIRs and frequently attain high copy numbers in genomes. The *D. virilis* genome has been shown to harbour abundant MITEs, with several families already documented [74, 135, 136, 146]. However, this study did not include MITEs, warranting their careful examination and curation in future research.

In genetic research, TE landscapes are used to quantify the sequence divergence between individual TE copies and a family consensus sequence. Histograms of the resulting Kimura distances [147] offer insights into TE activity over evolutionary timescales, serving as a proxy for divergence time [87, 148–150]. Our divergence landscape analysis delineates three temporal waves of TE mobilization, reflecting the dynamic history of the *D. virilis* genome. The first cluster comprises young, recently active families with low Kimura divergence, many of which are intact and enriched in euchromatin. The second cluster, with intermediate divergence, likely corresponds to mobilization events during the diversification of the *virilis* species group [54–56]. A similar pattern of TE distribution was observed in the genomic assemblies of closely related species of *D. americana* and *D. novamexicana*, and even the diverged subgroup of *D. montana* [49, 151]. Finally, the third, ancient cluster is dominated by degenerate copies of *Helitrons* and LTR retrotransposons, occupying the largest genomic territory and residing primarily in heterochromatin. This pattern of ancient TE invasions is pervasive among species of the genus Drosophila, as observed in *D. melanogaster* and other species groups, providing a retrospective view of the history of genome colonization by TEs [151–154].

### Heterochromatin as a dynamic reservoir for TEs

Pericentromeric heterochromatin, with its low meiotic recombination, is enriched with repetitive TEs and is considered a genomic repository for superfluous DNA [155, 156]. Euchromatic TE insertions, however, are less tolerated as they are likely subject to negative selection [157, 158].

The landscape of TE insertions is far from random. It is shaped by both sequence-level preferences, such as specific nucleotide motifs or structural features, and chromatin context, including accessibility, histone modifications, and nuclear architecture [159–161]. While some TEs prefer open chromatin, others target heterochromatin via host factor tethering [162–166]. The data presented in the current study also suggest that the repressive environment of heterochromatin is not a substantial constraint for the transposition of intact elements. Indeed, dozens of essential genes were identified in the pericentromeric heterochromatin of Drosophila [167]. The accumulation of full-length TEs in heterochromatin may be explained by epigenetic heterogeneity, where euchromatin-like islands lack the H3K9me3 mark, providing a platform for transcription factors [168, 169].

Pericentromeric heterochromatin forms 3D domains with euchromatin via liquid–liquid phase separation [169]. TEs, which recruit repressive marks like H3K9me2/3, may facilitate this spatial organization. The accumulation of TEs in heterochromatin suggests that these interactions are dynamic and could influence fitness, though whether this is due to insertion or retention bias remains unclear.

Certain TEs exhibit a preference for insertion into heterochromatin. A notable example is the *I-element* in *D. melanogaster*, which shows a strong tendency to integrate into pericentromeric regions under hybrid dysgenesis conditions [170]. To this end, in Drosophila, representatives of the *R1* superfamily of TEs, *R1* and *R2*, exhibit a preference for inserting into the ribosomal DNA cluster located in the pericentromeric regions of X and Y chromosomes [171].

In summary, the accumulated data challenge the paradigm that heterochromatin solely harbours degenerate repeats and suggest that TEs actively colonize these regions, potentially exploiting their unique chromatin environment.

### TE-derived tandem repeats as a source for piRNA production

Once dismissed as “junk” DNA, along with TEs, tandem repeats like satellite DNA are now known to be vital for chromosome structure, heterochromatin assembly, and development [172, 173]. Both TEs and satellite DNAs may produce piRNAs, linking them to genome defence. In *D. melanogaster*, complex satellite DNAs are transcribed into lncRNAs and processed into piRNAs via the Rhino-Deadlock-Cutoff complex [174]. The *Helitron-2*-derived 154-nt repeats demonstrate similar properties, being transcribed and processed into piRNAs. Given that the *D. virilis* genome is especially rich in repeats, including tandem repeats of various sizes [50, 51, 175], it raises key questions: what signals trigger the transcriptional activation of these repeats, and how does the piRNA machinery recognize different repeat classes? The *rhino^G31D^* mutation causes Rhino to redistribute from piRNA clusters to satellite repeats, altering the piRNA profile [176], highlighting the need to investigate differences in piRNA production from TE-derived versus satellite repeats.

### Strain-specific TE landscapes reveal dynamic regulation and a potential for genomic conflict

Our findings show that the *D. virilis* genome contains diverse TE families, with RC elements (*Helitron-1* and *Helitron-2*) being the most abundant, consistent with prior reports of their high copy number in Drosophila [3]. The presence of intact full-length copies of certain TE families (e.g. *Demodocus, Snarf, Agelaus, Galatea, Hecuba, Ziggy, Anticlea, Telemac, Andromache*, etc.) targeted by piRNAs in all strains suggests recent invasions. The conservation of these elements across the studied strains contrasts with the variable presence of other families like *Penelope* and *Polyphemus*, that show strain-specific patterns probably reflecting different evolutionary histories or selective pressures in these strains. The predominance of truncated copies demonstrated for most TE families is consistent with the high rate of DNA loss documented in other Drosophila genomes [177, 178].

The dominance of LINE elements *Demodocus* and *Rachael* in strains *101* and *1051.47* contrasts with the prevalence of LTR retrotransposons in strains *9, 140*, and *160*. This biased TE family representation mirrors patterns in *Drosophila simulans*, where LTR retrotransposons have proliferated in specific lineages through episodic bursts [179]. While these bursts may arise spontaneously, they are frequently linked to stressful conditions, including extreme temperatures, irradiation, chemical exposure, or viral infection [180–184]. Since some TEs, such as *Copia*, have been found to contain sequences homologous to the promoters of heat shock protein genes [185], it will be important to investigate TE expression and potential mobilization in *D. virilis* after heat shock, to identify responsive TEs and adaptive insertions that may contribute to stress adaptation.

Based on its unique TE composition, strain *160* is an established “inducer” of *D. virilis* hybrid dysgenesis [58, 142]. The uneven distribution of active TEs like *Paris* and *Helena* between strains explains the paternal transmission of TEs to naïve maternal genomes, causing germline mobilization and sterility in certain crosses [62, 84]. Our finding of higher TE expression in strains where they are more prevalent supports their direct role in this syndrome.

The interstrain variations in TE composition have important evolutionary implications. Although hybrid dysgenesis may be rare in nature, the underlying “genomic shock” comprising TE activation, novel insertions, and the resulting epigenetic and transcriptional changes can occur on a more localized scale. Low-level genomic turmoil, driven by differential TE activity, provides a continuous source of genetic variation that can contribute to population differentiation and adaptation. Strain-specific TE patterns may even contribute to reproductive isolation through genomic incompatibilities [186, 187]. Future work should explore how these TE differences translate into phenotypic variation and population divergence in *D. virilis*.

The observed significant correlation between total TE copy number (full-length + truncated) and expression levels suggests that both types of copies contribute to the TE transcript pool, possibly through the transcription from cryptic promoters, flanking gene promoters or the presence of internal promoters in truncated elements [188, 189]. It is also important to note that truncated TEs can be “hidden” within chimeric gene–TE transcripts. Because they are controlled by the gene’s own promoter, their expression level is not a true measure of the TE’s activity [190, 191]. An alternative explanation is that the correlation arises from genomic co-occurrence. While both full-length and truncated copies are linked to TE expression, this may be a coincidence of their shared location rather than evidence that truncated copies are directly involved in transcription.

### HT and functional decay: modes of TE persistence

Evidence from *D. melanogaster* demonstrates that lineages of recently mobilized TEs are frequently shared among close relatives through HT [128, 129]. Consequently, a TE’s ecosystem can be viewed as encompassing the genomes of close relatives, suggesting adaptation to a broader host group rather than a single species.

The most well-described TE in *D. melanogaster* is the *P-element*. It invaded *D. melanogaster* from *D. willistoni* via HT and can cause hybrid dysgenesis [104, 192]. Since this discovery, it has become increasingly clear that HT is an important source of newly invading TEs [105, 193]. Two factors facilitating transfer are ecological proximity, as with the *P-element*’s spread during New World colonization, and phylogenetic relatedness, where TEs pre-adapted to one host can readily function in a related host within a similar cellular environment [194].

We found substantial evidence of HT of 13 TE families among species of the virilis group, including between phylads that diverged ∼9 million years ago. More broadly, these results support the hypothesis that a single species is not the entire ecosystem for TE lineages. Rather, groups of related species are, with recurrent invasion, loss, and re-invasion. This pattern of sharing among related species is consistent with a recent study that identified phylogenetic relatedness as an important factor in TE HT rates [195].

Conversely, within a genome, TEs are subject to mutational decay. Crucially, our ORF integrity analysis revealed that a significant proportion (∼30%–50%) of full-length TE insertions contain disruptive mutations, rendering them nonfunctional. This highlights that the mere presence of a full-length sequence is an insufficient proxy for transposition competence; a comprehensive assessment must integrate structural and coding potential to distinguish active TEs from genomic relics.

The predominance of retrotransposon families (*Galatea, Hecuba, Andromache, Rachael*, etc.) among TEs with complete ORFs is particularly noteworthy. This pattern may reflect several biological phenomena. While HT imposes purifying selection on all TEs, the selective pressures during within-host evolution differ dramatically between TE classes. Retrotransposons predominantly evolve under purifying selection within host genomes, a pattern consistent with the *cis*-preference of their replication mechanism, which directly links a copy’s transposition to the functionality of its own proteins [193]. In contrast, DNA transposons evolve neutrally within hosts due to *trans*-complementation, which decouples a copy’s replication from its own coding capacity [193]. This fundamental difference in selection regimes explains why the long-term persistence of DNA transposons relies more heavily on HT than that of retrotransposons. Across a broad phylogenetic scale of animals, the accumulation of TEs in genera is not consistently linked to effective population size [196]. This suggests that long-term genome size evolution is not predominantly driven by genetic drift, but rather depends on lineage-specific factors such as host silencing mechanisms and deletion biases [196].

### A TE-induced inversion breaks karyotypic stasis in ***D. virilis***

Inversions, which are widespread in Diptera species including *D. melanogaster* populations, are often caused by TEs [3, 134]. While most TE-induced inversions are deleterious, some become fixed due to selective advantages [197]. Inversions were also shown to play a significant role in the evolution and speciation of the *virilis* species group [48, 49]. However, *D. virilis* is an exception within the *virilis* group, showing a highly stable karyotype.

Our study identifies the first spontaneous chromosomal inversion in *D. virilis* (strain *1051.47*), a ∼4.5 Mb event linked to *Rachael* and *Hecuba* retrotransposon insertions at its proximal breakpoint, connecting retrotransposon activity to macro-evolutionary change. The distal breakpoint lacked such evidence, possibly due to transposon loss. Alternatively, the inversion may have arisen from misrepair of a transposon-induced double-strand break via nonhomologous end joining, a mechanism prevalent in the *D. melanogaster* group [198]. While spontaneous inversions were not reported in *D. virilis*, numerous inversions and other aberrations with TE-associated breakpoints have been found in this species in the progeny of dysgenic crosses [48].

Inversions may influence speciation by maintaining co-adapted gene complexes via suppressed recombination in heterozygotes [199, 200]. For instance, inversions at the X chromosome were shown to reduce gene flow between *Drosophila flavomontana* and *D. montana*, supporting their role in reinforcing species boundaries [201]. Future studies could investigate how inversion polymorphisms contribute to local adaptation in *D. virilis*.

### TEs as drivers of gene regulation

Numerous studies have demonstrated how TE insertions may influence nearby gene expression, with a few even providing adaptive advantages [31, 202–206]. In this study, we show that the spread of H3K9me3 from TEs is predominantly associated with reduced gene expression, but only when the TE is inserted in a promoter or exon. H3K9me3 spreading extends up to 4 kb from full-length TE insertions, consistent with *D. melanogaster* studies showing that heterochromatin spreading is distance-limited [29]. In contrast, in the *Drosophila nasuta* species group, H3K9me3 enrichment is detected near TE insertion sites, both upstream and downstream, but rapidly diminishes within 100 bp [39]. Variation in H3K9me3 spreading patterns across Drosophila species likely reflects differences in species-specific genomic and epigenetic regulation. Similar distribution patterns observed in *D. virilis* and *D. melanogaster* suggest the utilization of shared chromatin remodellers such as Heterochromatin proteins 1 (HP1) and Su(var) proteins or RNA interference pathways that reinforce H3K9me3 over larger distances [19, 20]. In contrast, the narrow H3K9me3 spreading boundaries demonstrated in *D. nasuta* species group probably imply stronger barriers to spreading, including insulator elements that may block the spreading, competing euchromatin histone marks (e.g. H3K4me3). The demonstrated difference in the spreading between Drosophila species may also be due to the expansion of species-specific Drosophila interspersed elements observed in the *D. nasuta* species group that may lack strong heterochromatin nucleation signals (e.g. piRNA targeting) [39].

In contrast to *D. virilis*, in *D. melanogaster*, more than half of the analysed euchromatic TEs are associated with the spread of repressive epigenetic marks by at least 1 kb and do not show differences in spreading distance, even between species of *D. melanogaster* subgroup [29, 37]. However, a recent genome-wide, multitissue analysis [31] reveals this broad-stroke view obscures critical nuances. The epigenetic effects of TEs are not monolithic but are profoundly dependent on genomic context and tissue type. For instance, the repressive effect was strongest in the ovary but significantly weaker in the head, linked to the expression of chromatin regulators [31]. Therefore, the variation in spreading potential observed in *D. virilis* is unlikely to reflect long-term evolutionary adaptive strategies. Rather, it arises from the complex interplay between chromatin architecture and the balance of TE suppression with gene expression fidelity, driven by the expansion of the most active TE families.

Beyond influencing gene expression via insertion and epigenetic spreading, DNA transposons can generate structural variation through imprecise excision [207]. Our analysis confirmed characteristic target site duplication (TSD) motifs (e.g. TA for *Paris* and *Polyphemus*, AT for *Alcinous*) at insertion sites. However, a genome-wide assessment did not reveal a strong correlation between these TE families and small, disruptive indels in CDS (data not shown).

In summary, while piRNA-mediated silencing initiates heterochromatin formation, the extent of spreading depends on the length of TE insertions, genomic context, and probably specific properties of TE families. These epigenetic modifications influence developmental and metabolic genes, providing potential fitness trade-offs in species evolution. However, it is important to note that our evaluation of TE-driven epigenetic changes and their functional effects on gene expression probably falls short of the real effects in natural populations. The study used long-established laboratory fly strains, where small population sizes and reduced selective pressure may alter TE-gene dynamics. Furthermore, our analysis was limited to homozygous insertions in ovarian tissue, unlike the heterozygous insertions found across various tissues and developmental stages in wild populations.

## Conclusion

Since the discovery of its extensive satellite DNA content by Joe Gall and colleagues in the 1970s [51], *D. virilis* has been an important model for understanding the evolution and function of repetitive DNA. Our study provides a comprehensive description of TE dynamics in *D. virilis*, integrating genomic, epigenomic, and transcriptomic analyses across several strains to uncover the evolutionary and regulatory impact of TEs. By creating a refined TE library, we demonstrate that, despite the potential to disrupt genome stability, TEs persist as active contributors to genetic diversity, with their activity patterns reflecting both recent and ancient invasions. The interplay between TE mobilization and host silencing mechanisms, such as piRNA-mediated repression, demonstrates a delicate balance that shapes genome evolution. The study elucidates TE distribution across chromatin domains, their strain-specific variations, and their epigenetic effects on neighbouring genes. The observed heterogeneity in TE activity and silencing across strains suggests that TEs may contribute to population differentiation and adaptation. We also show recurrent HT of TEs within the *virilis* species group, supporting earlier observations of ongoing TE influx [208] and suggesting that closely related species act as a common ecosystem for TE lineages. Furthermore, the discovery of a chromosomal inversion linked to TE activity in *D. virilis* underscores the broader implications of TEs in genomic rearrangements and speciation. Our work establishes TEs as key drivers of genomic diversity and regulation. Future research should explore the functional consequences of TE-mediated regulation and expand comparative analyses across the *virilis* group including populations recently collected in the wild.

## Supplementary Material

gkag139_Supplemental_Files

## Data Availability

High-throughput sequencing data performed in this study includes RNA-seq for strains *160, 101*, and *1051.47*; small RNA-seq for strains *160, 9, 101, 140, 1051.47*; ChIP-seq (H3K9me3) for strains *101, 1051.47*; ChIP-seq (H3K9me2) for strain *140*; raw ONT reads for strains *160, 9, 101*, and *1051.47* were deposited in NCBI GEO under the number GSE307819. The accession number for ChIP-seq (H3K9me3) for strains *160* and *9* in NCBI GEO is GSE59965. ChIP-seq, RNA-seq for strain *140*, as well as RNA-seq for strain *9*, can be downloaded from NCBI GEO using the number GSE292339. Raw ONT reads for strain *140* were deposited in the NCBI SRA (SRX28458524).

## Notes

Present address: Institute of Molecular Biotechnology of the Austrian Academy of Sciences (IMBA), Vienna BioCenter (VBC), Dr. Bohr-Gasse 3, 1030 Vienna, Austria
