# Supplementary material for: Transposable elements as drivers of genome evolution in Drosophila virilis
Source: Nucleic Acids Res. 2026 Feb 17;54(4):gkag139. doi: 10.1093/nar/gkag139 (PMC12910113; doi:10.1093/nar/gkag139)
Supplement: gkag139_Supplemental_Files [file gkag139_supplemental_files.zip › Supplementary_materials.docx]

**Supplementary materials to the manuscript entitled “Transposable elements as drivers of genome evolution in *Drosophila virilis*”**

Alexander P. Rezvykh^1^, Dina A. Kulikova^2^, Elena S. Zelentsova^1^, Liudmila Protsenko^1#^, Alina V. Bespalova^1^, Iuliia O. Guseva^3^, Justin P. Blumenstiel^4^, Mikhail B. Evgen’ev^1,5^, and Sergei Y. Funikov^1*^

^1^ Engelhardt Institute of Molecular Biology of Russian Academy of Sciences, Moscow, 119991, Russian Federation

^2^ Koltzov Institute of Developmental Biology, Russian Academy of Sciences, 119334 Moscow, Russia

^3^ Moscow Center for Advanced Studies, Kulakova Str. 20, 123592 Moscow, Russia

^4^ Department of Ecology and Evolutionary Biology, University of Kansas, Lawrence, Kansas, 66049, United States of America

^5^ Institute of Evolution, University of Haifa, Haifa 3498838, Israel

# present address: Institute of Molecular Biotechnology of the Austrian Academy of Sciences (IMBA), Vienna BioCenter (VBC); Dr. Bohr-Gasse 3, 1030 Vienna, Austria

* To whom correspondence should be addressed: Sergei Y. Funikov, e-mail: sergeifunikov@mail.ru

**Supplementary materials include:**

**Supplementary Files 1,2 (given as separate files)**

**Supplementary Tables S1-S3 (given as separate files)**

**Supplementary Figure S1-S15**

**Extended Materials and Methods**

**Supplementary figure and tables legends**

**Supplementary Table S1.** An overview of the numbers of TEs in the genome of *D. virilis*.

**Supplementary Table S2.** The TEs of *D. virilis* and their copy number.

**Supplementary Table S3.** The effect of TEs on gene expression.

**Supplementary Figures**

**
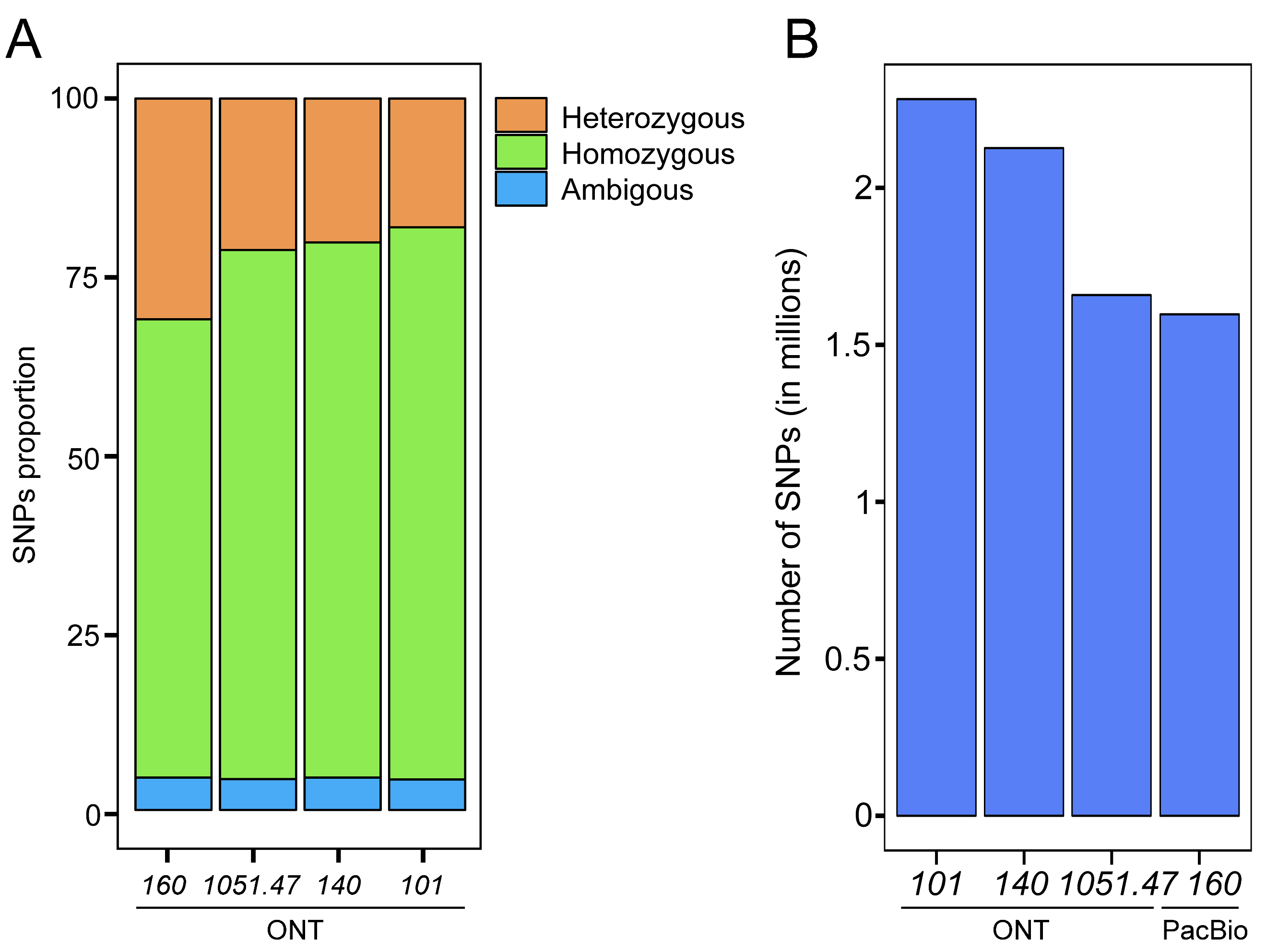
**

**Supplementary Figure S1.** (**A**) Homo- and heterozygous SNP levels in the studied strains. The heterozygosity levels were measured as the proportion of homozygous and heterozygous SNPs in four genomes. The genome of strain *9* was used as reference. ONT sequencing data was used for strain *160*. (**B**) The number of SNPs identified by comparative analysis of the genome assemblies (PacBio and ONT). Only SNPs located within the gene loci were taken into account. The genome of strain *9* was used as a reference for both analyses.

**
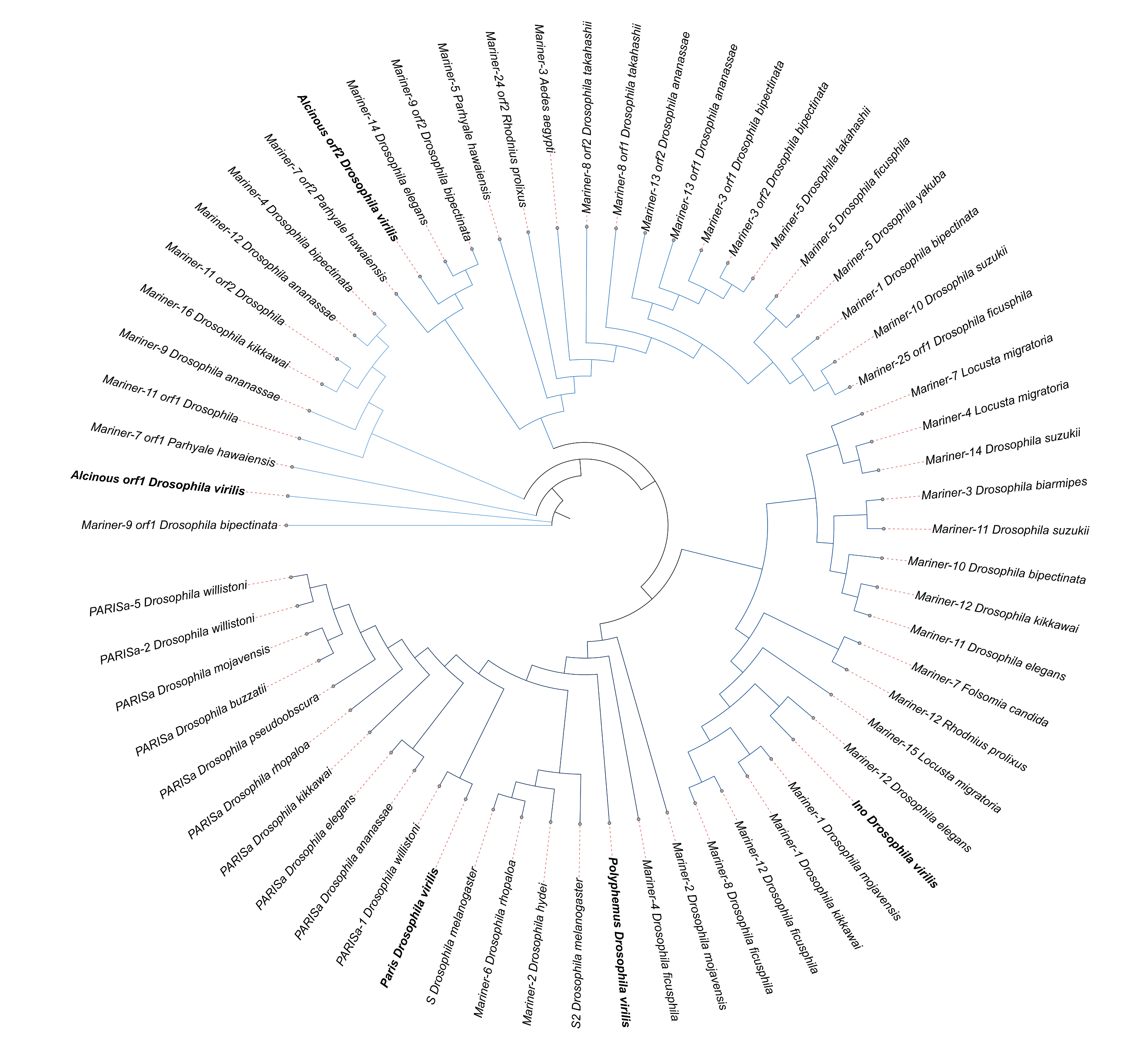
**

**Supplementary Figure S2.** Phylogenetic relationships between DNA transposon families of *D. virilis* and other invertebrates. The Bootstrap consensus phylogenetic tree is based on maximum likelihood topology. A phylogram is based on the amino acid alignment of full-length ORFs. The confidence of each node in the phylogenetic tree was determined using the bootstrap test with 100 replicates. TEs of *D. virilis* are shown in bold type.

**
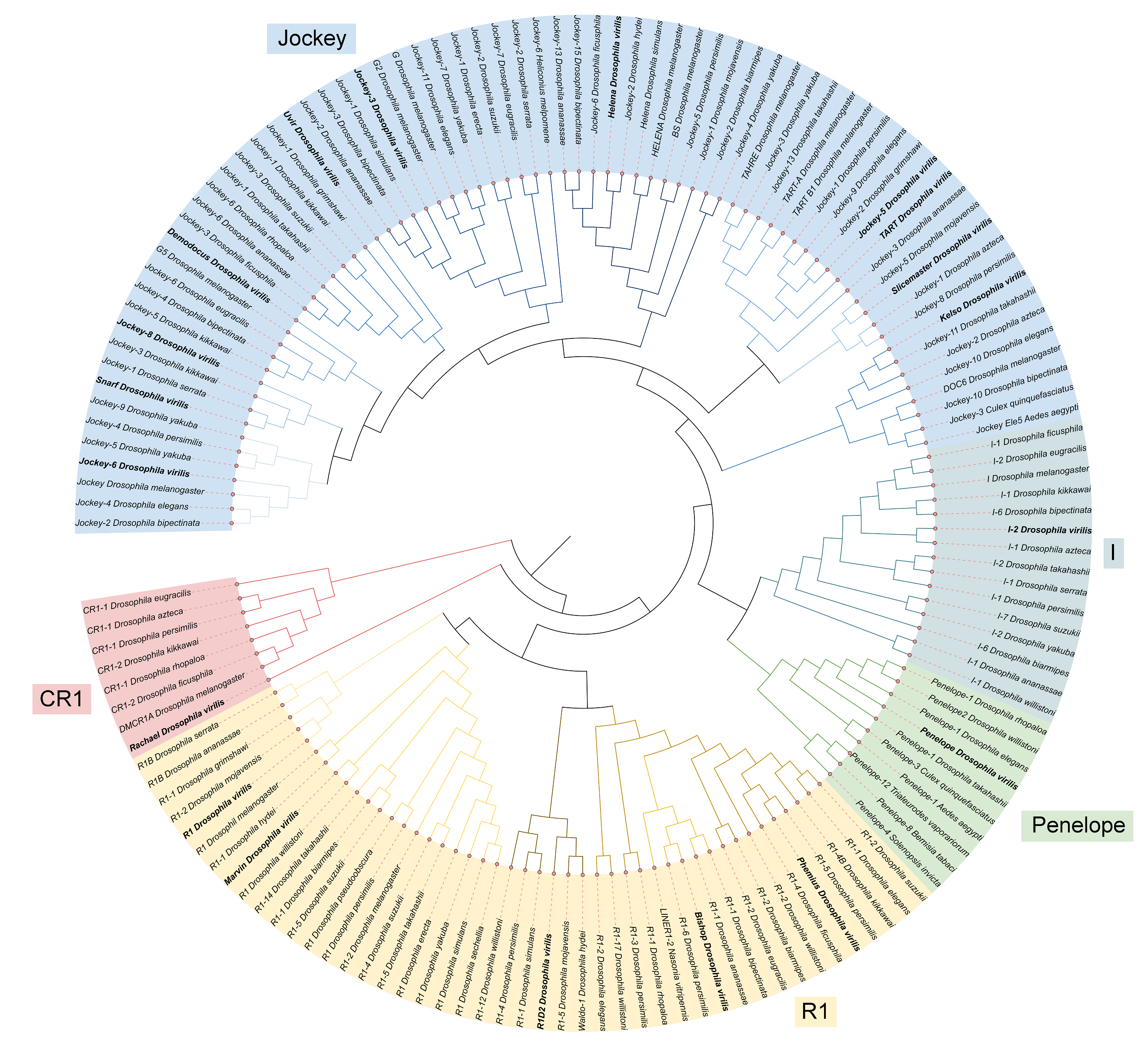
**

**Supplementary Figure S3.** Phylogenetic relationships between LINE retrotransposons of *D. virilis* and other invertebrates. The Bootstrap consensus phylogenetic tree is based on maximum likelihood topology. The phylogram is based on the amino acid alignment of full-length ORFs encoding endonuclease and reverse transcriptase domains. The confidence of each node in phylogenetic trees was determined using the bootstrap test with 100 replicates. TEs of *D. virilis* are shown in bold type.

**
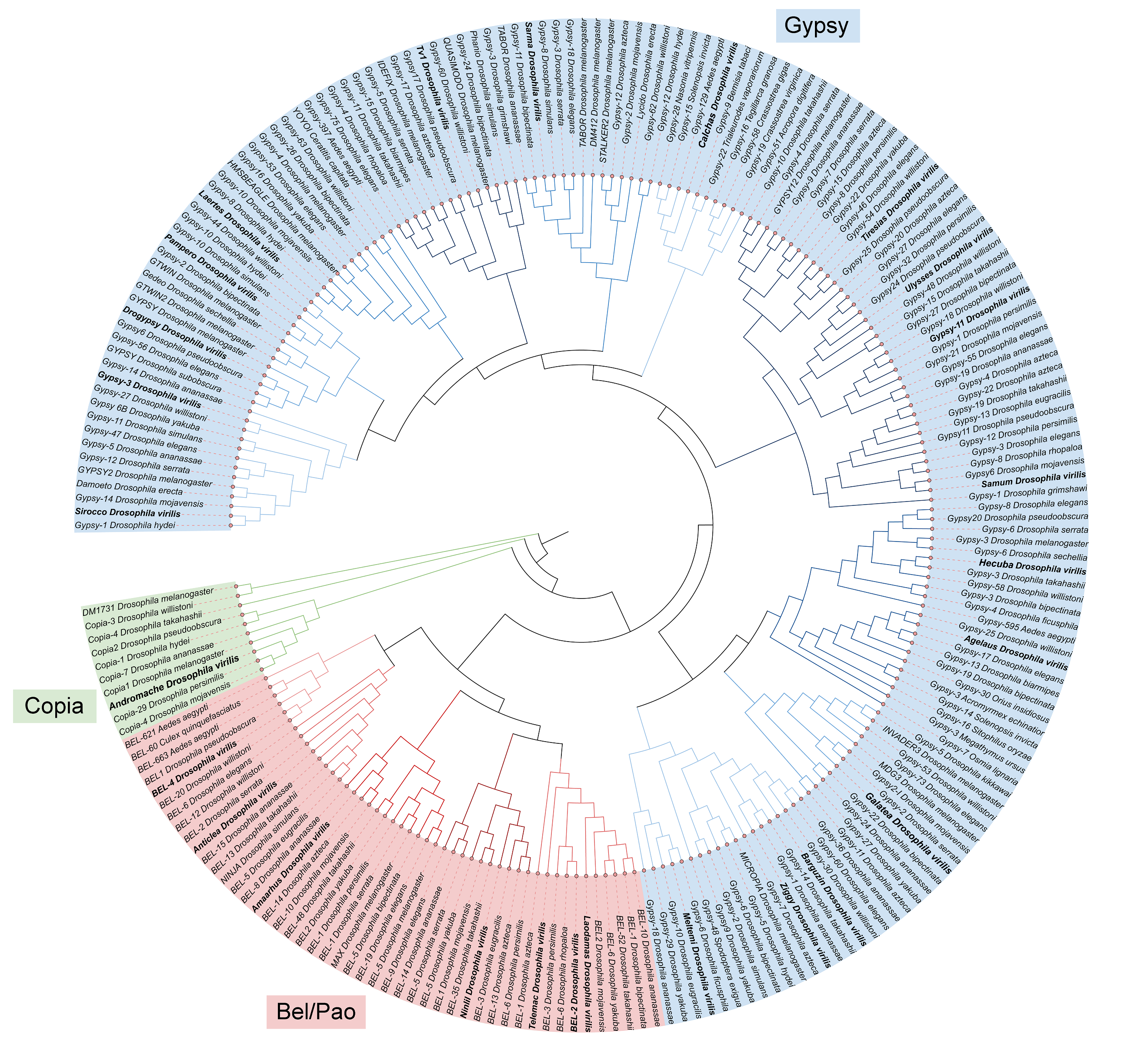
**

**Supplementary Figure S4.** Phylogenetic relationships between LTR retroelements of *D. virilis* and other invertebrates. The Bootstrap consensus phylogenetic tree is based on maximum likelihood topology. The phylogram is based on the amino acid alignment of full-length ORFs encoding endonuclease and reverse transcriptase domains. The confidence of each node in phylogenetic trees was determined using the bootstrap test with 100 replicates. TEs of *D. virilis* are shown in bold type.


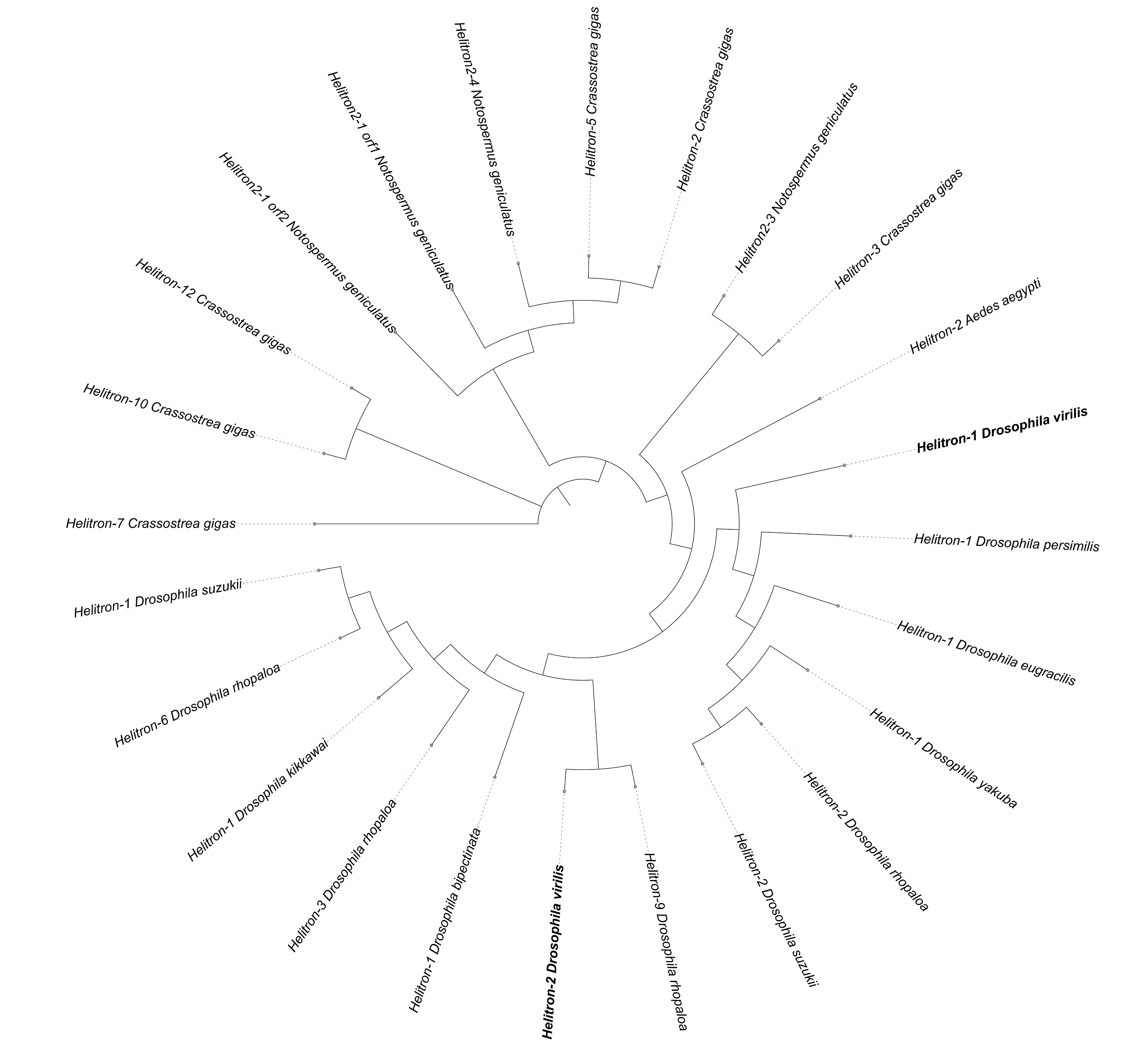


**Supplementary Figure S5.** Phylogenetic relationships between rolling-circle elements of *D. virilis* and other invertebrates. The Bootstrap consensus phylogenetic tree is based on maximum likelihood topology. A phylogram is based on the amino acid alignment of full-length ORFs. The confidence of each node in phylogenetic trees was determined using the bootstrap test with 100 replicates. TEs of *D. virilis* are shown in bold type.

**
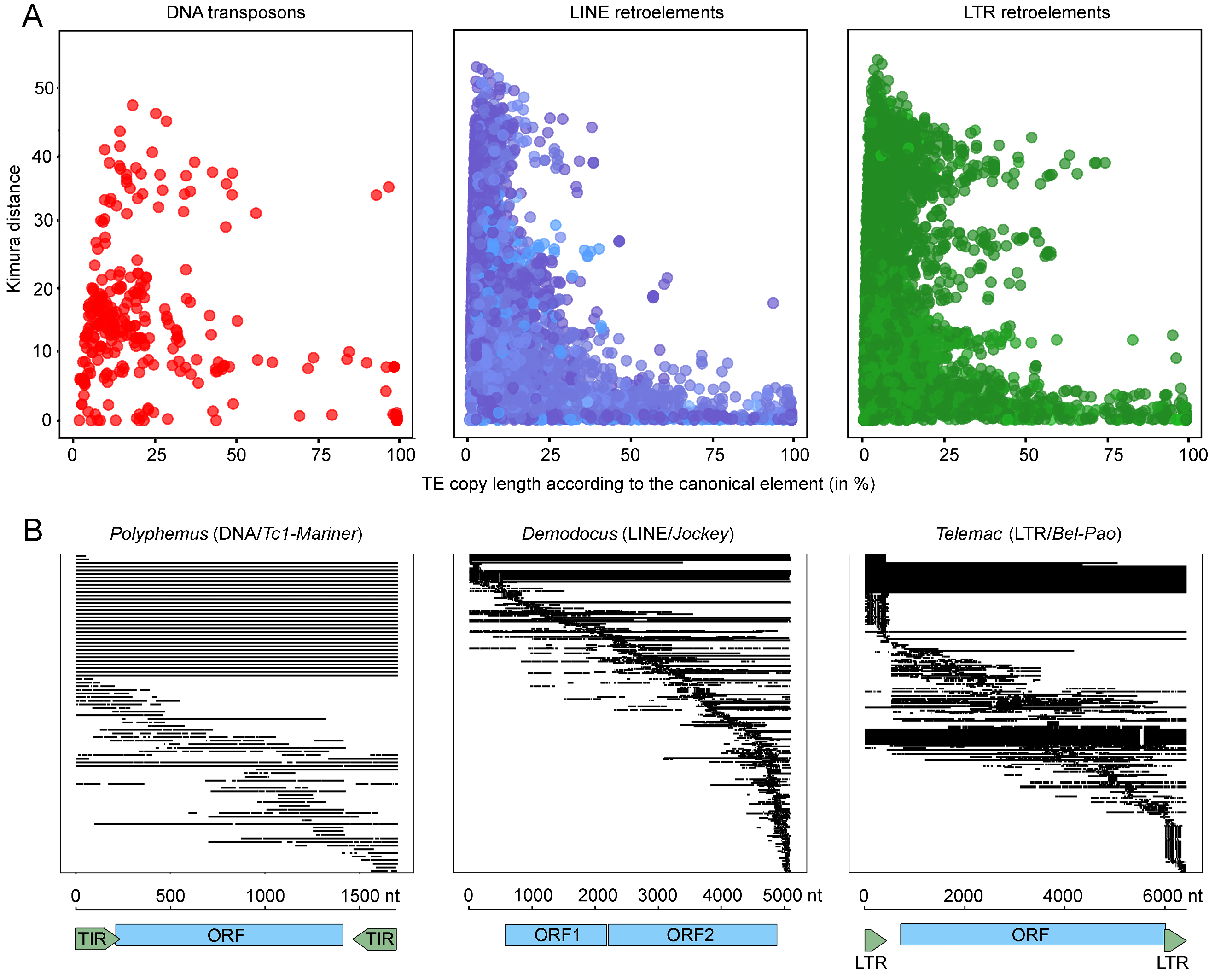
**

**Supplementary Figure S6.** Divergence of TE copies in the genome of *D. virilis*. (**A**) Relationship between TE insertion length and TE-divergence level (Kimura substitution level, adjusted for CpG) determined for each TE insertion. Only insertions of intact TE families were used for analysis. (**B**) Multiple sequence alignment of all genomic copies of DNA transposon *Polyphemus,* as well as retrotransposons *Demodocus* and *Telemac*. Data were analysed on the chromosome assembly of strain *160*.

**
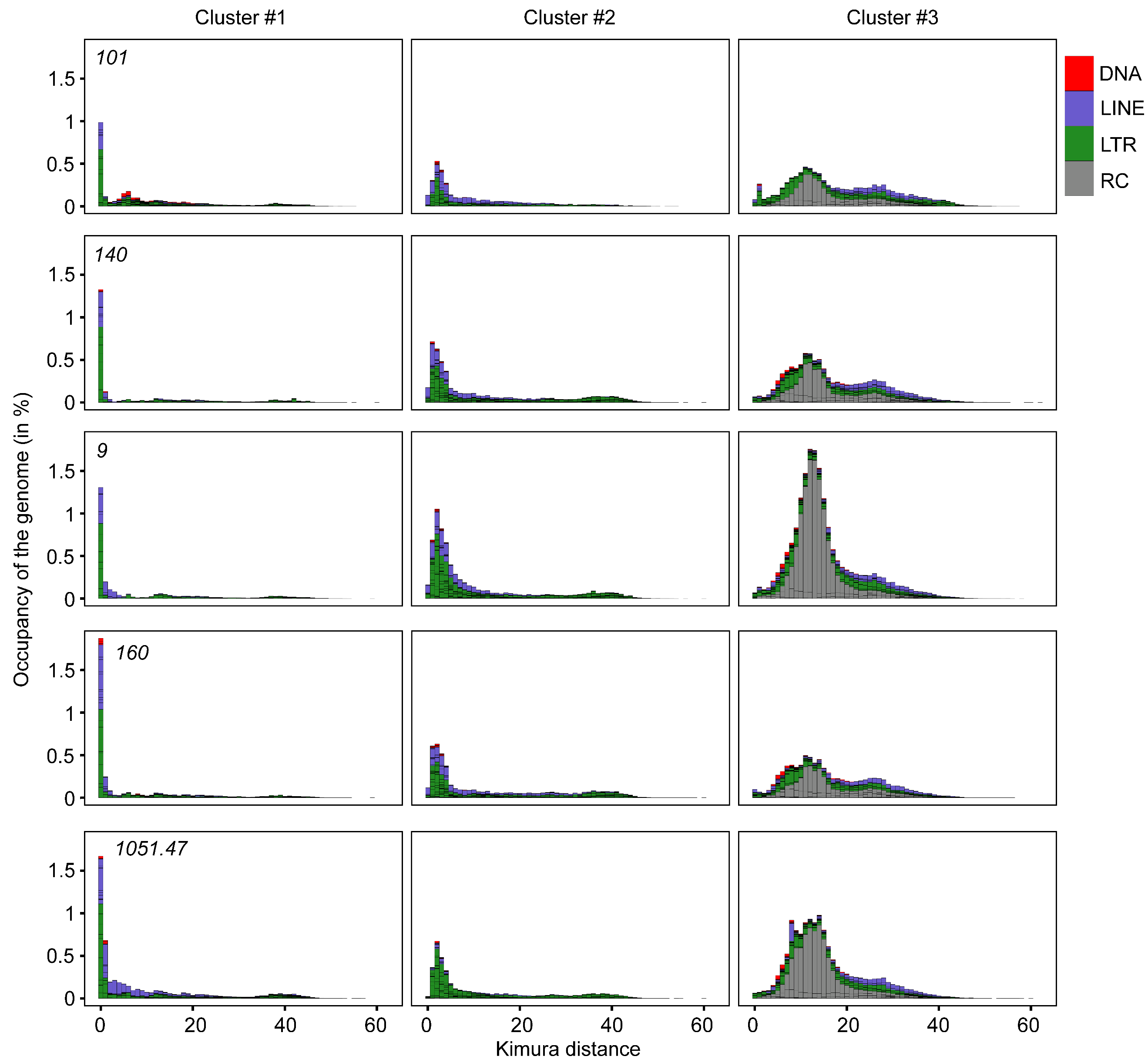
**

**Supplementary Figure S7.** TE divergence landscape in all studied *D. virilis* strains. The X-axes show the level of divergence (Kimura substitution level) between each identified TE copy and the consensus sequence for that TE family. The Y-axis shows the part of the genome occupied by each bar (in millions of bases covered). The number of TE clusters was determined by the silhouette method, followed by k-means clustering. Colours represent TE subclasses.


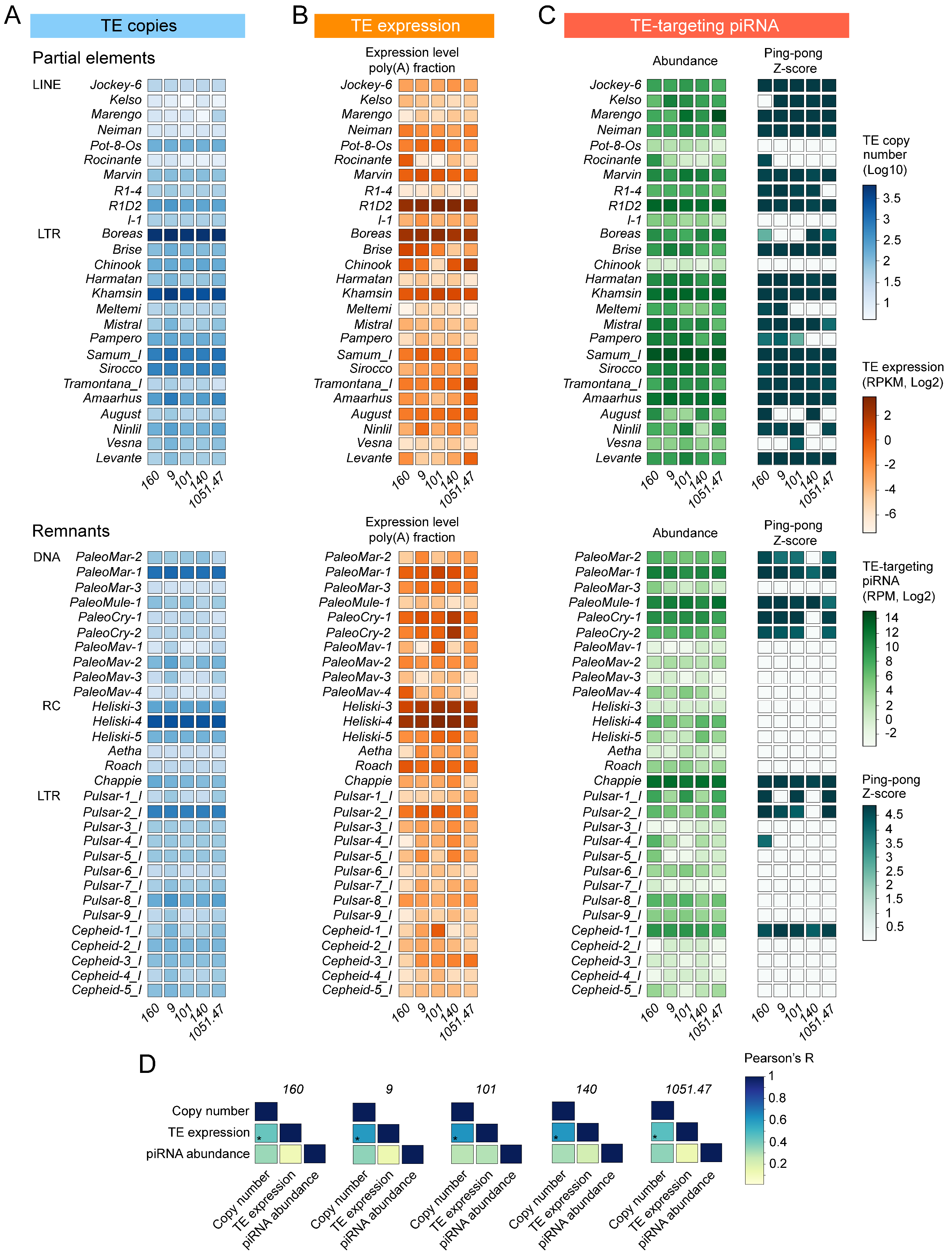


**Supplementary Figure S8.** Comparative analysis of TE abundance, TE expression, and TE-targeting by piRNAs in *D. virilis* strains (for partial and remnant elements). (**A**) Heatmap depicting the number of copies (Log10 values) of partial and remnant TEs in the genomes of strains studied. (**B**) Heatmap demonstrating the expression level (RPKM, Log2) for each TE family in ovaries of the studied strains. (**C**) Heatmaps showing the abundance of piRNAs (left) (RPM, Log2) and ping-pong Z-scores (right) for each TE family in five strains. (**D**) Pearson’s correlation coefficient between TE copy number, TE expression, and piRNA abundance is shown as a heatmap. Pairwise correlations were calculated between all categories for each strain studied and presented as average values. Asterisks indicate *p* < 0.05.


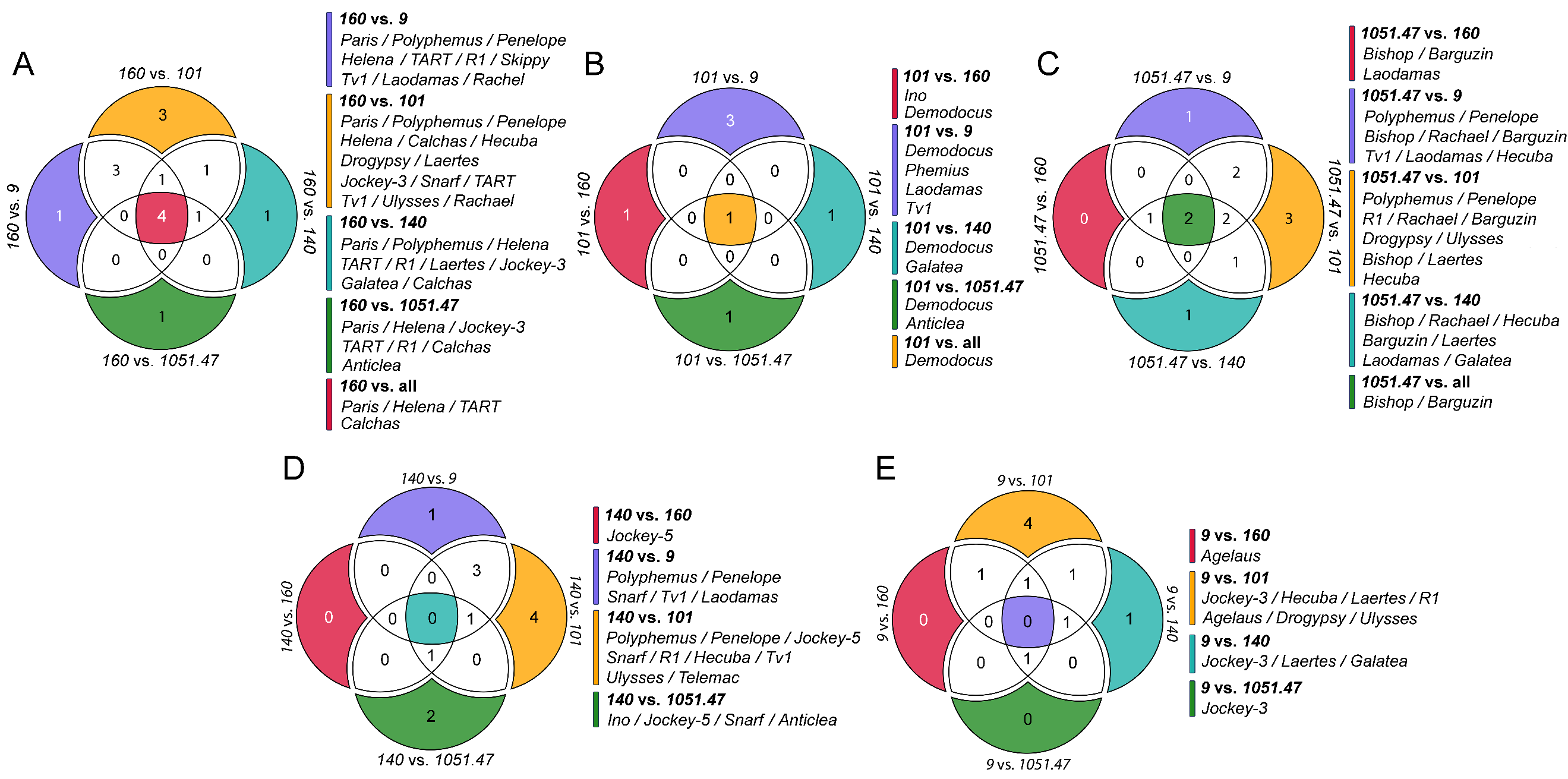


**Supplementary Figure S9.** Comparative analysis of TE copy number in *D. virilis* strains. (**A-E**) Venn diagrams show the results of pairwise comparisons of TE copy number in one particular strain compared to all others. Only full-length TE copies showing 2-fold differences in pairwise comparisons of fly strains were considered.

**
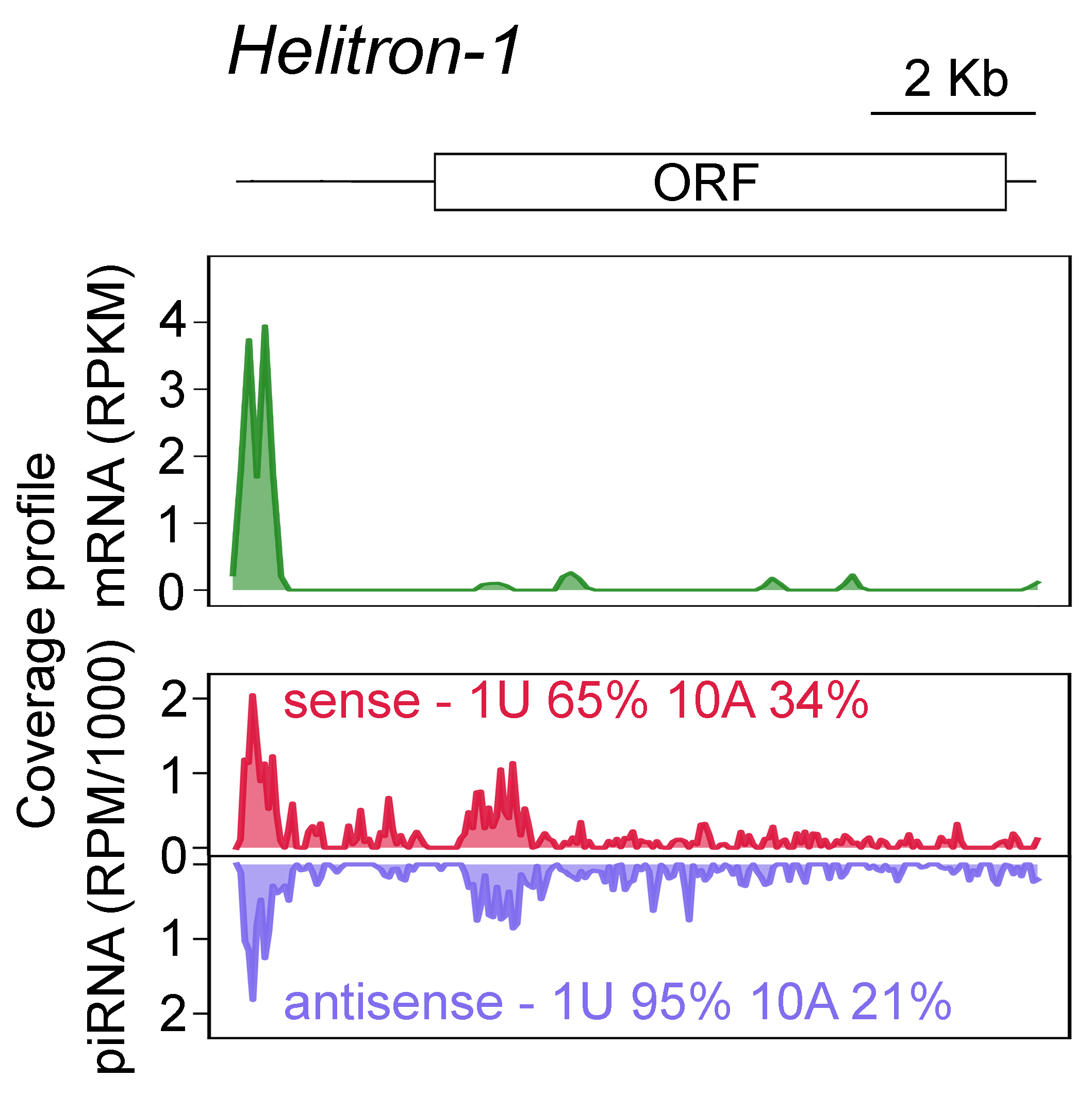
**

**Supplementary Figure S10.** Coverage profiles of RNA-seq and piRNA reads on *Helitron-1*. Data from strain *9* was used to plot RNA-seq and piRNA reads. Nucleotide biases, including 1U and 10A (percentage), are shown for both sense and antisense piRNA mappers (23-29 nt).

**
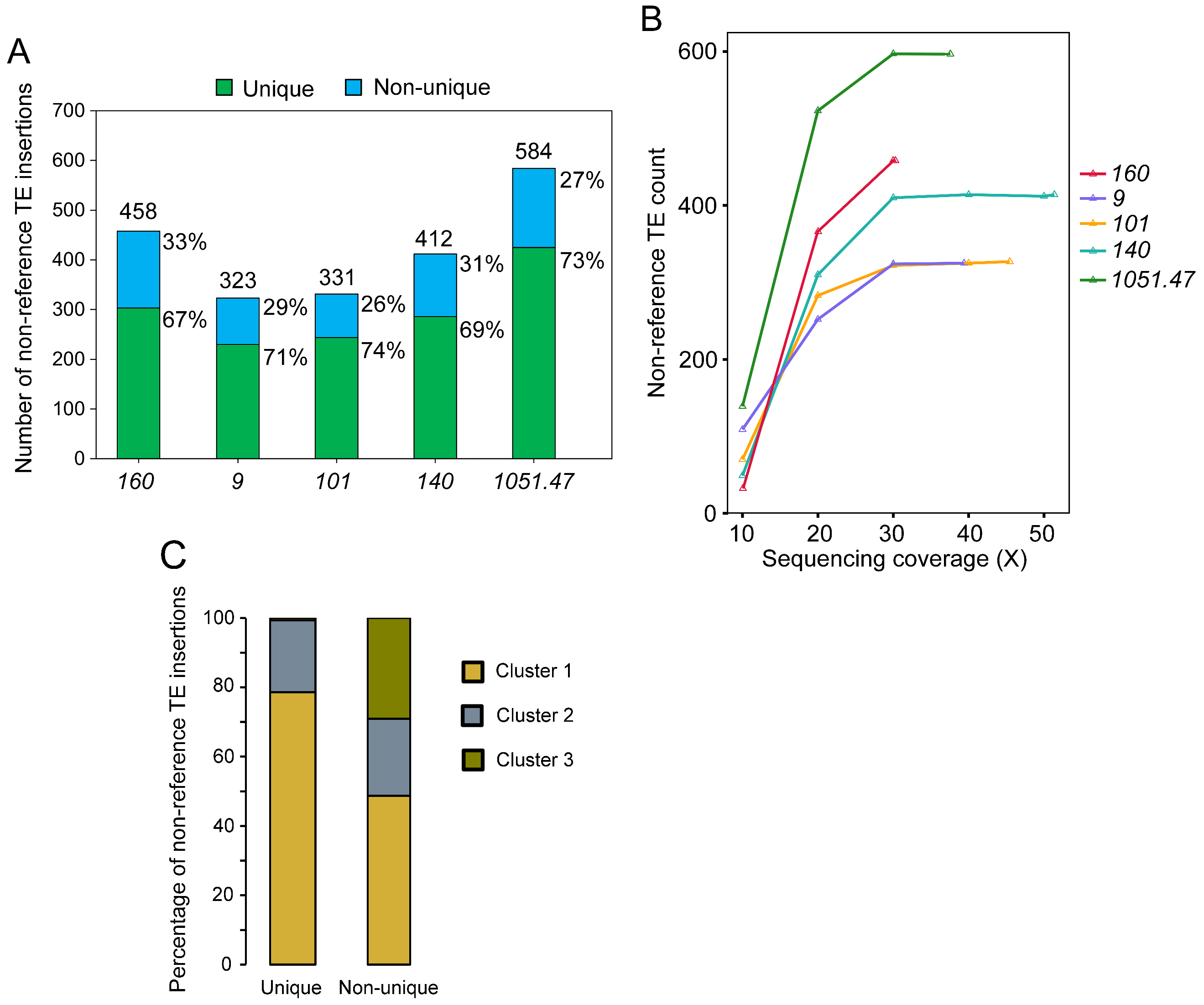
**

**Supplementary Figure S11.** Effect of sequencing coverage on the number of predicted non-reference TE insertions. **(A)** Comparison of the number of non-reference TE insertions predicted by the TELR software in strains *160*, *9*, *101*, *140,* and *1051.47.* TE insertions are subdivided into non-unique and unique, which are exclusively present in one strain and absent in others within a 10 Kb window centered on the TE insertion coordinate. **(B)** Comparison of the number of non-reference TE insertions identified using length- and depth-normalized long-read datasets for *D. virilis* strains *160*, *9*, *101*, *140,* and *1051.47*. Reads from all datasets were split into 10kb segments and sub-sampled to achieve the desired genome coverage by mapped reads. (**C**) Percentage of non-reference TE insertions as determined by Kimura distance cluster analysis presented in Figure 2.

**
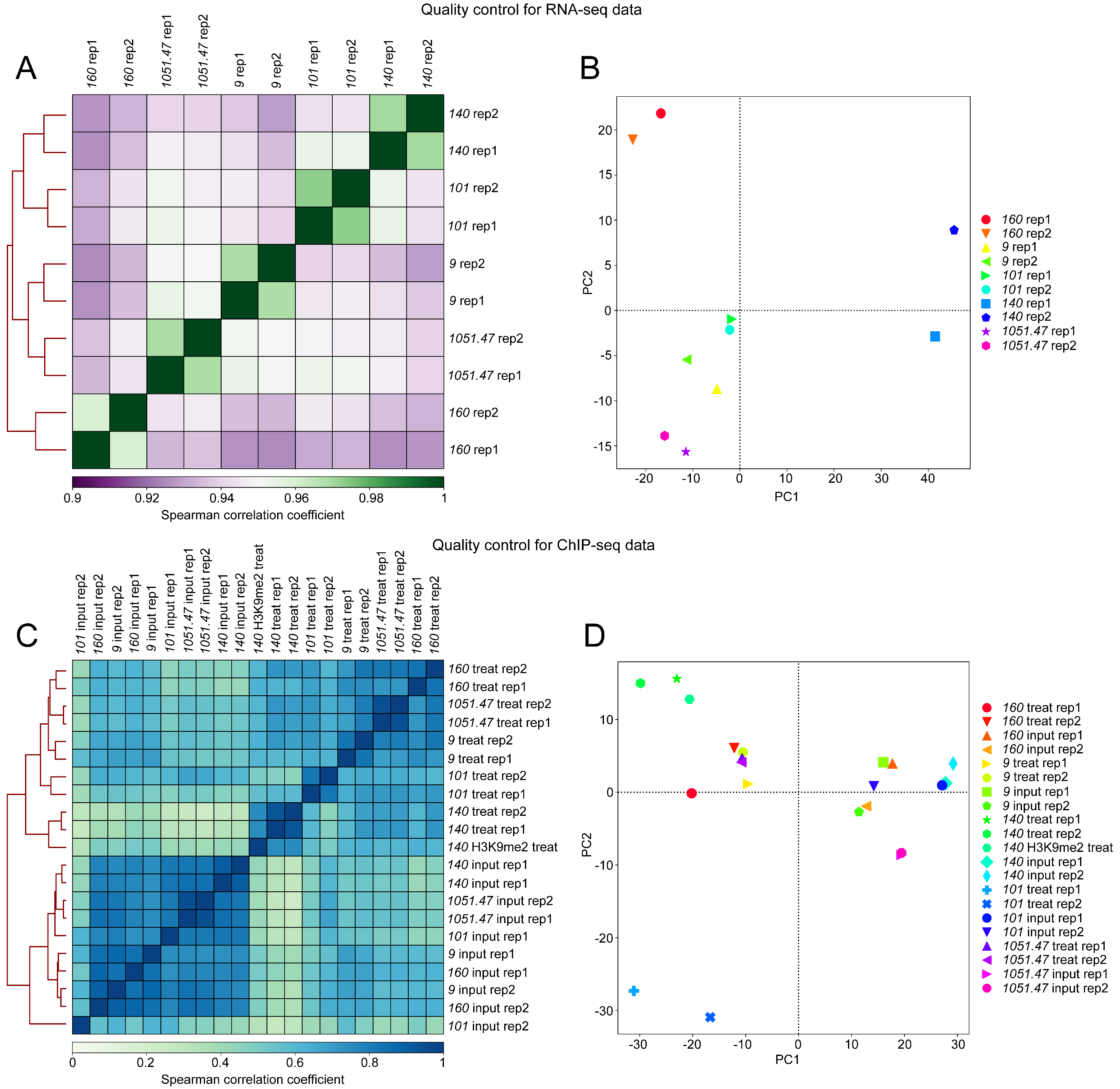
**

**Supplementary Figure S12.** Quality control for RNA-seq and ChIP-seq data used in the study. **(A)** and **(C)** The Spearman correlation coefficient (R) has been calculated for RNA-seq (A) and ChIP-seq (C) samples. Only genomic regions with unique TE insertions predicted by the TELR programme for each strain studied were used to calculate the average ChIP-seq score of mapped reads for each sample. The distances of the sample pairs are based on the correlation coefficients, where distance = 1 - R. H3K9me2 treat – ChIP of dimethylated H3K9, other treat samples – ChIP of trimethylated H3K9. **(B)** and **(D)** Principal component analysis performed for RNA-seq (B) and ChIP-seq (D) samples. The eigenvalues of the two principal components are shown. Only reads that matched annotated gene coordinates were used to calculate the mean score of RNA-seq samples.

**
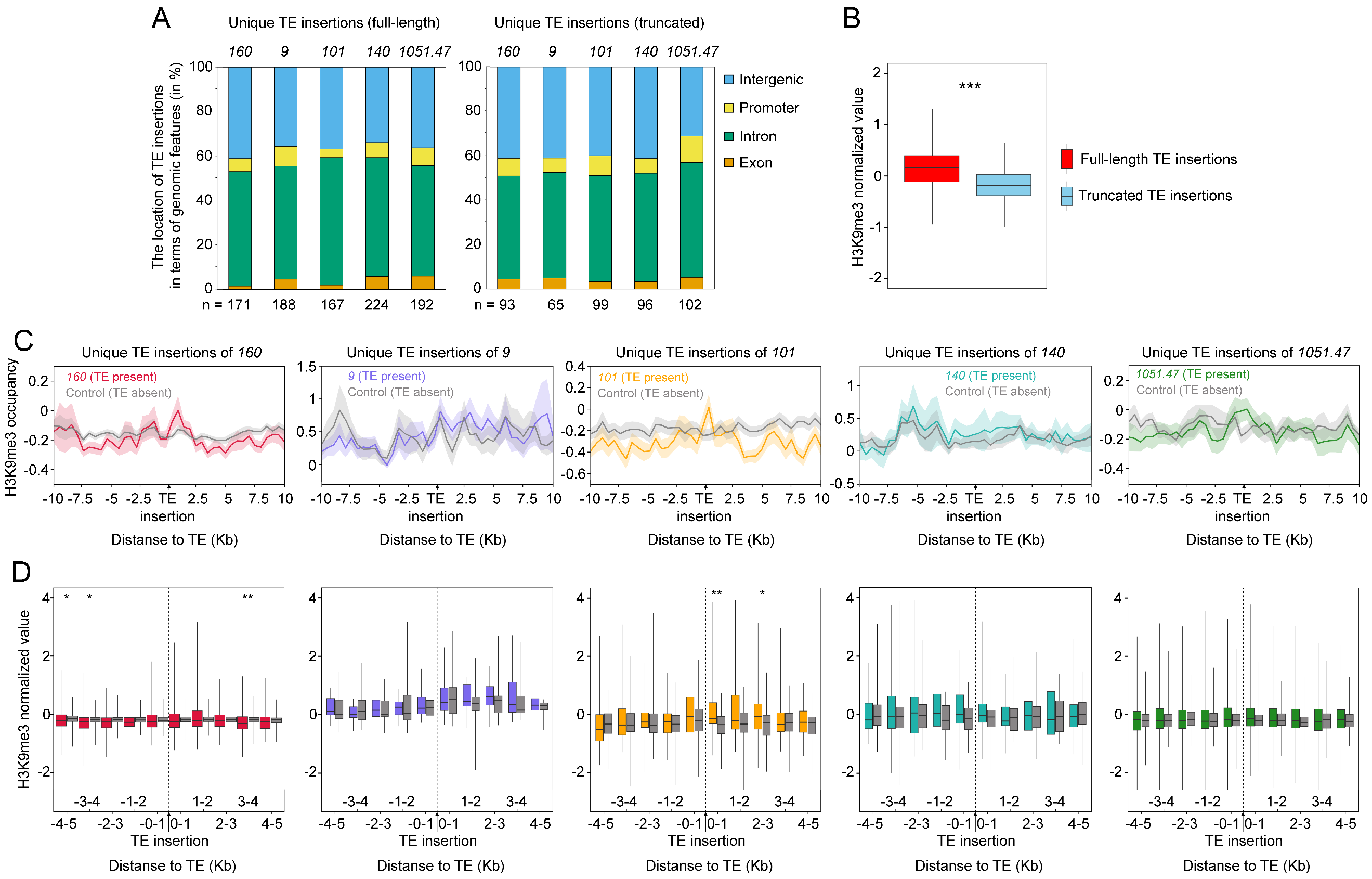
**

**Supplementary Figure S13.** Genetic and epigenetic effects of truncated or partial TE insertions on host gene expression. **(A)** The distribution of unique TE insertions, divided into full-length and truncated TE insertions, across genomic features is given for strains *160*, *9*, *101*, *140*, and *1051.47*. A region up to 1 Kb upstream of the transcription start site (TSS) of genes is considered the promoter region. **(B)** The genome-wide H3K9me3 occupancy around truncated/partial TE insertions in the studied genomes. The shaded areas indicate the 95% confidence interval. **(C)** The enrichment analysis of H3K9me3 occupancy in 0-1 Kb windows flanking TE insertions. The colours for the experimental group (TE present) and the control group (TE absent) correspond to those shown in B. Statistical significance was assessed by the Mann-Whitney U test, followed by FDR correction. * indicates *p* < 0.05 and ** denotes *p* < 0.01. **(D)** Boxplot depicts statistically significant difference in H3K9me3 enrichment between unique full-length and truncated TE insertions. In this analysis, all unique TE insertions were combined. *** indicates *p* < 0.001, as estimated by the Mann-Whitney U test, followed by FDR correction.

**
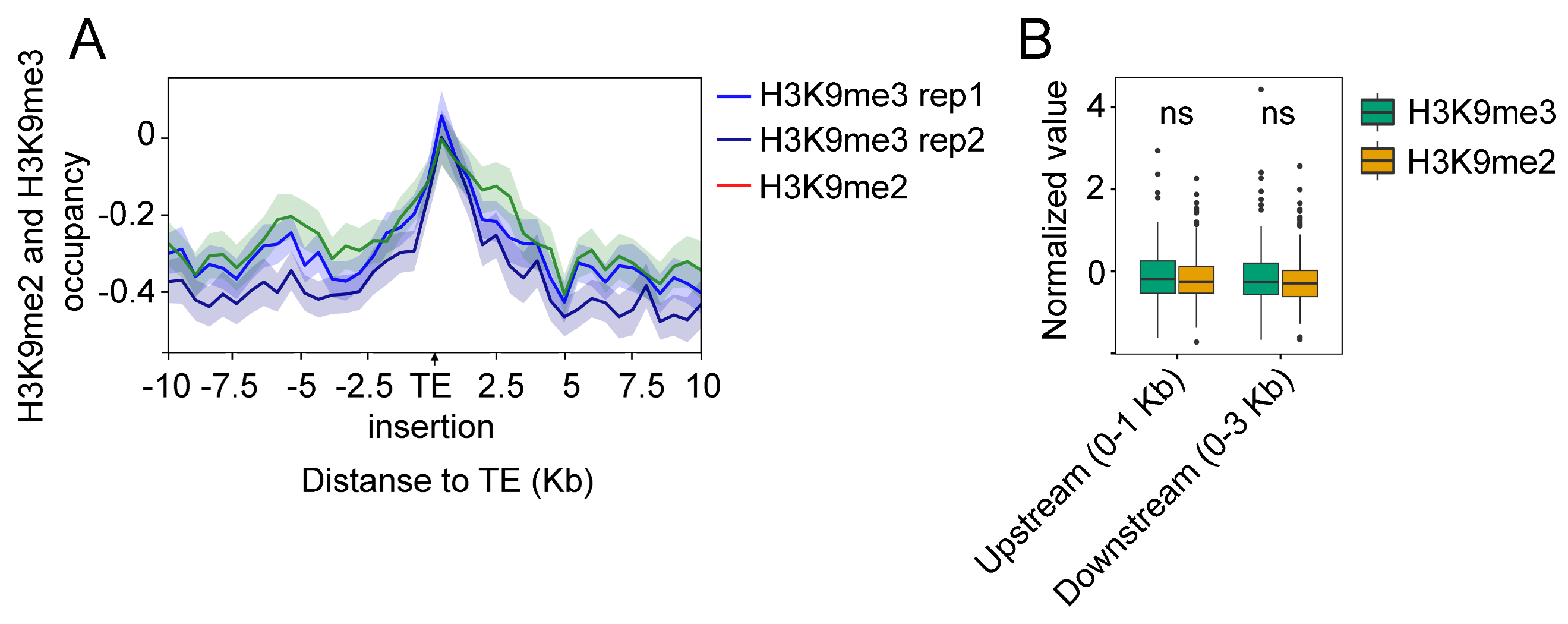
**

**Supplementary Figure S14.** Comparison of H3K9me2 and H3K9me3 spreading around TE insertions. **(A)** Comparison of upstream and downstream spreading of H3K9me2 and H3K9me3 from unique TE insertions observed in strain *140*. The values used to construct the distribution profile of both heterochromatic tags were calculated by subtracting the raw data from the treated (immunoprecipitated) samples. TE insertion regions were divided into 500 nt bins. **(B)** Boxplot shows no significant differences between the levels of H3K9me2 and H3K9me3 occupancy upstream and downstream of TE insertions. The TE insertion regions were divided into 50 nt bins, and then the enrichment of H3K9me2 and H3K9me3 in each bin was calculated. Values in the range 0-1 Kb and the range 0-3 Kb were used for upstream and downstream regions, respectively, consistent with the observed propagation profile in (A).


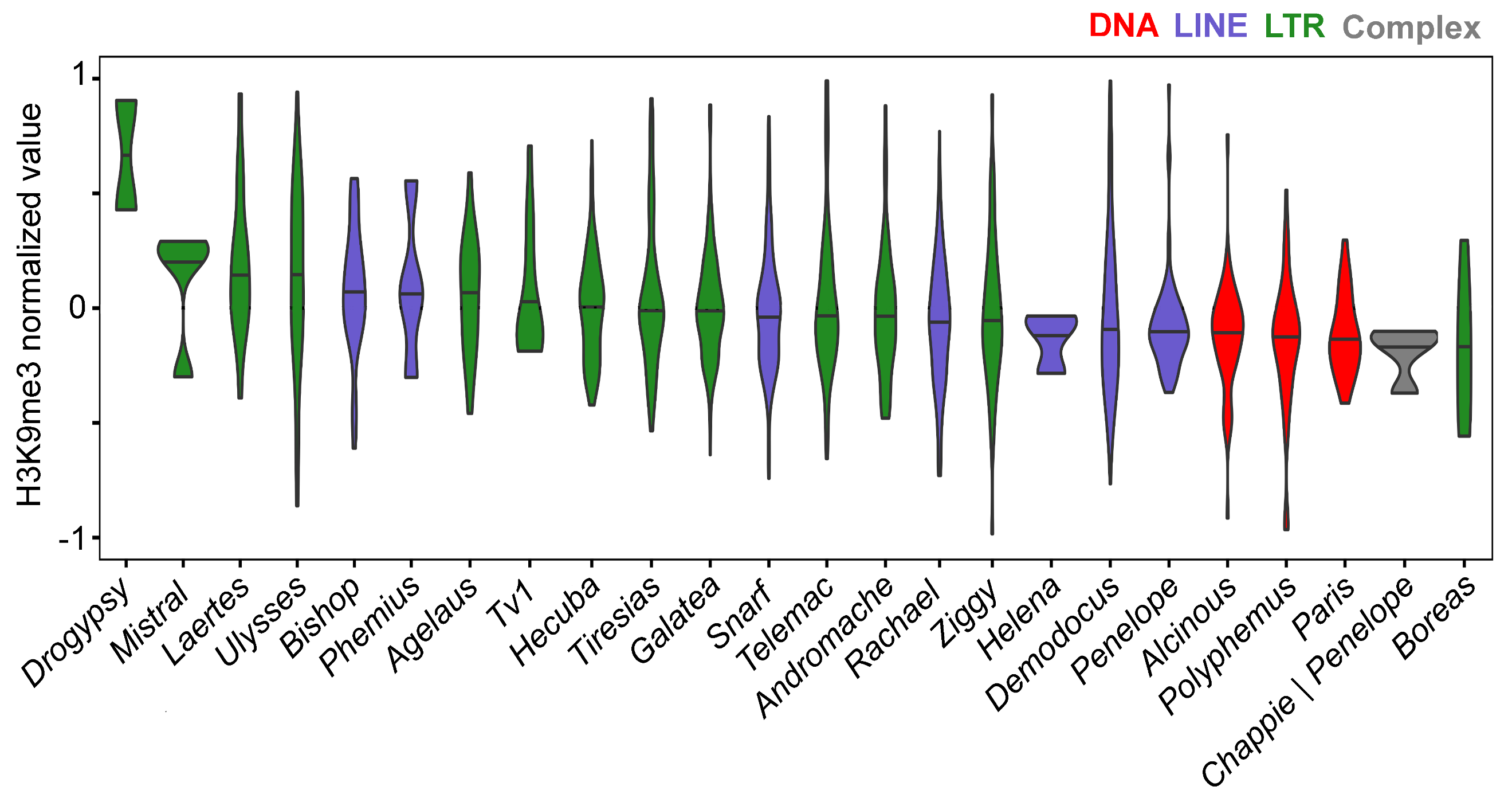


**Supplementary Figure S15.** The magnitude of H3K9me3 enrichment induced by TE for different TE families. The data set includes only those TE families displaying H3K9me3 spreading in at least one insertion.

**Extended Materials and Methods**

**Fly stocks and husbandry**

Five *D. virilis* strains were analysed in this study, including three wild-type strains *9* (Batumi, Georgia), *101* (Japan, neutral substrain according to the classification used in hybrid dysgenesis studies) (1), *15010-1051.47* (Hangchow, China), and two laboratory strains with recessive markers, *140* and *160*. Fly stocks of strains *9*, *140* and *101* were obtained from the Stock Center of Koltzov Institute of Developmental Biology RAS (Moscow, Russia). Strain *15010-1051.47* was obtained from the National Drosophila Species Stock Centre at Cornell University (USA). Strain *160* is a long-established strain maintained in our lab and has the capacity to induce hybrid dysgenesis (2). All flies were reared on standard agar-yeast-sugar-raisin medium at a constant temperature regime (25^0^C) until the age of 10–15 days to reach sexual maturity.

**DNA extraction, library preparation, ONT sequencing, and genome assembly**

Due to the expansion of 7 bp satellite DNA in the pericentromeric and centromeric regions of all chromosomes, the genome size of *D. virilis* is unusually large compared to other Drosophila species (~390 Mb) (3). In order to enrich the DNA library by euchromatic DNA sequences and improve the quality of the genome assembly, we took advantage of the isolation of genomic DNA from ovarian tissue characterized by underreplicated centromeric satellite DNA due to polytenization (4). Females of the *160*, *101* and *1051.47* strains were collected for DNA extraction and aged for 10-15 days. Genomic DNA was isolated from thirty pairs of ovaries using high-salt denaturing buffer containing 100 mM Tris-HCl (pH = 8), 10 mM EDTA (pH = 8), 350 mM NaCl, 2% SDS, and 7 M Urea following phenol-chloroform extraction. Before sequencing, we performed a size selection of high molecular weight DNA by depleting short DNA fragments below 10 kb using the Short Read Eliminator Kit (Circulomics, USA).

The preparation of DNA libraries for sequencing was carried out using the 1D ligation SQK-LSK109 kit (Oxford Nanopore Technologies (ONT), UK), by the recommended protocol from ONT. Sequencing was performed on a flow cell FLO-MIN-106D r9.4 (ONT, UK). The result was an average of 9.5 billion bases sequenced, with a median length of sequenced fragments from 20 to 40 kb for different libraries.

The conversion of FAST5 to FASTQ format was carried out by Guppy 6.4.6 using the super accurate config of the flip-flop algorithm (dna_r9.4.1_450bps_sup.cfg). Quality filtering (Phred > 10) was completed by NanoFilt (5). The adapter sequences were then removed using Porechop (6).

Genomes were assembled using Flye v2.9 (with an expected genome size set to 200 Mb) (7). The assembly quality was evaluated using QUAST 5.1 (8), and the presence of universal single-copy orthologs was determined using BUSCO software with BuscoDB version 4 and the Diptera lineage (9). For the reference-based QUAST assessment, we used the genome of *D. virilis* r.1.06 (downloaded from https://flybase.org/), assembled and annotated in the 12 Drosophila genomes project (10). The genome annotation of *D. virilis* genome r.1.06 was transferred to other *D. virilis* genomes using Liftoff (11) with default parameters.

To directly compare the effect of sequencing errors from PacBio and ONT technologies, we aligned the sequenced genomes to a reference genome (strain *9*) using GSAlign (12) to find single-nucleotide polymorphisms (SNPs). VCF files were annotated in R using VariantAnnotation and GenomicFeatures packages (13,14), focusing only on SNPs located within gene loci.

To compare homozygosity and heterozygosity levels between sequencing technologies (PacBio vs. ONT), reads were aligned to the *D. virilis* genome using BWA-MEM (15). The genome of strain *101* was used as reference. The resulting alignments were sorted, and duplicate reads were marked using SAMtools (16). Variant calling was performed with FreeBayes (17), applying filters for a minimum mapping quality of 30, base quality of 15, alternate fraction of 0.01, alternate count of 3, and coverage of 10. Heterozygous SNPs were defined as those with a "0/1" genotype, homozygous SNPs as "1/1", and all others were categorized as ambiguous. Heterozygosity levels were calculated as the proportion of heterozygous SNPs to all called SNPs.

Genome assemblies for strains *101* and *1051.47* are available at NCBI BioProject with the accession number PRJNA1279437. The genomes of *160*, *9,* and *140* strains that had previously been sequenced and assembled (18-20) were retrieved from NCBI (GCA_007989325.2 - strain *160*; GCA_016920725.1 - strain *9*; GCA_050656195.1 - strain *140*).

Note, this study utilized ONT reads from strain *160* to identify *de novo* TE insertions and SNP calling using FreeBayes. It is noteworthy that the chromosomal assembly for this same strain was generated in a previous study using PacBio sequencing data (19).

***De novo* annotation of transposable elements and creation of a curated TE library**

For TE annotation, with a particular focus on LTR elements, we employed the RepeatModeler2 pipeline (21) to analyse each available and newly assembled genome of *D. virilis*, utilising the "-LTRstruct" parameter. The obtained consensus was then filtered to reduce the redundancy of repetitive sequences in the RepeatModeler2 output. This filtering stage included: (1) filtering from non-TE protein coding sequences and other repetitive non-TE related sequences including ribosomal RNA and nuclear mitochondrial pseudogenes (numts), (2) discarding short instances of the longer TEs by CD-HIT (22) with the parameters "*-c 0.8 -n 5 -d 0*", and (3) discarding sequences that are shorter than the open reading frame of the smallest known eukaryotic transposase (Ac-type transposase homolog (*Glomus mosseae*) - 76 aa (228 nt) according to Repbase v27.01 (23). Filtering based solely on the length of the internal protein-coding ORF (steps 2 and 3) would be inappropriate for LTR elements because the consensus model must include the LTRs to be structurally accurate. The inclusion of these LTRs often results in a consensus sequence that is longer than the ORF itself. Therefore, to avoid accidentally discarding real TEs, steps (2) and (3) were not applied to LTR elements. Also, we have discarded sequences that were annotated as 'Unknown' when performing classification of TEs by the RepeatClassifier script implemented in the RepeatModeler2 package since they showed no homology to the known TE families.

Further curation of TEs was carried out by several successive steps. First, we aligned the filtered RepeatModeler2 output to the TE library composed by Erwin et al. (24) and TEs of *D. virilis* annotated in RepBase v27.01 (23) using blastn (25). According to pairwise alignment, shorter sequences that aligned to longer ones (coverage not less than 50%) were considered to have no value for the reconstruction of full copies of TEs and were therefore discarded. Longer sequences or sequences that were absent in one of the libraries were subjected to further curation. The second step includes manual curation to extend the TE sequences and polish their edges using the guidelines (26,27) and scripts written by Storer et al. (28). In summary, the consensus for each TE model was reconstructed by repeatedly aligning the sequences of all copies of each element found in the genome by running the script “AlignAndCallConsensus.pl”, which is implemented in the RepeatModeler2 package. In many cases, TE models comprise a subfamily structure that includes deletion and recombination products differing in length. To optimise the sorting of sequences by length and identity, we applied the script “ClusterPartialMatchingSubs.pl”. This step was carried out sequentially for all assembled genomes of *D. virilis*. Finally, to obtain the canonical sequences of TEs, a finishing round of manual curation was performed, including iterated multiple sequence alignment of full-length copies (coverage ≥ 90% according to blastn) of each element found in all genomes studied so far. At this step, careful attention was paid to the completeness of open reading frames (ORFs) according to protein domain structures intrinsic to TE subclasses. Protein domains of TEs were annotated using the conserved domain database (CDD) (29). The resulting library consists of 100 TEs, including 14 DNA, 5 RC, 30 LINE, and 50 LTR elements, and 1 *Penelope*-like element (Supplementary File 1). To avoid confusion, the names of TE described in this study and the corresponding homologous TE families in RepBase and Erwin et al. (24) are provided in the Supplementary File 2.

**TE analysis**

To screen genomes for TE insertions, we used RepeatMasker software (v 4.1.0) (30) in RMBlast mode. Given that some TEs feature low-complexity regions, we implemented two filtering criteria on RepeatMasker output to prevent erroneous identification of low-complexity genomic regions as TE insertions. The standard '80-80-80' rule is effective for identifying full-length TEs but is often too stringent for capturing the short, fragmented copies that dominate eukaryotic genomes. To achieve a comprehensive annotation of all TE-derived sequences, including the short, degenerate fragments that are prevalent in assembled genomes we first assign individual elements to families. Each TE insertion must demonstrate greater than 90 percent identity over more than 50 bp of sequence. Second, each TE insertion must not overlap with any region of low complexity or simple repeat by more than 50 percent of the TE insertion length. Additionally, the output of RepeatMasker was then filtered to include only TE insertions over 50 nt.

Due to the occurrence of indels, the blast algorithm may interpret a single TE copy as multiple closely spaced insertions. To reduce fragmentation of full-length TE copies in the genome, we parsed RepeatMasker output files (.out) with the script “one_code_to_find_them_all.pl” (31) with the parameter “--insert 11”. An insert size of 11 was selected to merge fragments of a single element that fall within the default word length of the RMBlast search engine applied in RepeatMasker.

To estimate the divergence time of TEs, we calculated the Kimura distance using the RepeatMasker output (.align) and executing the script “calcDivergenceFromAlign.pl” implemented in the RepeatMasker package. The source code of the script was modified in order to return the Kimura distances for each TE family, instead of superfamilies. The resulting output file was then parsed, and the Kimura distance values for each TE insertion were collected. We then converted the Kimura values into a matrix, with each row representing a single TE family and each column representing a specific Kimura distance value. The cell values were filled with the number of bases in the genome covered by each TE family corresponding to a particular Kimura value, normalized to each genome size. The matrix was Z-transformed, and the silhouette method, implemented in the fviz_nbclust function from the factoextra R package (32), was used to determine the optimal number of TE clusters, according to the Kimura distance. The Z-transformation of the data matrix was then processed using the k-means algorithm, resulting in the identification of three distinct TE clusters. This method was chosen over direct peak-calling to classify entire TE families based on their full divergence profile, which is particularly useful for families with broad distributions.

To assess the transposition capacity of each TE insertion, we analysed the ORFs of the identified TE insertions. Initially, all intact canonical TE sequences were analysed using the ExPasy tool (33), followed by manual curation. The length of all non-overlapping ORFs was then obtained. Then, the Biostrings package was used to extract sequences of all TE insertions in the genome identified by RepeatMasker. Each TE sequence was further processed using the findORFs function of the ORFik R package (34), which extracts the coordinates of all non-overlapping ORFs. The translate function of the Biostrings package was used to identify ORFs starting with ATG codons. Finally, to avoid potential frame shifts in TE proteins, all protein sequences obtained were aligned with CD-HIT using a 90%-similarity cutoff (*command: cd-hit -c 0.9 -d 500 -i proteins.fasta -o clusters*). TE insertions with a protein length of at least 90% were designated as full-length TE copies with complete ORFs of a given TE family.

Pericentric chromatin regions of the chromosomes of the strain *160* were manually annotated in the Integrative Genome Viewer (IGV) (35) using ChIP sequencing data of the heterochromatic mark H3K9me3 in ovarian tissue (36). As stated in Riddle et al. (37), the presence of a sharp shift in the density of H3K9me3 was indicative of a boundary between euchromatin and heterochromatin.

Phylogenetic analysis was performed based on the alignment of ORFs that encode the transposase domain for DNA transposons, and ORFs that encode the EN and RT domains for retroelements. Multiple sequence alignment was performed using the MAFFT algorithm (38) . To remove unaligned blocks, we used the trimAl tool (39). The selection of best-fit models of amino acid replacement was performed using ProtTest 3 (40). Phylogenetic trees were inferred using the maximum likelihood method with MEGA 11 software (41). Visualisation was performed using iTOL v6 (42).

The ggplot2 package (43) was utilised to visualise the distribution of TEs across the genome.

**Identification of horizontal transfer of TEs**

Horizontal transfer of TEs is indicated when there is near identity in TE sequence between two species that are divergent (44,45). We thus investigated whether TEs identified in *D. virilis* strains were present in relatives at levels of identity beyond that which is expected by vertical transmission of the TE since the common ancestor with *D. virilis*. This was achieved by using publicly available genome assemblies of *D. virilis* group species extracted from NCBI. The analyzed species included: *Drosophila novamexicana* (GCF_003285875.2), *Drosophila americana* (GCA_030788265.1), *Drosophila americana texana* (GCA_019972375.1), *Drosophila ezoana* (GCA_035045725.1), *Drosophila borealis* (GCA_035045885.1), *Drosophila flavomontana* (GCA_040285225.1), *Drosophila lacicola* (GCA_035045355.1), and *Drosophila montana* (JAMZDK01). To identify the best-matching TE sequences in the genomes, we performed a blastn search using all *D. virilis* TE sequences as queries. The search was conducted with "best-hit" parameters (*-max_target_seqs 1, -best_hit_score_edge 0.05, -best_hit_overhang 0.25*) to ensure high-confidence matches. The resulting hits were filtered to retain only those covering ≥ 90% of the canonical TE sequence length and exhibiting ≥ 99.5% sequence identity.

To estimate the neutral substitution rate, we calculated the synonymous substitution rate (dS) for single-copy orthologous genes and TE ORFs between *D. virilis* and other species in the group. We identified a conserved set of 50 single-copy orthologs using the BUSCO database (9). For each of these BUSCO genes and for each TE family, we generated multiple sequence alignments with MAFFT (38) and calculated dS values using the MSA2dist R package (46). Finally, we compared the dS distributions of protein-coding genes and TEs using a Mann-Whitney U-test, with statistical significance assessed by FDR correction.

**Identification of non-reference TE insertion**

To identify TE insertions that differ between the studied *D. virilis* strains, we applied the TELR tool which allows the identification, assembly, and allele frequency estimation of non-reference TE insertions using long-read sequencing data (47). TELR pipeline was executed with default parameters using the chromosomal assembly of strain *160* as a reference and genomic reads for strains *9*, *101*, *140,* and *1057.41* obtained by ONT as a query. To identify non-reference TE insertions in strain *160*, we used TELR with ONT reads from *160* as a query and the *1057.41* assembly as a reference. Then, the BED file containing insertion loci was converted to gene transfer format (GTF) and used as a reference GTF for Liftoff (run with default parameters, including the *-polish* flag) to transfer features back to the genome of *160*. The coordinates of each feature in the GTF file were extended by 100 nt downstream and upstream of the TE insertion coordinate before TE annotation transfer. We retained only those TE insertions that showed full overlap with the RepeatMasker annotations of the genome of strain *160*.

To retrieve unique TE insertions for each strain studied, we consistently overlap TE insertions coordinates obtained by TELR using bedtools intersect (48). As unique TE insertions, we consider only those TE insertions that do not overlap between the studied strains, 5 kb upstream and 5 kb downstream of the TE insertion coordinates.

The distribution of TE insertions among the genomic features was estimated using the annotatePeak function from the ChIPSeeker R package (49).

**RNA-seq experiments and TE expression analysis**

Total RNA for RNA-seq was extracted from the ovaries of flies aged 10-15 days using the Extract RNA reagent (Evrogen, Russia). The concentration of RNA was measured with a Qubit Fluorometer (Invitrogen, USA). The quality of RNA was then determined using an Agilent BioAnalyzer 2100 with an RNA 6000 nano kit. The RNA Integrity Number (RIN) of all RNA samples taken for mRNA libraries preparation was not less than 8. Poly(A)-containing RNA was isolated using NEBNext Poly(A) mRNA Magnetic Isolation Module (New England Biolabs, USA), and libraries for RNA-seq were prepared using the NEBNext Ultra II Directional RNA Library Prep Kit for Illumina (New England Biolabs, USA) according to the manufacturer's guidelines. Experiments were performed in two biological replicates for each *D. virilis* strain. Paired-end sequencing (50+50 nucleotides) was conducted on an Illumina NextSeq 2000 platform.

Pre-processing of sequenced reads, including all NGS experiments, was performed by TrimGalore (https://github.com/FelixKrueger/TrimGalore), including adapter and quality (≥ 20 Phred) trimming. Trimmed RNA-seq reads were aligned to the genome of *160* using STAR 2.7.1a (50). TE expression analysis was conducted with TEtranscripts (51) using the TE annotation file obtained by RepeatMasker and transformed to the GTF format. The counts of RNA-seq reads mapped on the consensus TE sequences were then normalized to TE length and sequencing depth (RPKM, Reads Per Kilobase per Million mapped reads). The resulting values were considered as TE expression levels. Differential gene expression analysis was performed with the edgeR package (52).

**Small RNA-seq experiment and TE-targeting piRNA analysis**

The ovarian small RNA fraction for cloning was separated from total RNA (~ 15 μg) of files aged 10-15 days using 15% polyacrylamide gel electrophoresis containing 8M Urea. Following this, the gel fragments corresponding to the small RNA fraction were excised using chemically synthesised RNA corresponding to 21 and 29 nucleotides as size markers. The cloning of small RNA libraries was then performed using the Illumina TruSeq Small RNA prep kit (Illumina, USA) according to the manufacturer's protocol.

Pre-processed reads were then subjected to subtraction of reads matching all rRNA, tRNA, snRNA, and microRNA sequences of the Drosophila genus. The selected reads were then mapped to the consensus sequences of TEs and the genome of the corresponding *D. virilis* strain using Bowtie (53), with a requirement for a perfect match (0 mismatches). We considered small RNA fractions of 23-29 nt in length as piRNAs (54,55). While this method does not isolate RISC-bound piRNAs specifically, the 23-29 nt size fraction from Drosophila ovaries is highly enriched for piRNAs. This has been confirmed by the characteristic 1U bias and ping-pong signature, which are described in our previously published data (1,55). Analysis of piRNAs, including sorting of sense and antisense piRNAs, and calculation of ping-pong signatures, was carried out by well-described techniques (56), utilising custom scripts written in Python.

**ChIP-seq experiments and enrichment analysis of methylated H3K9**

ChIP analysis was performed as described in Akulenko et al. (57). Briefly, ChIP experiments were performed using a commercial anti-H3K9me3 (ab8898, Abcam, UK) and anti-H3K9me2 (ab1220, Abcam, UK) antibodies. Antibodies were bound to Protein A/G agarose beads (Pierce, Thermo Fisher Scientific, USA). Libraries for sequencing were prepared using the NEBNext Ultra II DNA Library Prep Kit for Illumina (New England Biolabs, USA). The experiment included two biological replicates, and paired-end sequencing (50+50 bp) was conducted on an Illumina NextSeq 2000 platform. For analysis of H3K9me3 enrichment of strains *160* and *9,* we used data (single-end sequencing) published previously (36) and deposited in NCBI GEO under the number GSE59965.

Trimmed sequenced reads were aligned to the genome of *160* with Bowtie2 (58). Aligned reads were sorted by coordinates, filtered of most multi-mapped reads (*samtools view -Sbh -q 10*) and cleaned of duplicates (*samtools rmdup*) using SAMtools (59).

Aligned files in BAM format were converted to BigWig format with a bin size of 10 nt and normalized using the RPGC method (number of reads per bin/scaling factor for 1x average coverage of the genome) using the bamCoverage script (*command: bamCoverage --binSize 10 --normalizeUsing RPGC --effectiveGenomeSize (total length of the genome) --extendReads (for pair-end data)*) implemented in the deepTools2 package (60). Next, input sample values were subtracted from treatment sample values using the bigwigCompare script, followed by calculation of the matrix centred on the TE insertion coordinates (deeptools computeMatrix).

To determine the epigenetic effects of TEs, the bins were extended from 10 bp to 1000 bp and centered between the insertion loci (0-1 kb downstream, 1-2 kb downstream, etc.). To examine the epigenetic impact of the unique TE insertion in five *D. virilis* strains, we consistently used four control strains for each experimental strain. For example, to determine the epigenetic effects of strain *101*'s unique TE insertions, the epigenetic profile of *101* (the experimental group, TE present) was compared to the combined epigenetic profiles of strains *160*, *9*, *140*, and *1051.47* (the mean signal, the control group, TE absent). Statistical significance of H3K9me2 and H3K9me3 enrichment values at each locus between strains was estimated using the Mann-Whitney U test, followed by FDR correction. The TE insertion exhibiting heterochromatin spreading was considered as true if satisfying two criteria: 1) the enrichment of H3K9me2 or H3K9me3 is required to be higher in the experimental group compared to the control group in the defined flanking sequence from the TE insertion; 2) the determined difference is required to be statistically significant (FDR < 0.05).

The chi-squared test was used to assess the association between the presence of heterochromatin spreading as a result of TE insertion at the corresponding genomic feature of TE location (e.g., promoter, exon, intron, etc.) and gene downregulation (FDR < 0.05). Loci with significant enrichment were manually curated using IGV.

**Chromosomal inversion analysis**

To screen the assembled genomes for genomic rearrangements, we performed a pairwise genome-wide alignment using Minimap2 with the asm5 preset (61). The resulting alignment file was visualized using dotPlotly (https://github.com/tpoorten/dotPlotly). The Blastn algorithm was applied to estimate the inversion breakpoints more accurately.

The polytene chromosomes of the salivary glands of the F1 hybrid from the cross between the *1051.47* and *9* strains were prepared and stained according to the following method (62).

PCR validation of the inversion breakpoints was performed using LongAmp Taq DNA Polymerase (New England Biolabs, USA). The sequences of the primers used are as follows: P1 – GCGTTTGTTCGCCAAAGCG; P2 – GTCCTGCCAGTTGTTTCAGTTTG; P3 – CATTATAATCTTGCAGCTGCC; P4 – CCGTTAAACTGAATTATGCGCCATG. For the determination of the proximal inversion breakpoint in the genome of *1051.47,* we have used a combination of the primer pairs P1 and P3, and for the distal inversion breakpoint, P2 and P4.

**References**

1. Funikov, S.Y., Kulikova, D.A., Krasnov, G.S., Rezvykh, A.P., Chuvakova, L.N., Shostak, N.G., Zelentsova, E.S., Blumenstiel, J.P. and Evgen'ev, M.B. (2018) Spontaneous gain of susceptibility suggests a novel mechanism of resistance to hybrid dysgenesis in Drosophila virilis. *PLoS genetics*, **14**, e1007400.

2. Lozovskaya, E.R., Scheinker, V.S. and Evgen'ev, M.B. (1990) A hybrid dysgenesis syndrome in Drosophila virilis. *Genetics*, **126**, 619-623.

3. Bosco, G., Campbell, P., Leiva-Neto, J.T. and Markow, T.A. (2007) Analysis of Drosophila species genome size and satellite DNA content reveals significant differences among strains as well as between species. *Genetics*, **177**, 1277-1290.

4. Belyaeva, E.S., Zhimulev, I.F., Volkova, E.I., Alekseyenko, A.A., Moshkin, Y.M. and Koryakov, D.E. (1998) Su(UR)ES: a gene suppressing DNA underreplication in intercalary and pericentric heterochromatin of Drosophila melanogaster polytene chromosomes. *Proceedings of the National Academy of Sciences of the United States of America*, **95**, 7532-7537.

5. De Coster, W., D'Hert, S., Schultz, D.T., Cruts, M. and Van Broeckhoven, C. (2018) NanoPack: visualizing and processing long-read sequencing data. *Bioinformatics (Oxford, England)*, **34**, 2666-2669.

6. Bonenfant, Q., Noé, L. and Touzet, H. (2023) Porechop_ABI: discovering unknown adapters in Oxford Nanopore Technology sequencing reads for downstream trimming. *Bioinformatics advances*, **3**, vbac085.

7. Kolmogorov, M., Yuan, J., Lin, Y. and Pevzner, P.A. (2019) Assembly of long, error-prone reads using repeat graphs. *Nature biotechnology*, **37**, 540-546.

8. Gurevich, A., Saveliev, V., Vyahhi, N. and Tesler, G. (2013) QUAST: quality assessment tool for genome assemblies. *Bioinformatics (Oxford, England)*, **29**, 1072-1075.

9. Simão, F.A., Waterhouse, R.M., Ioannidis, P., Kriventseva, E.V. and Zdobnov, E.M. (2015) BUSCO: assessing genome assembly and annotation completeness with single-copy orthologs. *Bioinformatics (Oxford, England)*, **31**, 3210-3212.

10. Clark, A.G., Eisen, M.B., Smith, D.R., Bergman, C.M., Oliver, B., Markow, T.A., Kaufman, T.C., Kellis, M., Gelbart, W., Iyer, V.N. *et al.* (2007) Evolution of genes and genomes on the Drosophila phylogeny. *Nature*, **450**, 203-218.

11. Shumate, A. and Salzberg, S.L. (2021) Liftoff: accurate mapping of gene annotations. *Bioinformatics (Oxford, England)*, **37**, 1639-1643.

12. Lin, H.N. and Hsu, W.L. (2020) GSAlign: an efficient sequence alignment tool for intra-species genomes. *BMC genomics*, **21**, 182.

13. Lawrence, M., Huber, W., Pagès, H., Aboyoun, P., Carlson, M., Gentleman, R., Morgan, M.T. and Carey, V.J. (2013) Software for computing and annotating genomic ranges. *PLoS computational biology*, **9**, e1003118.

14. Obenchain, V., Lawrence, M., Carey, V., Gogarten, S., Shannon, P. and Morgan, M. (2014) VariantAnnotation: a Bioconductor package for exploration and annotation of genetic variants. *Bioinformatics (Oxford, England)*, **30**, 2076-2078.

15. Li, H. and Durbin, R. (2009) Fast and accurate short read alignment with Burrows-Wheeler transform. *Bioinformatics (Oxford, England)*, **25**, 1754-1760.

16. Li, H., Handsaker, B., Wysoker, A., Fennell, T., Ruan, J., Homer, N., Marth, G., Abecasis, G. and Durbin, R. (2009) The Sequence Alignment/Map format and SAMtools. *Bioinformatics (Oxford, England)*, **25**, 2078-2079.

17. Garrison, E. and Marth, G. (2012) Haplotype-based variant detection from short-read sequencing. *arXiv preprint arXiv*.

18. Bespalova, A.V., Kulikova, D.A., Zelentsova, E.S., Rezvykh, A.P., Guseva, I.O., Dorador, A.P., Evgen'ev, M.B. and Funikov, S.Y. (2025) Paramutation-Like Behavior of Genic piRNA-Producing Loci in Drosophila virilis. *International journal of molecular sciences*, **26**.

19. Hemmer, L.W., Dias, G.B., Smith, B., Van Vaerenberghe, K., Howard, A., Bergman, C.M. and Blumenstiel, J.P. (2020) Hybrid dysgenesis in Drosophila virilis results in clusters of mitotic recombination and loss-of-heterozygosity but leaves meiotic recombination unaltered. *Mobile DNA*, **11**, 10.

20. Rezvykh, A.P., Funikov, S.Y., Protsenko, L.A., Kulikova, D.A., Zelentsova, E.S., Chuvakova, L.N., Blumenstiel, J.P. and Evgen'ev, M.B. (2021) Evolutionary Dynamics of the Pericentromeric Heterochromatin in Drosophila virilis and Related Species. *Genes*, **12**.

21. Flynn, J.M., Hubley, R., Goubert, C., Rosen, J., Clark, A.G., Feschotte, C. and Smit, A.F. (2020) RepeatModeler2 for automated genomic discovery of transposable element families. *Proceedings of the National Academy of Sciences of the United States of America*, **117**, 9451-9457.

22. Fu, L., Niu, B., Zhu, Z., Wu, S. and Li, W. (2012) CD-HIT: accelerated for clustering the next-generation sequencing data. *Bioinformatics (Oxford, England)*, **28**, 3150-3152.

23. Bao, W., Kojima, K.K. and Kohany, O. (2015) Repbase Update, a database of repetitive elements in eukaryotic genomes. *Mobile DNA*, **6**, 11.

24. Erwin, A.A., Galdos, M.A., Wickersheim, M.L., Harrison, C.C., Marr, K.D., Colicchio, J.M. and Blumenstiel, J.P. (2015) piRNAs Are Associated with Diverse Transgenerational Effects on Gene and Transposon Expression in a Hybrid Dysgenic Syndrome of D. virilis. *PLoS genetics*, **11**, e1005332.

25. Camacho, C., Coulouris, G., Avagyan, V., Ma, N., Papadopoulos, J., Bealer, K. and Madden, T.L. (2009) BLAST+: architecture and applications. *BMC bioinformatics*, **10**, 421.

26. Goubert, C., Craig, R.J., Bilat, A.F., Peona, V., Vogan, A.A. and Protasio, A.V. (2022) A beginner's guide to manual curation of transposable elements. *Mobile DNA*, **13**, 7.

27. Rodriguez, F. and Arkhipova, I.R. (2023) An Overview of Best Practices for Transposable Element Identification, Classification, and Annotation in Eukaryotic Genomes. *Methods in molecular biology (Clifton, N.J.)*, **2607**, 1-23.

28. Storer, J.M., Hubley, R., Rosen, J. and Smit, A.F.A. (2021) Curation Guidelines for de novo Generated Transposable Element Families. *Current protocols*, **1**, e154.

29. Wang, J., Chitsaz, F., Derbyshire, M.K., Gonzales, N.R., Gwadz, M., Lu, S., Marchler, G.H., Song, J.S., Thanki, N., Yamashita, R.A. *et al.* (2023) The conserved domain database in 2023. *Nucleic acids research*, **51**, D384-d388.

30. Smit, A., Hubley, R & Green, P. (2013-2015) RepeatMasker Open-4.0. [*http://www.repeatmasker.org*](http://www.repeatmasker.org).

31. Bailly-Bechet, M., Haudry, A. and Lerat, E. (2014) “One code to find them all”: a perl tool to conveniently parse RepeatMasker output files. *Mobile DNA*, **5**, 13.

32. Kassambara, A. and Mundt, F. (2020) Factoextra: Extract and Visualize the Results of Multivariate Data Analyses. R Package Version 1.0.7.

33. Duvaud, S., Gabella, C., Lisacek, F., Stockinger, H., Ioannidis, V. and Durinx, C. (2021) Expasy, the Swiss Bioinformatics Resource Portal, as designed by its users. *Nucleic acids research*, **49**, W216-w227.

34. Tjeldnes, H., Labun, K., Torres Cleuren, Y., Chyżyńska, K., Świrski, M. and Valen, E. (2021) ORFik: a comprehensive R toolkit for the analysis of translation. *BMC bioinformatics*, **22**, 336.

35. Robinson, J.T., Thorvaldsdóttir, H., Winckler, W., Guttman, M., Lander, E.S., Getz, G. and Mesirov, J.P. (2011) Integrative genomics viewer. *Nature biotechnology*, **29**, 24-26.

36. Le Thomas, A., Marinov, G.K. and Aravin, A.A. (2014) A transgenerational process defines piRNA biogenesis in Drosophila virilis. *Cell reports*, **8**, 1617-1623.

37. Riddle, N.C., Minoda, A., Kharchenko, P.V., Alekseyenko, A.A., Schwartz, Y.B., Tolstorukov, M.Y., Gorchakov, A.A., Jaffe, J.D., Kennedy, C., Linder-Basso, D. *et al.* (2011) Plasticity in patterns of histone modifications and chromosomal proteins in Drosophila heterochromatin. *Genome research*, **21**, 147-163.

38. Katoh, K. and Standley, D.M. (2013) MAFFT multiple sequence alignment software version 7: improvements in performance and usability. *Molecular biology and evolution*, **30**, 772-780.

39. Capella-Gutiérrez, S., Silla-Martínez, J.M. and Gabaldón, T. (2009) trimAl: a tool for automated alignment trimming in large-scale phylogenetic analyses. *Bioinformatics (Oxford, England)*, **25**, 1972-1973.

40. Darriba, D., Taboada, G.L., Doallo, R. and Posada, D. (2011) ProtTest 3: fast selection of best-fit models of protein evolution. *Bioinformatics (Oxford, England)*, **27**, 1164-1165.

41. Tamura, K., Stecher, G. and Kumar, S. (2021) MEGA11: Molecular Evolutionary Genetics Analysis Version 11. *Molecular biology and evolution*, **38**, 3022-3027.

42. Letunic, I. and Bork, P. (2024) Interactive Tree of Life (iTOL) v6: recent updates to the phylogenetic tree display and annotation tool. *Nucleic acids research*, **52**, W78-w82.

43. Wickham, H. (2016) ggplot2: Elegant Graphics for Data Analysis. *Springer-Verlag New York*.

44. Daniels, S.B., Peterson, K.R., Strausbaugh, L.D., Kidwell, M.G. and Chovnick, A. (1990) Evidence for horizontal transmission of the P transposable element between Drosophila species. *Genetics*, **124**, 339-355.

45. Peccoud, J., Loiseau, V., Cordaux, R. and Gilbert, C. (2017) Massive horizontal transfer of transposable elements in insects. *Proceedings of the National Academy of Sciences of the United States of America*, **114**, 4721-4726.

46. K, U. (2025), Vol. R package version 1.14.0.

47. Han, S., Dias, G.B., Basting, P.J., Viswanatha, R., Perrimon, N. and Bergman, C.M. (2022) Local assembly of long reads enables phylogenomics of transposable elements in a polyploid cell line. *Nucleic acids research*, **50**, e124.

48. Quinlan, A.R. and Hall, I.M. (2010) BEDTools: a flexible suite of utilities for comparing genomic features. *Bioinformatics (Oxford, England)*, **26**, 841-842.

49. Wang, Q., Li, M., Wu, T., Zhan, L., Li, L., Chen, M., Xie, W., Xie, Z., Hu, E., Xu, S. *et al.* (2022) Exploring Epigenomic Datasets by ChIPseeker. *Current protocols*, **2**, e585.

50. Dobin, A., Davis, C.A., Schlesinger, F., Drenkow, J., Zaleski, C., Jha, S., Batut, P., Chaisson, M. and Gingeras, T.R. (2013) STAR: ultrafast universal RNA-seq aligner. *Bioinformatics (Oxford, England)*, **29**, 15-21.

51. Jin, Y., Tam, O.H., Paniagua, E. and Hammell, M. (2015) TEtranscripts: a package for including transposable elements in differential expression analysis of RNA-seq datasets. *Bioinformatics (Oxford, England)*, **31**, 3593-3599.

52. Robinson, M.D., McCarthy, D.J. and Smyth, G.K. (2010) edgeR: a Bioconductor package for differential expression analysis of digital gene expression data. *Bioinformatics (Oxford, England)*, **26**, 139-140.

53. Langmead, B., Trapnell, C., Pop, M. and Salzberg, S.L. (2009) Ultrafast and memory-efficient alignment of short DNA sequences to the human genome. *Genome biology*, **10**, R25.

54. Brennecke, J., Aravin, A.A., Stark, A., Dus, M., Kellis, M., Sachidanandam, R. and Hannon, G.J. (2007) Discrete small RNA-generating loci as master regulators of transposon activity in Drosophila. *Cell*, **128**, 1089-1103.

55. Rozhkov, N.V., Aravin, A.A., Zelentsova, E.S., Schostak, N.G., Sachidanandam, R., McCombie, W.R., Hannon, G.J. and Evgen'ev, M.B. (2010) Small RNA-based silencing strategies for transposons in the process of invading Drosophila species. *RNA (New York, N.Y.)*, **16**, 1634-1645.

56. Nicholas F. Parrish, Y.W.I. (2022) *piRNA*. 1 ed. Humana New York, NY.

57. Akulenko, N., Ryazansky, S., Morgunova, V., Komarov, P.A., Olovnikov, I., Vaury, C., Jensen, S. and Kalmykova, A. (2018) Transcriptional and chromatin changes accompanying de novo formation of transgenic piRNA clusters. *RNA (New York, N.Y.)*, **24**, 574-584.

58. Langmead, B. and Salzberg, S.L. (2012) Fast gapped-read alignment with Bowtie 2. *Nature methods*, **9**, 357-359.

59. Danecek, P., Bonfield, J.K., Liddle, J., Marshall, J., Ohan, V., Pollard, M.O., Whitwham, A., Keane, T., McCarthy, S.A., Davies, R.M. *et al.* (2021) Twelve years of SAMtools and BCFtools. *GigaScience*, **10**.

60. Ramírez, F., Ryan, D.P., Grüning, B., Bhardwaj, V., Kilpert, F., Richter, A.S., Heyne, S., Dündar, F. and Manke, T. (2016) deepTools2: a next generation web server for deep-sequencing data analysis. *Nucleic acids research*, **44**, W160-165.

61. Li, H. (2018) Minimap2: pairwise alignment for nucleotide sequences. *Bioinformatics (Oxford, England)*, **34**, 3094-3100.

62. Henderson, D.S. (2004) *Drosophila Cytogenetics Protocols*. Humana Totowa, NJ.
